# Cancer-associated fibroblasts promote drug resistance in *ALK*-driven lung adenocarcinoma cells by upregulating lipid biosynthesis

**DOI:** 10.1186/s40170-025-00400-7

**Published:** 2025-06-16

**Authors:** Ann-Kathrin Daum, Lisa Schlicker, Marc A. Schneider, Thomas Muley, Ursula Klingmüller, Almut Schulze, Michael Thomas, Petros Christopoulos, Holger Sültmann

**Affiliations:** 1 Division of Cancer Genome Research, German Cancer Research Center (DKFZ), German Cancer Consortium (DKTK), National Center for Tumor Diseases (NCT), Im Neuenheimer Feld 280, 69120 Heidelberg, Germany; 2 German Center for Lung Research (DZL), TLRC Heidelberg, Heidelberg, Germany; 3 Medical Faculty, Heidelberg University, Heidelberg, Germany; 4 Division of Tumor Metabolism and Microenvironment, German Cancer Research Center (DKFZ), Heidelberg, Germany; 5 Proteomics Core Facility, German Cancer Research Center (DKFZ), Heidelberg, Germany; 6 Translational Research Unit, Thoraxklinik at Heidelberg University Hospital, Heidelberg, Germany; 7 Division Systems Biology of Signal Transduction, German Cancer Research Center (DKFZ), Heidelberg, Germany; 8 Department of Oncology, Thoraxklinik at Heidelberg University Hospital, Heidelberg, Germany

**Keywords:** Lung adenocarcinoma, EML4-ALK, Cancer-associated fibroblasts, Therapy resistance, Lipid metabolism, 3D cell culture

## Abstract

**Background:**

Targeted therapy interventions using tyrosine kinase inhibitors (TKIs) provide encouraging treatment responses in patients with *ALK*-rearranged lung adenocarcinomas, yet resistance occurs almost inevitably. In addition to tumor cell-intrinsic resistance mechanisms, accumulating evidence suggests that cancer-associated fibroblasts (CAFs) within the tumor microenvironment contribute to therapy resistance. This study aimed to investigate CAF-driven molecular networks that shape the therapeutic susceptibility of *ALK*-driven lung adenocarcinoma cells.

**Methods:**

Three-dimensional (3D) spheroid co-cultures comprising *ALK*-rearranged lung adenocarcinoma cells and CAFs were utilized to model the tumor microenvironment. Single-cell RNA sequencing was performed to uncover transcriptional differences between TKI-treated homotypic and heterotypic spheroids. Functional assays assessed the effects of CAF-conditioned medium and CAF-secreted factors on tumor cell survival, proliferation, lipid metabolism, and downstream AKT signaling. The therapeutic potential of targeting metabolic vulnerabilities was evaluated using pharmacological inhibition of lipid metabolism and by ferroptosis induction.

**Results:**

CAFs significantly diminished the apoptotic response of lung tumor cells to ALK inhibitors while simultaneously enhancing their proliferative capacity. Single-cell RNA sequencing identified lipogenesis-associated genes as a key transcriptional difference between TKI-treated homotypic and heterotypic lung tumor spheroids. CAF-conditioned medium and the CAF-secreted factors HGF and NRG1 activated AKT signaling in 3D-cultured ALK-rearranged lung tumor cells, leading to increased de novo lipogenesis and suppression of lipid peroxidation. These metabolic adaptations were critical for promoting tumor cell survival and fostering therapy resistance. Notably, both dual inhibition of ALK and the lipid-regulatory factor SREBP-1, as well as co-treatment with ferroptosis inducers such as erastin or RSL3, effectively disrupted the CAF-driven metabolic-supportive niche and restored sensitivity of resistant lung tumor spheroids to ALK inhibition.

**Conclusions:**

This study highlights a critical role for CAFs in mediating resistance to ALK-TKIs by reprogramming lipid metabolism in ALK-rearranged lung cancer cells. It suggests that targeting these metabolic vulnerabilities, particularly through inhibition of lipid metabolism or induction of ferroptosis, could provide a novel therapeutic approach to overcome resistance and improve patient outcomes.

**Supplementary Information:**

The online version contains supplementary material available at 10.1186/s40170-025-00400-7.

## Background

Lung adenocarcinomas, the most prevalent non-small cell lung cancer (NSCLC) subtype, can be stratified into different molecular subgroups on the basis of recurrent genomic alterations. Among these, rearrangements of the *ALK* gene occur in approximately 6% of diagnosed cases [1]. Crizotinib was the first approved tyrosine kinase inhibitor (TKI), which led to remarkable responses in patients with advanced ALK + NSCLC compared to standard chemotherapy [2, 3]. Moreover, the next-generation ALK inhibitors (ALKis) brigatinib, alectinib and lorlatinib have become the preferred first-line treatment options because their systemic and intracranial efficacy is superior to that of crizotinib in head-to-head randomized trials [4]. However, the majority of patients who initially respond to these targeted drugs experience disease progression due to the emergence of drug resistance [5]. This is caused by either genetic factors, i.e., secondary mutations in the ALK kinase domain or amplification of the ALK locus, with tumor cells still being ALK dependent, or nongenetic factors, i.e., activation of bypass signaling pathways and phenotypic changes [6]. While resistance mutations can be at least partly targeted by sequential treatment interventions [7], the mechanisms of non-genetic resistance due to bypass signaling remain largely unknown.

In addition to tumor cell-intrinsic mechanisms, the tumor microenvironment (TME) effectively shapes the therapeutic vulnerability of cancer cells [8, 9]. In particular, cancer-associated fibroblast (CAF) populations are known contributors to therapy resistance owing to a range of mechanisms, including adhesion- and matrix-based signaling [10], paracrine crosstalk [11], immunosuppression [12] and metabolic reprogramming [13]. The underlying signaling circuitry and connective tissue remodeling disrupt normal tissue homeostasis and promote cancer initiation and malignant progression. For example, paracrine interactions with stromal fibroblasts affect the sensitivity of NSCLC cells to chemotherapy and targeted therapy [14, 15]. However, the TME interactions leading to therapy resistance are not fully understood.

Metabolic reprogramming represents one of the hallmarks of cancer [16]. Mounting evidence also supports aberrant activation of the lipogenesis pathway in human cancers, which is required for membrane biogenesis and energy compensation in highly proliferative cancer cells [17–19]. However, it remains unclear whether this metabolic phenotype is driven exclusively by cancer cell-intrinsic processes or is also modulated by environmental factors, i.e., tumor-stromal interactions. The expression of genes encoding lipogenic enzymes is controlled mainly by the transcription factor family sterol regulatory element-binding proteins (SREBPs) [20–22], which are expressed in three different isoforms (SREBP-1a, SREBP-1c, and SREBP-2). While SREBP-1 is involved primarily in regulating *de novo* lipogenesis, SREBP-2-driven gene activation affects cholesterol uptake and biosynthesis [22–24]. *SREBP* expression and transcription factor activity are tightly regulated by the cellular nutrient status in response to upstream signaling networks (e.g., the PI3K/AKT/mTORC1 pathway) [25–28]. Similarly, receptor tyrosine kinase (RTK) signaling results in the rewiring of lipid metabolism in oncogene-driven cancers [29–32]. In line with this, Chen et al. [33] demonstrated perturbations in lipid metabolism following TKI treatment in EGFR-driven NSCLCs. The same study also revealed that sustained SREBP-1-dependent lipogenesis was a key mediator of resistance to EGFR-targeted therapy, which could be reversed by metabolic interventions involving SREBP-1 inhibition.

Since, to our knowledge, no prior studies have investigated tumor stroma-dependent global gene expression changes in TKI-treated EML4-ALK-positive NSCLC, the aim of our study was to explore tumor stroma-related processes leading to nongenetic resistance in this tumor type. To mimic fibroblast–tumor cell interactions in the TME in vitro, we established spheroid co-culture models of ALK-driven NSCLC cell lines and CAFs. We employed single-cell RNA-sequencing (scRNA-seq) to analyze differential gene expression between TKI-treated homo- and heterotypic lung tumor spheroids. Our analyses led us to focus on the deregulation of tumor cell lipid metabolism evoked by factors in the TME and provide a resource of data for further characterization of nongenetic TKI therapy resistance in these models.

## Materials and methods

### Reagents

The reagents used in the study are listed in Table 1.

**Table:** Table 1

Reagent type (species) or resource	Designation	Source or reference	Identifiers	Additional information
Cell line (*Homo sapiens*)	A549 [CCL-185]	ATCC	RRID: CVCL_0023	Cell line cultured in F12-K Culture medium supplemented with 10% FCS
Cell line (*Homo sapiens*)	NCI-H2228	ATCC	RRID: CVCL_1543	Cell line cultured in RPMI 1640 Culture medium supplemented with 10% FCS
Cell line (*Homo sapiens*)	NCI-H3122	NCI	RRID: CVCL_5160	Cell line cultured in RPMI 1640 Culture medium supplemented with 10% FCS
Cell line (*Homo sapiens*)	MRC-5 [CCL-171]	ATCC	RRID: CVCL_0440	Cell line cultured in EMEM Culture medium supplemented with 10% FCS
Cell line (*Homo sapiens*)	FB1, Primary lung fibroblasts derived from NSCLC adenocarcinoma patient	Thoraxklinik, Heidelberg University Hospital		Cell line cultured in DMEM Culture medium supplemented with 10% FCS
Cell line (*Homo sapiens*)	FB2, Primary lung fibroblasts derived from NSCLC adenocarcinoma patient	Thoraxklinik, Heidelberg University Hospital		Cell line cultured in DMEM Culture medium supplemented with 10% FCS
Antibody	anti-ACLY (rabbit polyclonal)	CST	Cat# 4332, RRID: AB_2223744	WB (1:1000)
Antibody	anti-β-Actin (rabbit monoclonal)	CST	Cat# 4970, RRID: AB_2223172	WB (1:1000)
Antibody	anti-AKT (rabbit polyclonal)	CST	Cat# 9272, RRID: AB_329827	WB (1:1000)
Antibody	anti-pAKT (Ser473, rabbit monoclonal)	CST	Cat# 4060, RRID: AB_2315049	WB (1:1000)
Antibody	anti-αSMA (mouse monoclonal)	Sigma-Aldrich	Cat# A5228, RRID: AB_262054	WB (1:1000)
Antibody	anti-CD326 (EpCAM; mouse monoclonal)	Miltenyi Biotec	Cat# 130-061-101, RRID: AB_2832928	MicroBeads for use in MACS Separation
Antibody	anti-FAP (rabbit monoclonal)	Abcam	Cat# ab207178, RRID: AB_2864720	WB (1:1000)
Antibody	anti-FASN (rabbit monoclonal)	CST	Cat# 3180, RRID: AB_2100796	WB (1:1000)
Antibody	anti-GAPDH (rabbit monoclonal)	CST	Cat# 2118, RRID: AB_561053	WB (1:1000)
Antibody	Anti-HGF (mouse monoclonal)	R&D Systems	Cat# Cat# MAB294, RRID: AB_2279754	Neutralization (1:500)
Antibody	anti-Ki67 eFluor 450 (rat monoclonal)	Thermo Fisher Scientific	Cat# 48-5698-82, RRID: AB_11149124	Flow Cytometry (1:20)
Antibody	Anti-Nrg1-α (goat polyclonal)	R&D Systems	Cat# AF-296-NA, RRID: AB_354453	Neutralization (1:20)
Antibody	Anti-Nrg1-β1 (goat polyclonal)	R&D Systems	Cat# AF-396-NA, RRID: AB_354472	Neutralization (1:20)
Antibody	anti-S6K/p70 (rabbit monoclonal)	CST	Cat# 2708, RRID: AB_390722	WB (1:1000)
Antibody	anti-pS6K/p70 (Thr389, rabbit polyclonal)	CST	Cat# 9205, RRID: AB_330944	WB (1:1000)
Antibody	anti-SREBP-1 (mouse monoclonal)	SantaCruz Biotechnology	Cat# sc-13,551, RRID: AB_628282	WB (1:500)
Sequence-based reagent	ACACA_F	Sigma-Aldrich	PCR primers	TGAACTTCACACAGGTAGTCTGCC
Sequence-based reagent	ACACA_R	Sigma-Aldrich	PCR primers	TGGAACACTCGATGGAGTTTCT
Sequence-based reagent	ACLY_F	Sigma-Aldrich	PCR primers	GAGGCATATCCAGAGGAAGCC
Sequence-based reagent	ACLY_R	Sigma-Aldrich	PCR primers	TCCCTTTGGGGTTCAGCAAG
Sequence-based reagent	ACTA2_F	Sigma-Aldrich	PCR primers	CTGTTCCAGCCATCCTTCAT
Sequence-based reagent	ACTA2_R	Sigma-Aldrich	PCR primers	TCATGATGCTGTTGTAGGTGGT
Sequence-based reagent	ACTB_F	Sigma-Aldrich	PCR primers	CCAACCGCGAGAAGATGA
Sequence-based reagent	ACTB_R	Sigma-Aldrich	PCR primers	CCAGAGGCGTACAGGGATAG
Sequence-based reagent	FAP_F	Sigma-Aldrich	PCR primers	TGGCGATGAACAATATCCTAGA
Sequence-based reagent	FAP_R	Sigma-Aldrich	PCR primers	ATCCGAACAACGGGATTCTT
Sequence-based reagent	FASN_F	Sigma-Aldrich	PCR primers	TCCGAGATTCCATCCTACGC
Sequence-based reagent	FASN_R	Sigma-Aldrich	PCR primers	GCAGCTGTGACACCTTCAGG
Sequence-based reagent	GAPDH_F	Sigma-Aldrich	PCR primers	AGCCACATCGCTCAGACAC
Sequence-based reagent	GAPDH_R	Sigma-Aldrich	PCR primers	GCCCAATACGACCAAATCC
Sequence-based reagent	SCD1_F	Sigma-Aldrich	PCR primers	TAAGTTGGAGACGATGCCCC
Sequence-based reagent	SCD1_R	Sigma-Aldrich	PCR primers	TGGGCCTTCCTTATCCTTGT
Sequence-based reagent	SREBP-1c_F	Sigma-Aldrich	PCR primers	GGAGCCATGGATTGCACTTT
Sequence-based reagent	SREBP-1c_R	Sigma-Aldrich	PCR primers	TCAAATAGGCCAGGGAAGTCA
Peptide, recombinant protein	HGF	R&D Systems	Cat# 294-HG	
Peptide, recombinant protein	NRG1-β1	R&D Systems	Cat# 396-HB	
Peptide, recombinant protein	TGF-β1	PeproTech	Cat# 100-21-10	
Chemical, compound, drug	Brigatinib	TargetMol	Cat# T3621	
Chemical, compound, drug	Erastin	Molnova	Cat# M15083	
Chemical, compound, drug	Everolimus	MCE	Cat# HY-10,218	
Chemical, compound, drug	Fatostatin	TargetMol	Cat# T6832	
Chemical, compound, drug	Ipatasertib	MCE	Cat# HY-15,186	
Chemical, compound, drug	Lorlatinib	TargetMol	Cat# T3061	
Chemical, compound, drug	RSL3	MCE	Cat# HY-100,218 A	
Commercial assay or kit	Annexin V Apoptosis Detection Kit eFluor 450	Thermo Fisher Scientific	Cat# 88-8006-74	
Commercial assay or kit	CellTiter-Glo 3D Cell Viability Assay	Promega	Cat# G9683	
Commercial assay or kit	CellTrace CFSE Cell Proliferation Kit	Thermo Fisher Scientific	Cat# C34554	
Commercial assay or kit	CellTrace Violet Cell Proliferation Kit	Thermo Fisher Scientific	Cat# C34557	
Commercial assay or kit	Chromium Single Cell 3’ Reagent Kit	10x Genomics	Cat# 1,000,075	
Commercial assay or kit	Human Premixed Multi-Analyte Kit	R&D Systems	Cat# LXSAHM	
Commercial assay or kit	Human WNT5A(Protein Wnt-5a) ELISA Kit	FineTest	Cat# EH1164	
Software, algorithm	Cell Ranger	10x Genomics	RRID: SCR_017344	Version 3.1.0
Software, algorithm	FACSDiva software	BD Biosciences	RRID: SCR_001456	Version 6.1.3
Software, algorithm	FlowJo	BD Biosciences	RRID: SCR_008520	Version 10.6.2
Software, algorithm	Fiji	PMID:22743772	RRID: SCR_002285	
Software, algorithm	GraphPad Prism 8	GraphPad Software Inc	RRID: SCR_002798	Version 8.4.3
Software, algorithm	R	PMID:18252159	RRID: SCR_001905	Version 3.6.0

### Cell culture

Human lung cancer cells (A549, NCI-H2228) and fetal lung fibroblasts (MRC-5 [CCL-171]) were purchased from ATCC (Manassas, VA, USA). NCI-H3122 cells were obtained from NCI (Bethesda, MD, USA). Human primary fibroblasts (FB1, FB2) derived from NSCLC adenocarcinoma patients were generated and kindly provided by the Translational Research Unit of the Thoraxklinik (Heidelberg, Germany) following written informed consent and approval by the ethics committee of the Medical Faculty Heidelberg (S-270/2001, S-296/2016, and S-435/2019, respectively). Briefly, tumor tissues were minced into small pieces and digested using Liberase DH Research Grade (Roche, Mannheim, Germany). Afterwards, the cells were filtered through 100 μm and 40 μm cell strainers (Corning, NY, USA) and purified via Histopaque (Sigma Aldrich, St. Louis, MO, US). The cells were seeded in DMEM (Thermo Fisher Scientific, Waltham, MA, USA) supplemented with 10% FCS (Thermo Fisher Scientific) until the fibroblasts reached 80% confluency. A549 cells were maintained in F12-K medium (ATCC), NCI-H2228 and NCI-H3122 cells in RPMI 1640 medium (Thermo Fisher Scientific), MRC-5 cells in EMEM (Thermo Fisher Scientific), and the primary fibroblast lines FB1 and FB2 in DMEM (Thermo Fisher Scientific), all supplemented with 10% FCS (Thermo Fisher Scientific). The cell lines and primary cells were propagated at 37 °C in a humidified atmosphere with 5% CO_2_ and maintained for no more than 8–15 passages. The cells were subcultured at 70–80% confluence by harvesting with 0.25% trypsin-EDTA (Thermo Fisher Scientific) and were then suspended to the required cell density for subsequent assays. Cells were routinely monitored for mycoplasma contamination and authenticated based on single-nucleotide polymorphism (SNP) profiling.

### CAF transdifferentiation

Fibroblasts were seeded 24 h prior to TGF-β1 treatment. The next day, the cells were serum starved for 8 h with growth medium containing 0.5% FCS. Next, TGF-β1 made up in serum low-medium (final concentration of 2 ng/ml) was added to the cell culture vessel, and the cells were incubated for 72 h. Fibroblasts treated with 0.5% FCS-containing growth medium served as controls.

### 3D cell culture

3D cultures were generated as homo- and heterotypic tumor spheroids. To this end, H2228 and H3122 cells were trypsinized and diluted in ice-cold spheroid medium (DMEM supplemented with GlutaMAX + 25 mM HEPES (Thermo Fisher Scientific) + 10% FCS)). A 50 µl volume of cell suspension corresponding to 2,000 cells was added to each well of a round-bottom ultra-low attachment (ULA) 96-well plate (Corning) followed by centrifugation at 300 × g for 5 min. Adequate spheroid formation was initiated by adding 50 µl of ice-cold spheroid medium additives to the corresponding tumor cell-containing wells as follows: H2228 cells were supplemented with 3% growth factor-reduced (GFR)-Matrigel (Corning), while H3122 cells were supplemented with 1% GFR-Matrigel and 10 µg/ml Cultrex 3D Culture Matrix Rat Collagen I (Bio-Techne, Minneapolis, MN, USA). The plates were incubated under standard cell culture conditions for 72 h. To generate heterotypic spheroids, H2228 and H3122 cells were co-seeded with previously activated MRC-5, FB1 and FB2 fibroblasts, respectively. A volume of 100 µl of cell suspension corresponding to 3,000 cells with a tumor cell: fibroblast ratio of 1:6 was added to each well of a round-bottom ULA 96-well plate. The plates were subsequently centrifuged at 300 × g for 5 min and incubated under standard cell culture conditions as described above.

Homotypic fibroblast spheroids were generated utilizing the AggreWell 400 24-well plate (Stemcell Technologies, Vancouver, Canada). The plates were prepared according to the manufacturer’s instructions. Previously (non)activated MRC-5, FB1 and FB2 cells were detached and diluted in spheroid medium to 2.88 × 10^7^ cells/ml. A volume of 500 µl of cell suspension was seeded into each well containing a microwell inlay corresponding to 1,000 cells/microwell. Fibroblast spheroids were grown under standard cell culture conditions for 48 h.

### FB-conditioned media

FB-conditioned media (FB-CM) were made from MRC-5, FB1, and FB2 cells grown as fibroblast spheroids as described above. After 48 h, the conditioned media were collected and centrifuged for 10 min at 1,000 × g to remove the remaining cells and cell debris. The supernatant was immediately stored at -80 °C for later use.

### Spheroid viability

Homotypic spheroids were treated by adding 100 µl of fresh spheroid culture medium supplemented with the indicated agents. The viability of (non)treated tumor spheroids was evaluated using the CellTiter-Glo 3D Cell Viability Assay (Promega, Fitchburg, WI, USA) according to the manufacturer’s instructions, with slight modifications. Briefly, tumor spheroids were transferred with 50 µl culture medium into a new opaque-walled white-bottomed 96-well plate, which was subsequently allowed to equilibrate to RT. An equivalent volume of reagent was added to all the samples, and the plates were vigorously shaken for 5 min at 750 rpm in the dark. Luminescence signals were recorded following incubation for 30 min at RT using an Infinite M200 plate reader (Tecan, Männedorf, Switzerland).

### Spheroid growth

To quantify spheroid size after ALKi treatment without (w/o) and with (w/) CAF-CM, tumor spheroid mono-cultures were grown for 72 h. On the day of treatment, previously generated CAF-CM was thawed on ice and diluted 1:2 with fresh spheroid medium. H2228 and H3122 spheroids were treated with appropriate ALKi concentrations either in diluted CAF-CM or in spheroid medium only. After 72 h of treatment, the spheroid size was assessed via brightfield imaging at 10x magnification using an Axio Observer 7 (Carl Zeiss, Jena, Germany). Twelve representative spheroids from each corresponding sample group were imaged. The spheroid area (µm²) was evaluated using the freehand selection tool of the image analysis software Fiji [34].

### siRNA-mediated knockdown

For gene silencing experiments, Silencer Select siRNAs targeting *HGF* (siHGF_1: s6528, siHGF_2: s6529, siHGF_3: s6530) and *NRG1* (siNRG1_1: s194521, siNRG1_2: s230507, siNRG1_3: s230508) (Thermo Fisher Scientific) were used. A non-targeting siRNA (Silencer Select Negative Control No. 1) and GAPDH-targeting siRNA (Silencer Select GAPDH Positive Control) served as corresponding controls. FB2 fibroblasts were seeded in T75 flasks 24 h prior to transfection to achieve ~ 60% confluence at the time of transfection. Transfections were performed using Lipofectamine RNAiMAX Reagent (Thermo Fisher Scientific) according to the manufacturer’s instructions. Briefly, siRNAs were diluted in Opti-MEM Reduced Serum Medium (Thermo Fisher Scientific) to a final concentration of 10 nM. Separately, Lipofectamine RNAiMAX was diluted in Opti-MEM. The diluted siRNA and Lipofectamine RNAiMAX solutions were then combined and incubated for 5 min at room temperature to allow complex formation. The siRNA-lipid complexes were added dropwise to the cells without removing the existing medium. Cells were incubated under standard culture conditions (37 °C, 5% CO₂) for 72 h before harvest for spheroid formation and generation of FB-CM. Knockdown efficiency was validated by qPCR.

### Magnetic cell separation

Heterotypic spheroids were pooled and dissociated with Accumax Cell Aggregate Dissociation Medium (Thermo Fisher Scientific) via incubation for 8 min at 37 °C, followed by gently pipetting up and down to disrupt cell-cell contacts. Both steps were repeated at least twice until complete dissociation of the spheroids occurred, and the remaining cell clumps were removed by passing the cells through pre-separation filters (20 μm). Tumor cells were separated from the fibroblast population applying magnetic-activated cell sorting (MACS) via CD326 (EpCAM) MicroBeads (Miltenyi Biotec, Bergisch Gladbach, Germany) according to the manufacturer’s instructions.

### Annexin V apoptosis assay

The tumor cells were stained with a 10 µM CellTrace CFSE Cell Proliferation Kit (Thermo Fisher Scientific) according to the manufacturer’s instructions. The stained cells were resuspended in 1 ml of fresh spheroid culture medium and used for homo- and heterotypic spheroid generation as described above. Apoptosis was measured utilizing the Annexin V Apoptosis Detection Kit eFluor 450 assay (Thermo Fisher Scientific) with the Fixable Viability Dye (FVD) eFluor 660 (Thermo Fisher Scientific). Single cells from pooled and dissociated tumor spheroids were suspended in flow cytometry buffer (1X DPBS + 1% BSA) and incubated at 37 °C for 30 min to allow recovery of the cells. The cells were pelleted by centrifugation for 3 min at 300 × g and recovered in 500 µl of FVD-staining solution (FVD diluted 1:1000 in cold 1X DPBS), followed by incubation for 30 min at RT in the dark. Next, the cells were processed with Annexin V staining solution according to the manufacturer’s protocol. The samples were run through a FACSCanto II flow cytometry system (BD Biosciences, San Jose, CA, USA), and the data were analyzed using FlowJo software.

### Cell cycle analysis

After the corresponding treatment, pooled and dissociated tumor spheroids were fixed in 70% ice-cold ethanol and stained with anti-Ki-67 eFluor 450 antibody (1:20). Combined RNaseA treatment and PI staining was performed using the FxCycle PI/RNase Staining Solution (Thermo Fisher Scientific). Data from the gated tumor cells were acquired by running the samples on a FACSCanto II instrument. Cell cycle plots of quiescent (G0-phase) and actively cycling tumor cells (G1-, S-, G2M-phase) were generated with FlowJo software.

### Lipid peroxidation quantification by BODIPY C11 staining

Tumor spheroids were pooled, dissociated and stained with 10 µM BODIPY 581/591 C11 (Thermo Fisher Scientific) for 30 min at 37 °C after the indicated treatment. Labeled cells were washed twice with PBS and subjected to flow cytometry analysis using a FACSCanto II instrument (488 nm excitation). Positive controls were generated by incubating untreated samples with 1 mM H_2_O_2_ for 1 h at 37 °C.

### RNA extraction, cDNA synthesis, and qRT-PCR

Total RNA was extracted from cell lines or pooled tumor spheroids using the miRNeasy Mini Kit (Qiagen, Hilden, Germany) following the manufacturer’s protocol. RNA was reverse-transcribed using the RevertAid H Minus First Strand cDNA Synthesis Kit (Thermo Fisher Scientific) according to the supplier’s instructions. Quantitative real-time PCR (qPCR) was carried out using the LightCycler 480 II system (Roche, Basel, Switzerland) using Universal ProbeLibrary (Roche) or primaQUANT (Steinbrenner, Wiesenbach, Germany) SYBR assays with primers listed in Table 1. Relative gene expression fold changes were calculated employing the 2^(-ΔΔCt)^ method [35], with *ACTB* or *GAPDH* used as internal standards.

### Immunoblot analysis

Whole-cell lysates were collected from single cells or pooled tumor spheroids using RIPA buffer, supplemented with protease/phosphatase inhibitor cocktail (Roche). Protein concentration was measured with Pierce™ BCA Protein Assay Kit (Thermo Fisher Scientific) following the manufacturer’s instructions. Proteins were separated by SDS-PAGE and transferred onto PVDF membranes applying the Trans-Blot Turbo Transfer system (Bio-Rad Laboratories). Membranes were blocked with 5% BSA or 5% NFDM in 1X TBS-T buffer and incubated with specific primary and secondary antibodies as listed in Table 1. The development of chemiluminescent immunoblot signals was carried out with the SuperSignal West Dura Extended Duration Substrate (Thermo Fisher Scientific), and the signals were detected with a ChemiDoc XRS + System (Bio-Rad Laboratories).

### CAF secretome profiling

Simultaneous detection and quantification of proteins and growth factors was conducted using a Magnetic Luminex Assay. Analytes in cell culture supernatants of fibroblast spheroids were tested with the Human Premixed Multi-Analyte Kit (Bio-Techne). All reagents, standards, and samples were prepared as recommended by the manufacturer. Data acquisition was conducted using a Bio-Plex 200 system (Bio-Rad Laboratories), and standard curves were created using the five-parameter logistic (5PL) curve fitting. Each standard and sample was measured in duplicate. Secreted levels of WNT5A in the cell culture supernatants of fibroblast spheroids were quantitatively determined employing the Human WNT5A ELISA Kit (FineTest, Wuhan, China). All reagents, standards, and samples were prepared according to the manufacturer’s instructions. The absorbance of the samples was recorded at 450 nm using the Infinite M200 plate reader (Tecan). The target concentrations were interpolated from a standard curve created from the standards with predefined concentrations. Each sample was measured in duplicate.

### Single-cell RNA-Sequencing by 10x technology

Single-cell suspensions of homo- and heterotypic tumor spheroids were prepared as described previously and pipetted through a 20 µm cell strainer. To obtain barcoded scRNA-seq libraries, samples were processed using the Chromium Single Cell 3’ Reagent Kit v3 (10x Genomics, Pleasanton, CA, USA) according to the manufacturer’s protocol. In brief, a total of 10,000 cells were loaded on a Chromium Single-Cell Controller Instrument (10x Genomics) to generate single-cell Gel Bead-in EMulsions (GEMs). The incorporated mRNA was reverse transcribed into barcoded cDNA and PCR-amplified. This was followed by fragmentation, end repair, A-tailing, and indexing to generate sequencing libraries. The enriched scRNA-seq library constructs were subjected to 28 + 74 bp paired-end sequencing on four lanes on the HiSeq 4000 system (Illumina, San Diego, CA, USA).

### Preprocessing of ScRNA data

Single-cell RNA-sequencing data were obtained as raw demultiplexed .*fastq* files and preprocessed via the Cell Ranger (version 3.1.0) analysis pipeline (10x Genomics). In brief, cellular barcodes were demultiplexed, and sequencing reads were mapped to the human reference genome (hg38/GRCh38). The aligned reads were filtered, and read count matrices were obtained via cell barcode and unique molecular identifier (UMI) counting.

### Secondary analysis using Seurat

Further filtering and clustering analyses of preprocessed single-cell RNA data were performed in R (version 3.6.0) using the ‘Seurat v3’ R package [36]. Loaded expression matrices were filtered to remove genes detected in < 3 cells. Thresholding the number of detected genes and the fraction of mitochondrial reads was performed for quality control measures. Thresholds were determined per dataset by visual inspection of the distributions, and low-quality cells were excluded from further analysis. In total, 66,375 single cells remained for downstream analyses. For all datasets, the gene expression values for each cell were normalized to a log scale with a scale factor of 10,000. Next, only the top 2,000 genes exhibiting the highest variability across cells were considered for further downstream analysis. For the integration of multiple datasets (i.e., control vs. treatment, mono- vs. co-culture), anchor correspondences were identified and allowed to run a single integrated analysis on all cells. Linear regression (‘scaling’) was performed prior to dimensional reduction to eliminate unwanted sources of variation, such as heterogeneity associated with cell cycle stages and mitochondrial contamination. The scaled data was subsequently used as input for PCA, and the top significant principal components were estimated using the elbow plot function. Significant principal components were further used for graph-based clustering into discrete populations. The resolution parameter, which indirectly sets the number of clusters, was empirically evaluated to establish discernible clusters with distinctive marker gene expression. Afterwards, non-linear dimensional reduction using UMAP was run to project the predicted populations in two dimensions for data visualization and exploitation purposes. To identify marker genes, each cluster was compared to all other cells via the Wilcoxon rank sum test for differential expression testing (min.pct = 0.25, logfc.threshold = 0.25). Marker gene expression was visualized using feature plots as well as expression heatmaps. For computing differentially expressed genes between (un)treated homo- versus heterotypic spheroid-derived tumor cells, differential expression testing (Wilcoxon rank sum test, min.pct = 0.25, logfc.threshold = 0.25) was performed on all cells while excluding the fibroblast subcluster.

### Gene set enrichment analysis (GSEA)

To investigate perturbed biological processes and pathways upon ALKi treatment and co-culture with activated fibroblasts, GSEA (Molecular Signatures Database (MSigDB); http://software.broadinstitute.org/gsea/msigdb; version 4.03.) was conducted on differentially expressed genes between datasets. For GSEA, log-transformed expression fold-change values of the analyzed genes that passed the following thresholds were used:|log(fold change)| > 0.25 and the gene was detected in > 25% of the cells in each of the compared groups. FDR correction was applied to the results. Enrichment analysis was performed using GSEAPreranked (desktop application v4.0.3) with permutations value set to 1000 and weighted enrichment statistics. The gene sets were pre-filtered according to gene set sizes, with a maximum of 500 and a minimum of 10 genes per gene set being allowed. The expression signatures for selected pathways (Hallmarks & Canonical pathways including REACTOME) were downloaded from MSigDB.

### Cell-cell interaction analysis

Physical and intercellular signaling interactions between lung cancer cells and fibroblasts were predicted by applying the R package ‘RNA-magnet’ (version 0.1.0). Prior to analysis, the highly curated list of ligand-receptor pairs (version 1.0.0) was prepared by changing from mouse to human annotations using the biomaRt package (version 2.42.1), and all entries were manually checked. Previously generated Seurat v3 objects were loaded and analyzed, including the permitted ligand localizations ‘Secreted’, ‘ECM’ and ‘Membrane’. Accordingly, cellular interactions were scored on the basis of receptors binding to other cell surface molecules, extracellular matrix components or secreted ligands.

### Lipogenic signature score expression analysis

The expression of the fatty acid gene set was analyzed as a lipogenic signature score using the ‘AddModuleScore’ function from the Seurat package. The gene set included the following fatty acid-related genes: *ACLY*,* ACACA*,* ACACB*,* FASN*,* SCD*,* FADS1*,* FADS2*,* FADS3*,* FADS6*,* ELOVL1*,* ELOVL3*,* ELOVL4*,* ELOVL5*,* ELOVL6*,* ELOVL7*, and *SREBF1*. Statistical significance was assessed using the Kruskal-Wallis test followed by Dunn’s post hoc test with Bonferroni correction. For the analysis of lipogenic signature scores in samples from TKI-naive and progressive disease patients, we utilized the scRNA-seq dataset from Maynard et al. [37]. The analysis was performed by comparing the expression of the lipogenic signature score between patient groups. Statistical significance was assessed using the Wilcoxon test rank-sum test.

### Lipidome profiling by liquid chromatography-mass spectroscopy (LC-MS)

For lipid extraction, pooled and (un)treated tumor spheroids were washed with 154 mM ice-cold ammonium acetate and snap frozen in liquid nitrogen. Spheroids were resuspended in 500 µl MeOH/H2O (80/20, v/v), the suspension was transferred to a glass tube, and another 500 µl MeOH/H2O (80/20, v/v) was added. Splash Lipidomix was added as an internal standard (10 µl per sample, Avanti Lipids). After the addition of 120 µl 0.2 M HCl, 360 µl chloroform, 400 µl chloroform and 400 µl of water with vigorous mixing between the pipetting steps, the samples were centrifuged at 3,000 × g for 10 min. 700 µl of the lower phase was collected and dried under a stream of nitrogen gas at 40 °C. Lipids were dissolved in 100 µl isopropanol prior LC-MS analysis. Lipids were separated on a C8 column (Accucore C8 column, 2.6 μm particle size, 50 × 2.1 mm, Thermo Fisher Scientific) mounted on a Ulitmate 3000 HPLC (Thermo Fisher Scientific) and heated to 40 °C. The mobile phase buffer A consisted of 0.1% formic acid in CH3CN/H2O (10/90, v/v), and buffer B consisted of 0.1% formic acid in CH3CN/IPOH/H2O (45/45/10, v/v/v). After injection of 3 µl lipid sample, 20% solvent B was maintained for 2 min, followed by a linear increase to 99.5% B within 5 min, which was maintained for 27 min. After returning to 20% B within 1 min, the column was re-equilibrated at 20% B for 5 min, resulting in a total run time of 40 min. The flow rate was maintained at 350 µl/min, and the eluent was directed to the ESI source of the QE Plus from 2 to 35 min. MS analysis was performed on a Q Exactive Plus mass spectrometer (Thermo Fisher Scientific) applying the following settings: *Scan settings*: Scan range – 200–1600 m/z in full MS mode with switching polarities (neg/pos) and data-dependent fragmentation; Resolution – 70,000, AGC target – 1E6; Max. injection time – 50 ms. *HESI source parameters*: Sheat gas – 30; Aux gas – 10; Sweep gas – 3; Spray voltage – 2.5 kV; Capillary temperature – 320 °C; S-lens RF level – 55.0; Aux gas heater temperature – 55 °C. *Fragmentation settings*: Resolution – 17,500; AGC target – 1E5; Max. injection time – 50 ms. Peaks corresponding to the calculated lipid masses (± 5 ppm) were integrated using El-Maven (https://resources.elucidata.io/elmaven).

The data was further analyzed using MetaboAnalyst 5.0 software (https://www.metaboanalyst.ca) [38]. PCA and unsupervised hierarchical clustering were performed to determine differences in the analyzed features. Saturation indices were calculated by summing peak intensities of phospholipids with the same level of (un)saturation, normalized to the total sum of peak intensities. Lorlatinib-induced alterations were analyzed as the log_2_ ratio of treated versus untreated samples.

### Statistical analysis

Statistical analysis was performed via GraphPad Prism 8. Quantitative data are presented as mean ± standard deviation (SD) and all statistical tests were performed of a minimum of three biological replicates. If not otherwise stated, statistical testing was conducted by a two-sided unpaired t test to determine significant differences between the control and corresponding treatment conditions. A p-value of < 0.05 was considered statistically significant (*, *p* <0.05; **, *p* < 0.01; ***, *p* < 0.001; ****, *p* < 0.0001).

## Results

### The presence of CAFs reduces apoptosis and promotes cell cycle progression of lung tumor cells upon ALK Inhibition

**Fig. 1:**
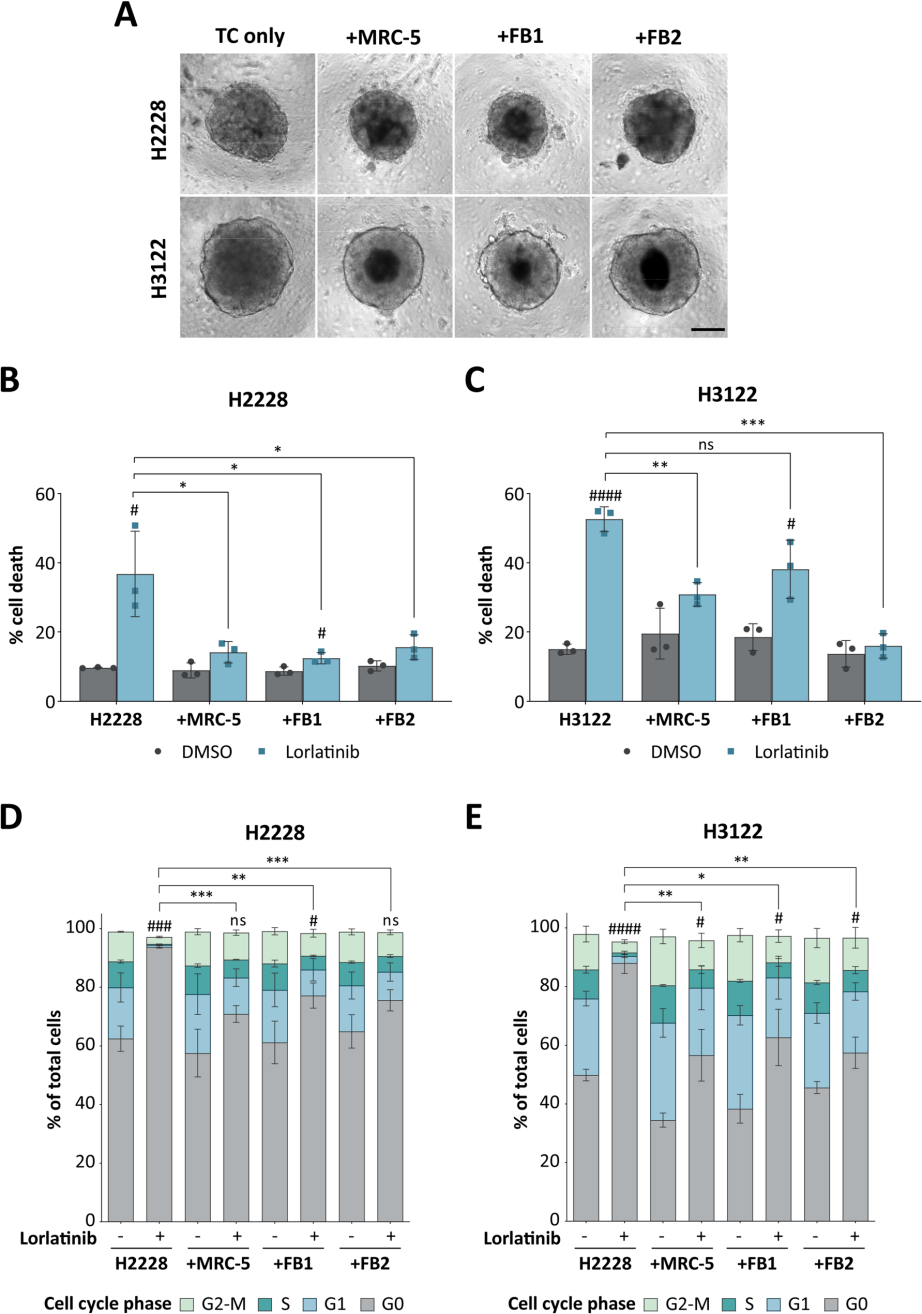
(**A**) H2228 or H3122 lung tumor cells (TCs) were seeded alone or in combination with three TGF-β1-activated fibroblast lines (MRC-5, FB1, FB2) for the generation of homo- and heterotypic lung tumor spheroids, respectively. Scale bar: 200 μm. Quantification of cell death rates of H2228 (**B**) and H3122 (**C**) cells as determined by the sum of early (Q3) and late apoptotic/necrotic (Q2) cells following flow cytometric analysis (*n* = 3). Data are presented as mean ± SD. #, *p* ≤ 0.05; ###, *p* ≤ 0.001 compared to the corresponding DMSO controls. ns, not significant; *, *p* ≤ 0.05; **, *p* ≤ 0.01 in comparison to lorlatinib-treated mono-cultures. FVD, fixable viability dye. Quantification of the portion of H2228 (**D**) and H3122 (**E**) cells according to their cell cycle phase status (*n* = 3). Data are presented as mean ± SD. ns, not significant; #, *p* ≤ 0.05; ###, *p* ≤ 0.001; ####, *p* ≤ 0.0001 for cells in the G0-phase compared to the corresponding DMSO controls. *, *p* ≤ 0.05; **, *p* ≤ 0.01; ***, *p* ≤ 0.001 for cells in the G0-phase in comparison to lorlatinib-treated mono-cultures. PI, propidium iodide

CAFs are known promoters of tumor cell resistance to systemic chemotherapy and targeted therapy interventions [8]. To investigate the stroma-mediated drug sensitivity of NSCLC tumor cells, we established 3D cultures of *EML4-ALK* translocation carrying H2228 (E6; A20) and H3122 (E13; A20) cells with and without CAFs (Fig. 1A). The CAF phenotype was induced via stimulation with 2 ng/ml TGF-β1 using MRC-5 fibroblasts and two primary fibroblast lines (FB1 and FB2) prior to co-cultivation. CAF activation was confirmed at the phenotypic and molecular level by a stellate cell-shaped morphology (Fig. S1A). Furthermore, elevated expression levels of α-smooth muscle actin (αSMA) and fibroblast activation protein (FAP) were also observed in fibroblasts treated with TGF-β1 in both 2D culture (Fig. S1B and C) and 3D culture (Fig. S1D). Analysis of dead and apoptotic tumor cells was then assessed by Annexin V+/FVD staining and subsequent flow cytometric analysis (Fig. S3A). Treatment with appropriate concentrations (IC_75_ values, Supplementary Table S1) of brigatinib and lorlatinib, as determined by dose-response modeling (Fig. S2), led to an increase in cell death in H2228 (Fig. 1B, Fig. S3B and C) and H3122 (Fig. 1C, Fig. S3D) spheroids. In contrast, direct co-cultivation with activated MRC-5, FB1, or FB2 cells significantly reduced tumor cell death upon TKI application compared to that of spheroids consisting of only tumor cells (Fig. 1B and C). Notably, the tumoricidal effect of both drugs appeared to be stronger in EML4-ALK variant 1 (v1)-driven H3122 cells than in EML4-ALK v3-driven H2228 cells (Fig. 1B and C, Fig. S3B and C). Next, combined staining of the proliferation-associated antigen Ki-67 and propidium iodide (PI) revealed significant changes in the cell cycle of homotypic H2228 (Fig. 1D, Fig. S4A and B) and H3122 (Fig. 1E, Fig. S4C) spheroids and a switch toward G0 arrest following brigatinib or lorlatinib treatment, respectively. In contrast, exposure of heterotypic H2228 and H3122 spheroids to both ALKis led to significantly increased cell cycle progression of the tumor cells in a CAF-dependent manner. Together, these results provide strong evidence that CAFs establish a protective niche for *EML4-ALK*-rearranged lung cancer cells toward ALK inhibition.

### Distinct clusters of ALK + NSCLC cells and CAFs as revealed by scRNA-seq

**Fig. 2:**
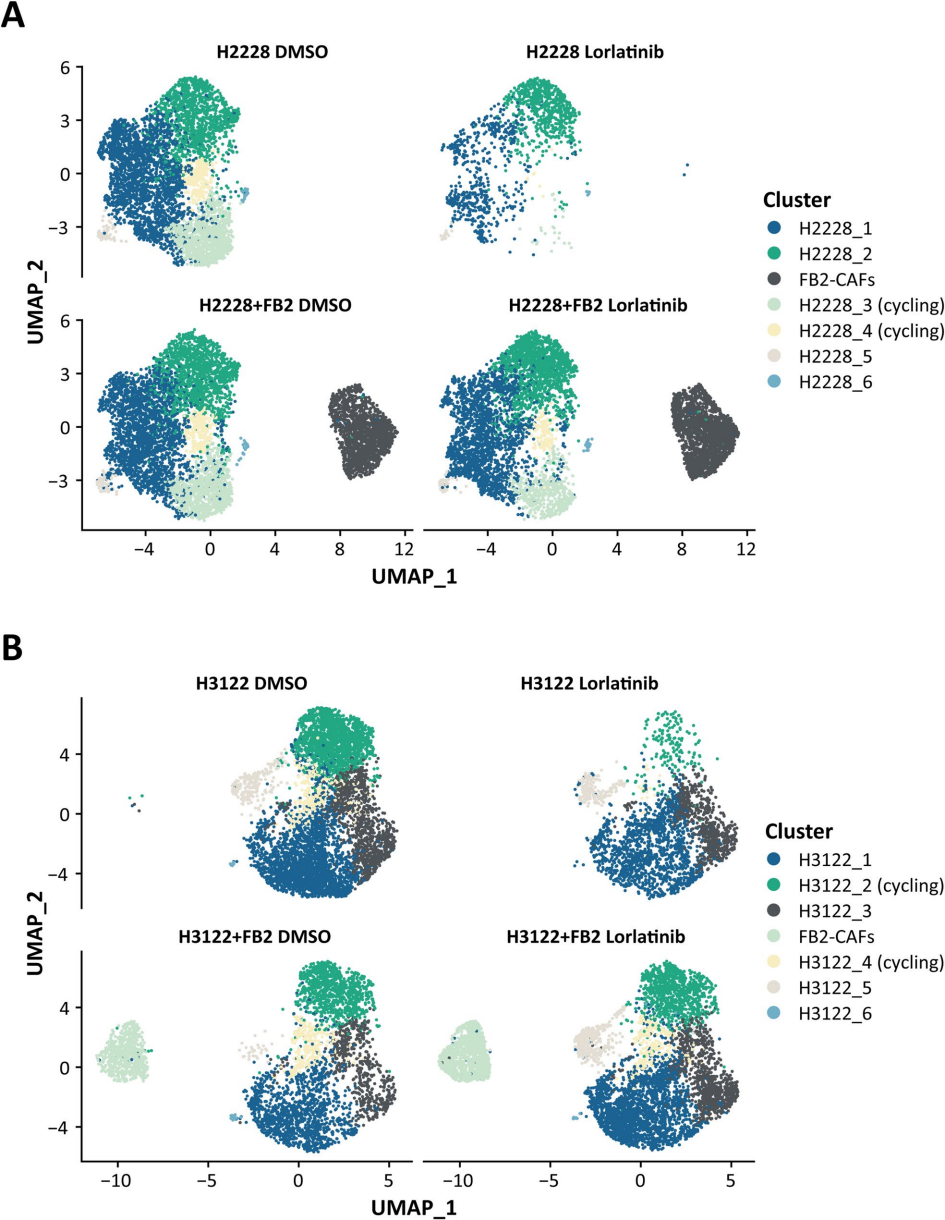
Uniform manifold approximation and projection (UMAP) representation of lorlatinib-treated H2228 (**A**) and H3122 (**B**) cells following graph-based clustering, colored by cluster identity

To analyze the molecular processes in tumor cells and CAFs, we applied scRNA-seq to dissociated H2228 and H3122 tumor spheroids either mono-cultured or co-cultured with TGFβ1-activated FB2 fibroblasts. We acquired transcriptome data for a total of 86,636 individual cells, with an average of 4,600 unique genes detected per cell (Supplementary Table S2). To determine differentially expressed genes (DEGs), we employed a series of analytical steps for each individual dataset, including quality control and filtering, principal component analysis (PCA) and a graph-based clustering approach. Dimensionality reduction and visualization of cell-type clusters defined by their transcriptional profiles were performed via uniform manifold approximation and projection (UMAP). Seven clusters (numbered 0–6) were observed for both lorlatinib-treated H2228 (Fig. 2**A**) and H3122 (Fig. 2B) spheroid-derived samples. To characterize inter- and intrapopulation heterogeneity and define the identity of the clusters, pairwise differential gene expression analysis for each cluster against all others was performed to generate cluster-specific marker genes conserved across all conditions. A total of 1,161 (H2228) and 1,000 (H3122) genes were identified that best grouped the cells into the seven subgroups. Heatmaps illustrating the ten most differentially expressed genes of each cell cluster are shown in Fig. S5. Known markers associated with matrix remodeling (e.g., *TIMP1*,* COL1A2*), fibroblast activation (e.g., *IGFBP5*,* FAP*), fibroblast-derived growth factors (e.g., *HGF*,* FGF2*) and cytokines (e.g., *CCL2*) clearly identified transcriptionally distinct cluster 2 (H2228, Fig. S5A) or cluster 3 (H3122, Fig. S5B) cells as the CAF population. This was supported by the fact that these clusters were present only in the co-culture samples (Fig. 2A and B). The H2228 and H3122 tumor cell fractions were divided into six subclusters on the basis of UMAP analysis. This heterogeneity could be partly explained by the presence of cycling and proliferating cells in cluster 3 and 4 within the H2228 sample group (Fig. S5A) as well as in cluster 1 and 4 of the H3122 samples (Fig. S5B). This finding was further supported by the specific expression of cell proliferation (e.g., *TOP2A*,* PCLAF*, and *CENPF*) and cell cycle (e.g., *PTTG1* and *CCNB1*) markers. The abundance of cells within these cycling clusters was also clearly lower in the lorlatinib-treated mono-culture-derived samples than in all the other samples (Supplementary Table S3), which corroborates the findings of the previous cell cycle analysis (see Fig. 1D and E).

### Co-culture with CAFs drives upregulation of the lipid metabolic pathway in ALKi-treated lung tumor spheroids

**Fig. 3:**
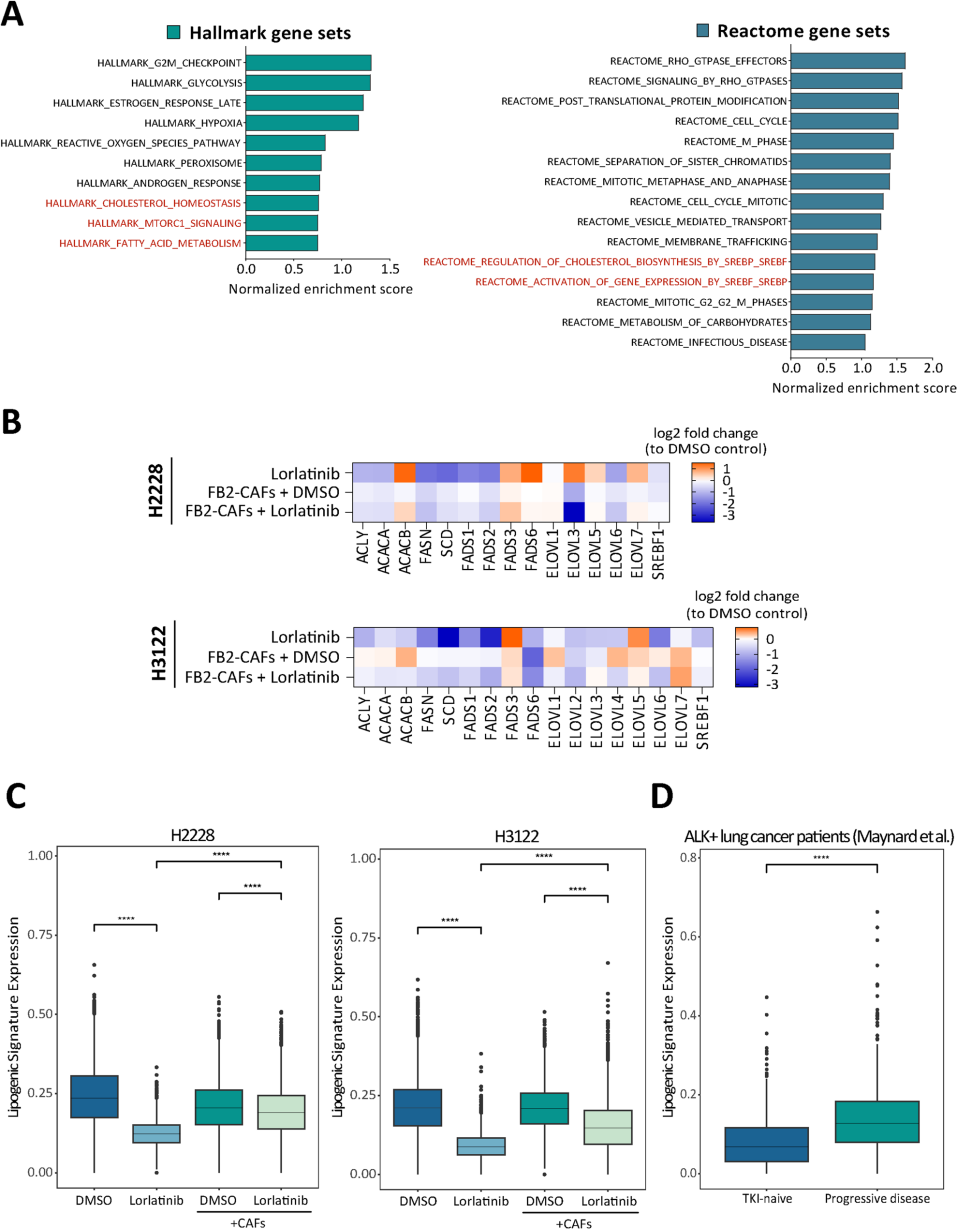
(**A**) Top deregulated pathways of hallmark (green bars) and reactome (blue bars) gene sets derived from the Molecular Signatures Database (MSigDB) in FB2 co-cultured versus mono-cultured H2228 and H3122 cells following ALK inhibition on the basis of gene set enrichment analysis (GSEA) (FDR ≤ 0.25). (**B**) Expression heatmap of fatty acid metabolism-related genes in single-cell transcriptome datasets of lorlatinib-treated H2228 and H3122 cells. (**C**) Boxplots showing the expression of lipogenic genes (as shown in **B**) in non- and lorlatinib-treated mono- vs. co-cultured H2228 and H3122 cells. (**D**) Boxplots showing the expression of lipogenic genes (as shown in B) in TKI-naїve vs. progressive disease ALK + lung cancer patients. Single-cell RNA sequencing data were derived from Maynard et al. [37]. All boxplots represent the interquartile range, with whiskers drawn to the highest value within the upper and lower fences (upper fence, 75th quantile + 1.5× interquartile range; lower fence, 25th quantile – 1.5× interquartile range). The solid middle line in the boxplot represents the median value. The gene expression data was analyzed using the Kruskal-Wallis test followed by Dunn’s post hoc test with Bonferroni correction. For comparisons based on patient-level data with only two groups, the Wilcoxon rank-sum test was applied; ****, *p* ≤ 0.0001

In order to explore the molecular processes facilitating the survival of ALK-driven lung cancer cells under the influence of CAFs, differential gene expression analysis between treated mono- and co-culture conditions was performed. After the exclusion of CAF clusters, 334 to 609 DEGs (avg. logFC|≥ 0.25|) were identified in brigatinib- or lorlatinib-treated H2228 and H3122 mono-cultures compared to those in co-culture conditions (Fig. S6A). Compared to those in mono-culture, considerable overlap of upregulated genes in TKI-treated lung tumor cells in response to CAF co-culture was observed (Fig. S6B, Fig. S7). These DEGs were subjected to gene set enrichment analysis (GSEA) via the Molecular Signatures Database (MSigDB), which revealed enriched pathways related to cell proliferation, metabolic activity, and signal transduction related to Rho-GTPases (Fig. 3A). Moreover, terms linked to lipid metabolism were among the top enriched biological processes and pathways. Since alterations in lipid metabolism are increasingly recognized to support tumorigenesis and therapy resistance [17, 39], we focused on this metabolic pathway for our further investigations. A selection of genes encoding lipogenic regulators are highlighted in Fig. 3B and C and Fig. S8, demonstrating the upregulation of *de novo* lipogenic genes in TKI-treated co-cultures when compared to the corresponding mono-culture conditions. Of note, the gene encoding stearoyl-CoA desaturase-1 (*SCD*), a key regulatory factor of fatty acid (FA) synthesis, was among the top 79 upregulated genes (Fig. S7). *SCD* is a well-established transcriptional target of sterol regulatory binding protein 1 (SREBP-1), which is encoded by the sterol regulatory element-binding transcription factor 1 (*SREBF1*) gene, whose downregulation following ALK-TKI treatment was also prevented by co-culture with CAFs. An examination of the average expression of a set of lipid-associated genes, represented by the fatty acid score, did not indicate any enrichment in a cluster-specific manner (Fig. S9). However, the depletion of these lipogenic genes upon lorlatinib treatment was consistently observed across all the tumor cell clusters. The differential expression of ATP citrate lyase (*ACLY*), acetyl-CoA carboxylase alpha (*ACACA*), fatty acid synthase (*FASN*), *SCD*, and *SREBF1* was further validated via RT‒qPCR subsequent to magnetic-based cell sorting of co-cultured H2228 and H3122 tumor spheroids (Fig. S10). To provide a more clinically relevant assessment, we utilized the dataset from Maynard et al. [37], which includes single-cell RNA-seq data from clinical biopsies of lung cancer patients, encompassing both treatment-naïve (TN) and progressive disease (PD) cases, including several ALK + samples. Our analysis revealed significantly greater expression of lipogenic genes in tumor cells from TNs than in those from PD patients (Fig. 3D). These findings suggest that the increase in fatty acid metabolism in TN patients could play a critical role in mediating resistance to ALK inhibition, potentially through a mechanism involving CAFs, as demonstrated by our in vitro data. These findings underscore the importance of targeting lipid metabolic pathways to overcome CAF-dependent resistance in ALK + lung cancer therapy.

### Ligand‒receptor interaction analysis predicts CAF-associated ligands that convey therapy resistance in ALK + lung cancer cells

**Fig. 4:**
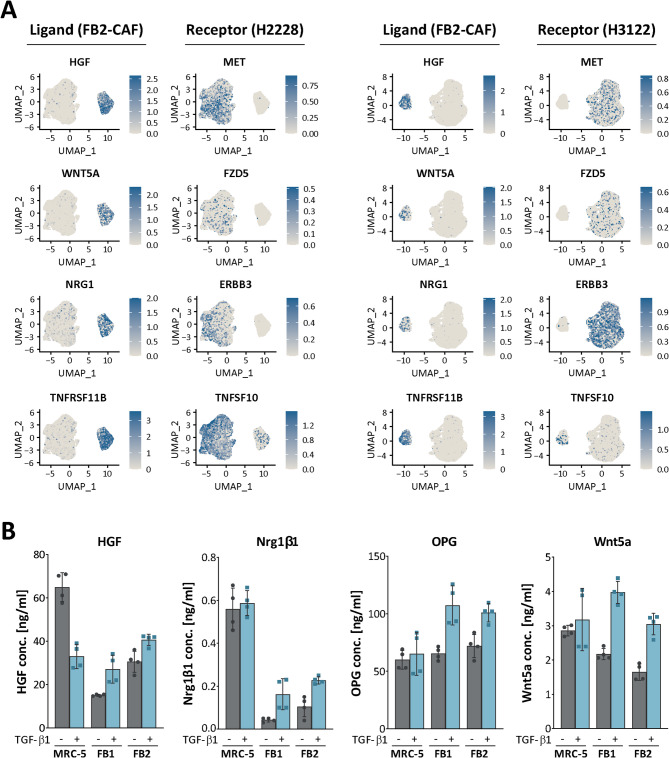
(A) Inference of cellular interactions from single-cell transcriptome data using ‘RNA-Magnet’. Routes of cell-cell communication between FB2-CAFs and H2228 or H3122 lung tumor cells were predicted following ligand-receptor interaction analysis. Selected ligand-receptor pairs are visualized as feature plots. (B) Validation of CAF-secreted candidate ligands using Luminex and ELISA. Concentrations were measured according to generated standard curves and are given for native versus TGF-β1-activated fibroblasts (*n* = 2). All data are presented as mean ± SD

To identify secreted factors and their cognate receptors for paracrine-mediated signaling, an in silico analysis of the scRNA-seq data was conducted by employing the ‘RNA-magnet’ algorithm [40]. Putative tumor–stroma interactions were inferred on the basis of a curated list of ligand‒receptor pairs and the enrichment of ligand‒receptor transcripts between the CAF population and each tumor cell cluster. The results of the top predicted interaction pairs (interaction score > 0.2) across all conditions for both the H2228 and H3122 samples are listed in Table 2. Among the computed interactions, key paracrine ligands derived from CAFs include RTK ligands such as HGF, FGF2 and NRG1 as well as Wnt signaling-associated factors (e.g., WNT5A and SFRP1). The highly abundant HGF was predicted to bind not only to its cognate receptor MET but also to SDC1 and ST14 (Table 2; Fig. 4A). HGF is a widely known CAF-secreted factor, and the HGF/MET-axis has often been found to be associated with cancer progression and the induction of therapy resistance [41, 42]. Secreted WNT5A was suggested to interact with a wide range of tumor cell-expressing receptors, such as LRP5, LRP6, FZD5, and FZD6 (Table 2; Fig. 4A). WNT5A, an activator of noncanonical Wnt pathways, has protumorigenic functions and contributes to inflammation and immunosuppression in the tumor microenvironment [43]. Another interesting computed stromal‒epithelial interaction pair was NRG1‒ERBB3 (Table 2; Fig. 4A). The CAF-derived factor NRG1 was demonstrated to modulate the therapeutic response in a variety of cancers [44, 45]. Notably, NRG1 signaling via ErbB receptors is involved in the (lipid-demanding) myelination by Schwann cells [44, 47] and oligodendrocytes [48]. In addition, interactions between the CAF-derived decoy receptor TNFRSF11B/OPG and the apoptosis-inducing ligand TNFSF10/TRAIL have been suggested. Interestingly, OPG was previously reported to protect cancer cells from TRAIL-induced apoptosis [49, 50].

**Table 2:** Predicted interaction pairs obtained via ligand-receptor interaction analysis using ‘RNA-magnet’

Predicted paracrine interaction pairs		
Score	Ligand	Receptor
0.707	HGF	SDC1
0.627	WNT5A	LRP5
0.593	HGF	MET
0.593	IGFBP4	LRP6
0.580	HGF	ST14
0.478	WNT5A	RYK
0.473	WNT5A	LRP6
0.452	TNFRSF11B	TNFSF10
0.435	SFRP1	FZD6
0.426	FGF2	SDC1
0.363	WNT5A	FZD5
0.316	WNT5A	FZD6
0.311	NRG1	ERBB3
0.279	BDNF	SORT1
0.273	FGF2	SDC4

Ligand‒receptor interaction analysis revealed a variety of CAF-secreted factors that could act as potential communicators between CAFs and lung tumor cells and thus as initiators of stroma-driven therapy resistance. To corroborate these findings, spheroid growth analyses via combination treatment with ALK-TKIs and CAF-conditioned medium (CM) derived from TGF-β1-activated and 3D-cultured lung FB2 fibroblasts demonstrated increased growth of H2228 (Fig. S11A and B) and H3122 (Fig. S11A and C) spheroids as opposed to treatment with brigatinib or lorlatinib alone. These experiments indicated that CAF supernatants contain molecular factors that promote the treatment resistance of tumor cells.

To validate the expression of selected CAF-associated ligands inferred via interaction analysis, the active secretion of HGF, NRG1β1, TNFRSF11B/OPG, and WNT5A in cell culture supernatants derived from previously 3D-cultured MRC-5, FB1 and FB2 fibroblasts was measured via multianalyte profiling. All tested ligands were detected in conditioned media obtained from both untreated and TGF-β1-treated fibroblasts (Fig. 4B). Here, differences in analyte concentrations were observed in a fibroblast line- and activation status-dependent manner. Notably, the *NRG1* gene gives rise to numerous protein isoforms (e.g., NRG1α and NRG1β1) through alternative promoter usage and splicing [51]. The total NRG1 concentration is therefore likely higher in fibroblast CM than the detected 0.02–0.7 ng/ml NRG1β1 alone. Interestingly, the secretion levels of all analytes tested increased following TGF-β1-mediated activation in MRC-5, FB1 and FB2 fibroblasts. In contrast, activated MRC-5 fibroblasts secreted less HGF than their native counterpart, a finding that is consistent with previous studies, which demonstrate a TGF-β1-induced degradation of full-length HGF mRNA in MRC-5 cells due to post-transcriptional regulation [52, 53].

**Fig. 5:**
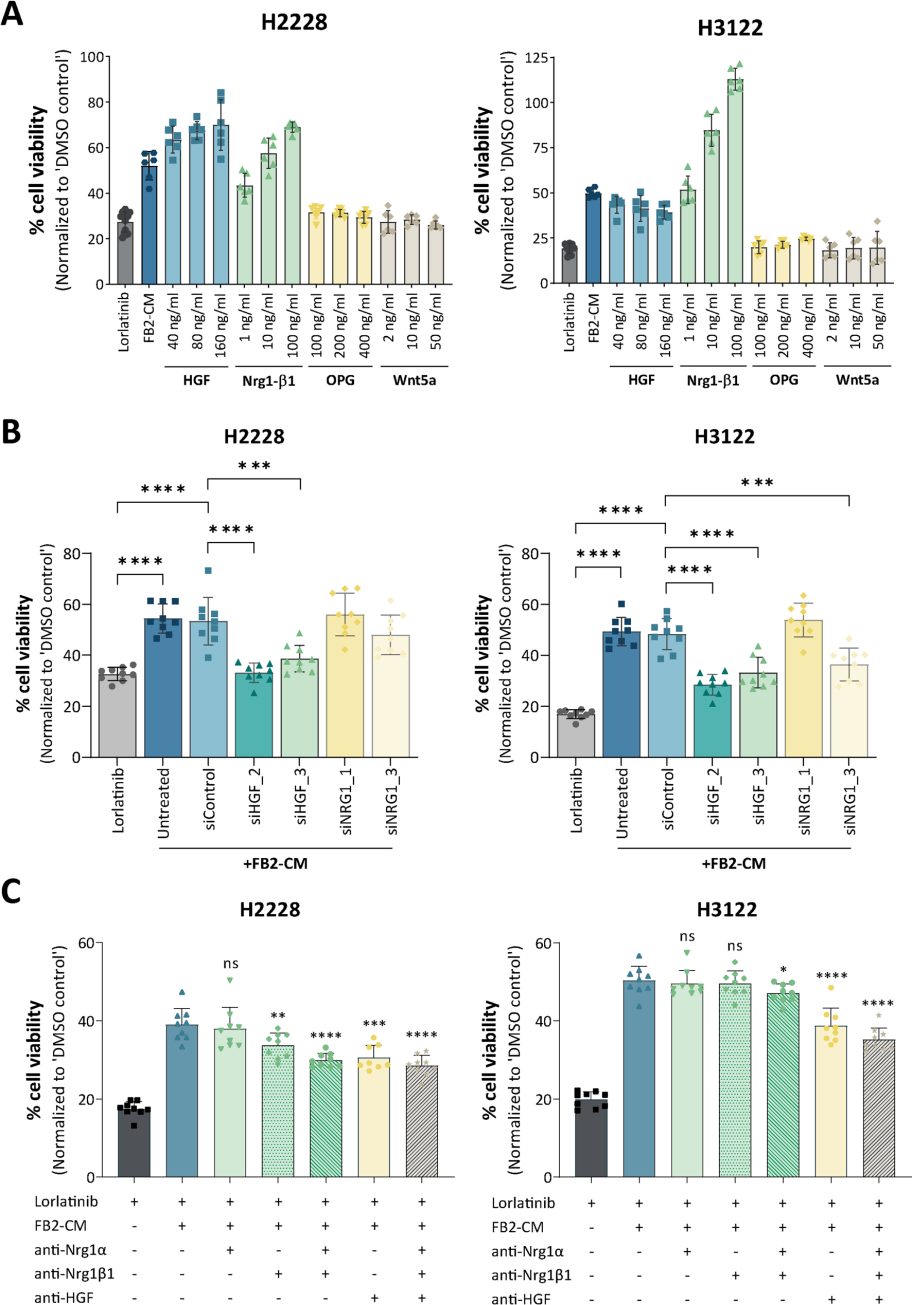
(**A**) Cell viability analysis of ALK-inhibited lung tumor cells following treatment with CAF-associated ligands (*n* = 2). (**B**) Cell viability analysis of lorlatinib-treated H2228 and H3122 lung tumor spheroids following treatment with CM derived from FB2 fibroblasts transfected with siRNA targeting HGF (siHGF_2, siHGF_3) or NRG1 (siNRG1_1, siNRG1_3) compared to untreated FB2-CM (*n* = 3). (**C**) Assessment of cell viability of lorlatinib-treated lung tumor spheroids subsequent to addition of FB2-conditioned medium (CM) and inhibitory antibodies directed against the CAF-associated ligands Nrg1α, Nrg1β1, and HGF (*n* = 3). All data are presented as mean ± SD. ns, not significant; *, *p* ≤ 0.05; **, *p* ≤ 0.01; ***, *p* ≤ 0.001; ****, *p* ≤ 0.0001 in comparison to treatment with lorlatinib and FB2-CM. HGF, hepatocyte growth factor; NRG1, neuregulin 1; TGF-β1, transforming growth factor-beta 1; OPG, osteoprotegerin

To address the possibility that the selected ligands alone could have mechanistic consequences on lung tumor cell drug sensitivity, recombinant HGF, NRG1β1, OPG, and WNT5A proteins were applied to ALKi-treated H2228 and H3122 tumor spheroids. Lorlatinib treatment considerably reduced cell viability compared to the vehicle control (Fig. 5A). OPG and WNT5A appeared to possess no paracrine potency in mediating tumor cell drug sensitivity under any of the tested conditions. Since these ligands are secreted by all fibroblast lines, a pivotal role for profibrotic autocrine signaling cannot be excluded. The addition of HGF or NRG1β1, however, could partially or fully reverse the TKI-induced decrease in H2228 and H3122 cell viability. This effect was observed in a concentration-dependent manner for NRG1β1, whereas increasing HGF concentrations did not result in a cumulative tolerability of H2228 and H3122 spheroids toward ALK inhibition. To further validate the contribution of fibroblast-derived HGF and NRG1 to tumor cell resistance, siRNA-mediated knockdown experiments were performed in FB2 fibroblasts. Efficient target gene knockdown by siHGF and siNRG1 was validated by qPCR (Fig. S12). Treatment with FB2-CM derived from fibroblasts transfected with siHGF_2 or siHGF_3 led to a significant reduction in cell viability compared to untreated FB2-CM in both H2228 and H3122 spheroids, confirming the critical role of HGF in mediating resistance (Fig. 5B). In contrast, knockdown with siNRG1_1 did not affect spheroid viability in either H2228 or H3122 cells. Interestingly, siNRG1_3 showed a modest but non-significant trend toward reduced viability in H2228, while significantly decreasing viability in H3122 spheroids. In line with these findings, the resistance-mediating effect of FB2-CM addition to lorlatinib-treated H2228 and H3122 tumor spheroids was partially inhibited by neutralizing antibodies directed against HGF, NRG1α, and NRG1β1 as well as combinations thereof (Fig. 5C). Taken together, these data establish a direct correlation between CAF-associated paracrine factors and therapy resistance in H2228 and H3122 cancer cells.

### The CAF-derived secretome upregulates lipid metabolism and AKT signaling in ALKi-treated lung tumor spheroids

**Fig. 6:**
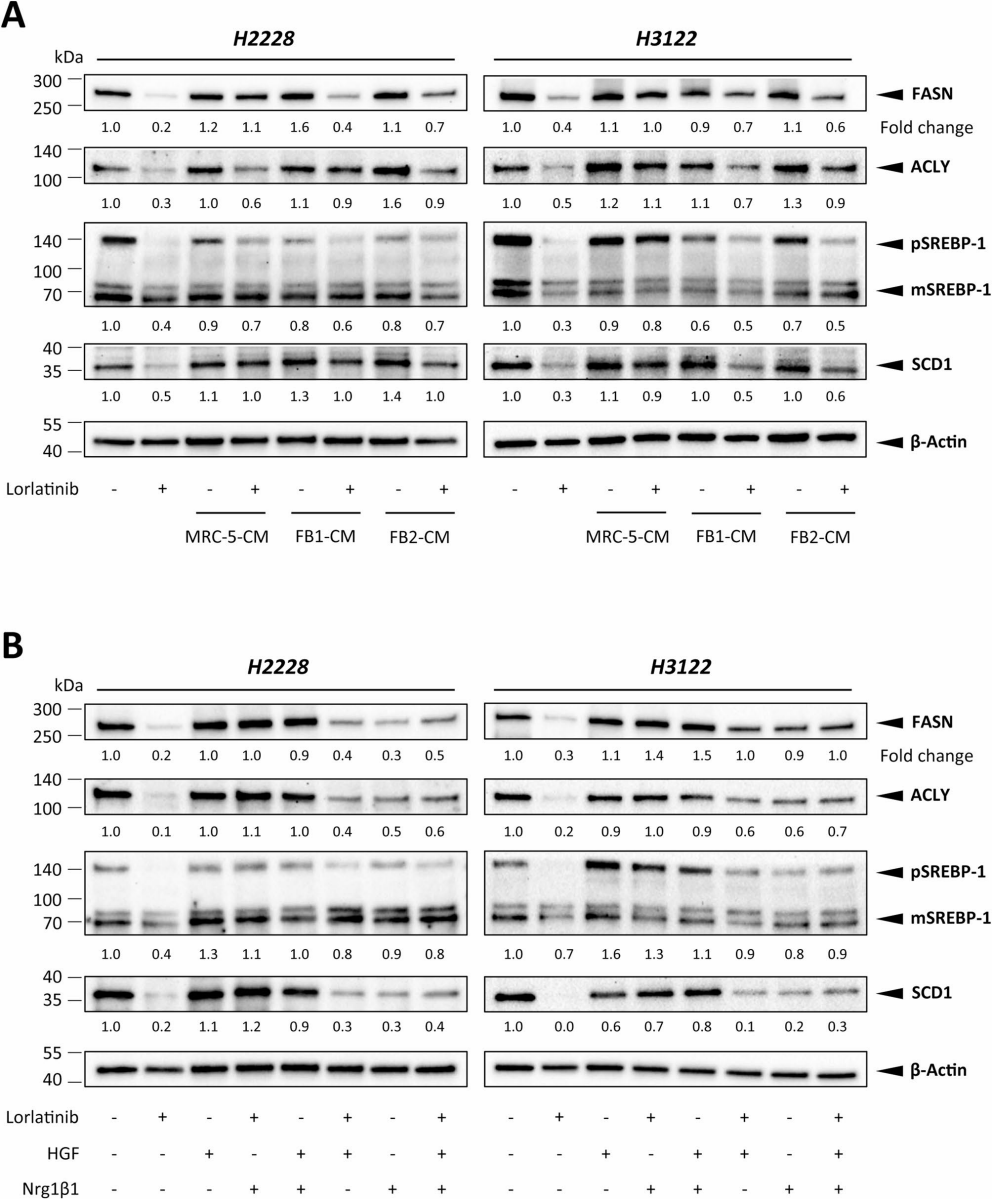
FB2-conditioned medium (CM) (**A**) and CAF-associated ligands HGF and Nrg1β1 (**B**) influence the expression of fatty acid metabolism-regulating targets upon ALK signaling perturbation via lorlatinib in H2228 and H3122 cells, as shown by representative western blots of FASN, ACLY, and SREBP-1 in H2228 and H3122 cells. Expression fold changes were normalized to those of β-actin. ACLY, ATP-citrate lyase; FASN, fatty acid synthase; pSREBP-1, precursor sterol-responsive element binding protein-1; mSREBP-1, mature SREBP-1

The differential expression of lipogenic enzymes upon ALK inhibition could be validated under direct co-culture conditions. Since the CAF-derived secretome was also able to interfere with drug susceptibility in ALK-driven lung tumor spheroids, we hypothesized that a potential link exists between CAF-mediated paracrine signaling and enhanced lipid metabolic activity in tumor cells. Western blot analysis of selected lipogenic enzymes suggested that Lorlatinib caused a decrease in ACLY and FASN concomitant with reduced expression of their upstream transcription factor SREBP-1. The addition of CAF-CM derived from MRC-5, FB1, and FB2 fibroblasts (Fig. 6A, Additional File 1), as well as the single CAF-associated ligands HGF and NRG1β1 (Fig. 6B, Additional File 2), was able to partially restore the observed protein expression changes, respectively. A combination of both ligands, however, was not able to fully restore the expression of lipogenic enzymes.

**Fig. 7:**
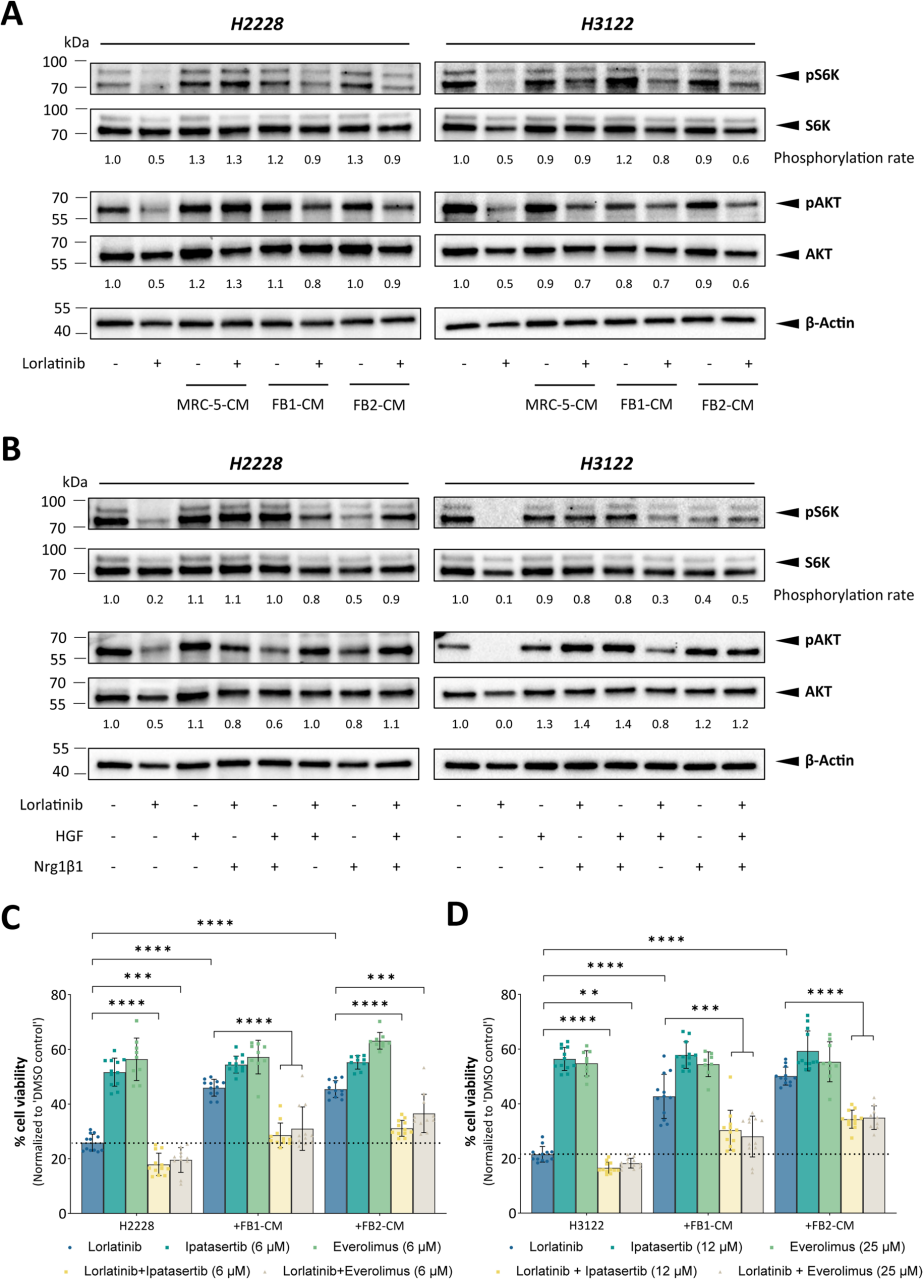
FB2-conditioned medium (CM) (**A**) and CAF-associated ligands HGF and Nrg1β1 (**B**) influence the activation of AKT signaling upon ALK signaling perturbation via lorlatinib in H2228 and H3122 cells, as shown by representative western blots of AKT/pAKT and S6K/pS6K in H2228 and H3122 cells. Phosphorylation rates were normalized to those of β-actin. AKT, protein kinase **B**; S6K, ribosomal S6 kinase

The PI3K-AKT pathway is a commonly activated signaling pathway downstream of mutant ALK [54]. Previous work demonstrated the induction of lipid biosynthesis via PI3K-AKT-mTOR signaling, with mTORC1 being a direct regulator of SREBP transcription factor activity [55, 56]. We therefore investigated whether the fibroblast-derived secretome enhances AKT signaling in ALK-inhibited lung tumor spheroids. While ALK inhibitor treatment led to a reduction in phosphorylated AKT and the mTOR downstream target S6K1, AKT signaling was effectively reinduced following the cultivation of H2228 and H3122 tumor spheroids with CAF-CM (Fig. 7A, Additional File 3). Treatment with exogenous HGF or NRG1β1 was also sufficient to induce the phosphorylation of AKT and S6K1 despite the inhibition of oncogenic ALK (Fig. 7B, Additional File 4), whereas co-treatment with both ligands was not able to fully re-establish the phosphorylation levels of AKT and S6K1. These data imply that the CAF-derived secretome confers therapeutic resistance via induction of the PI3K–AKT–mTOR signaling axis and thereby drives the expression of SREBP targets. To further strengthen our conclusion, we tested the effects of ipatasertib, a highly selective pan-AKT inhibitor, and everolimus, a widely used mTORC1/2 inhibitor, on the ability of CAF-CM to rescue ALK inhibition. Dual inhibition with lorlatinib and ipatasertib or everolimus significantly reduced the cell viability of H2228 (Fig. 7C) and H3122 (Fig. 7D) lung tumor spheroids, demonstrating the potential of AKT/mTOR signaling inhibition to overcome the resistance induced by the CAF secretome.

### CAF-CM rescues lung tumor spheroids from TKI-induced interference with de Novo lipogenesis and lipid peroxidation

**Fig. 8:**
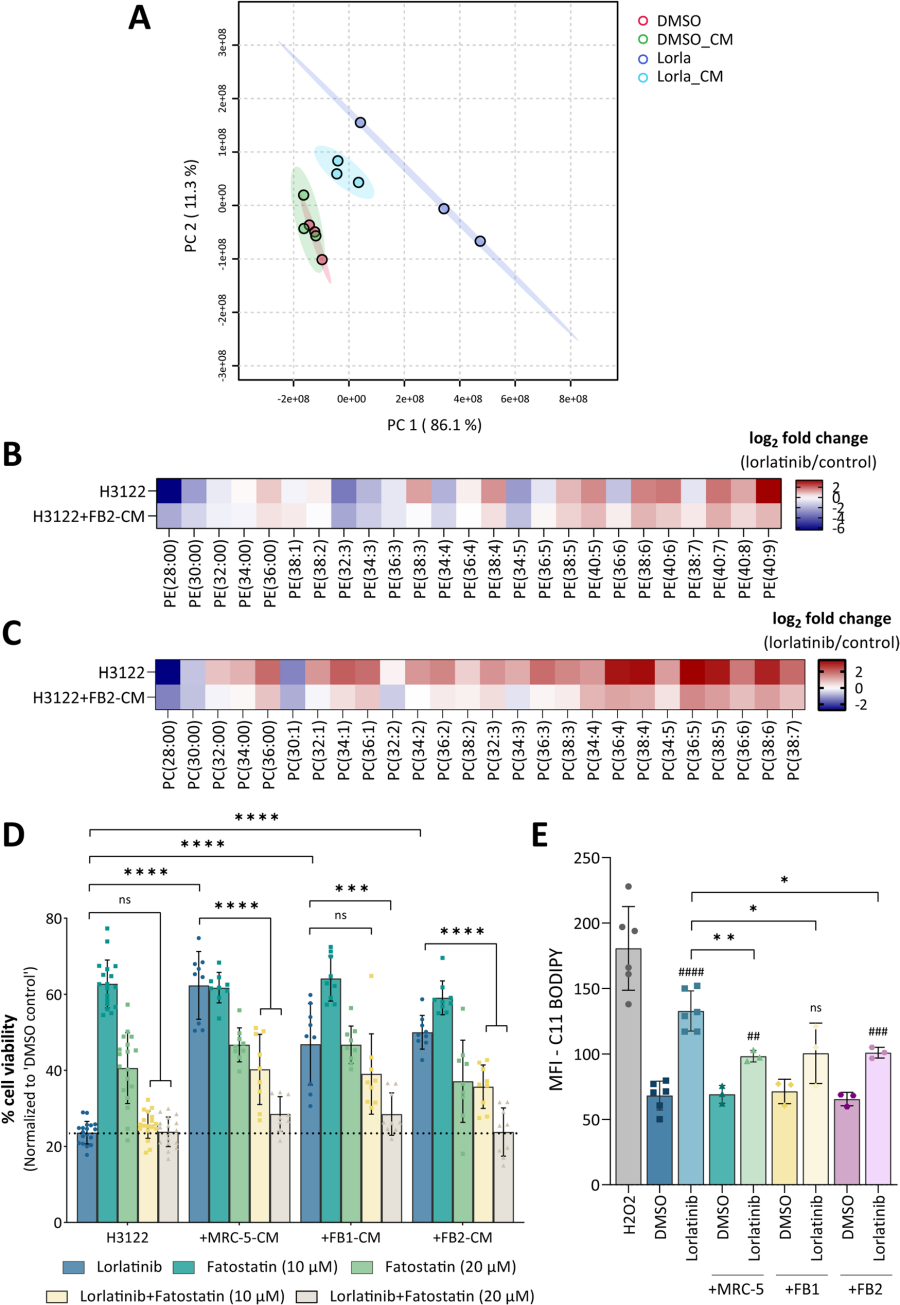
(**A**) Principal component analysis (PCA) of lipidomic profiles of lorlatinib-treated H3122 cells with and without FB2-conditioned medium. (**B**) Heatmap of log_2_ ratios of phosphatidylethanolamine (PE) species in lorlatinib-treated H3122 cells with and without FB2-conditioned medium compared to vehicle-treated H3122 cells. PE species are indicated by their total number of fatty acid carbons, followed by the total number of unsaturations (*n* = 3). (**C**) Heatmap of log_2_ ratios of phosphatidylcholine (PC) species in lorlatinib-treated H3122 cells with and without FB2-conditioned medium compared to vehicle-treated H3122 cells. PC species are indicated by their total number of fatty acid carbons, followed by the total number of unsaturations (*n* = 3). (**D**) Cell viability analysis following combination treatment of H3122 tumor spheroids with fatostatin, lorlatinib, and fibroblast CM (*n* = 3). (**E**) Quantification of lipid peroxidation in H3122 spheroids using C11 BODIPY. Hydrogen peroxide (H_2_O_2_) served as a positive control. All data are presented as mean ± SD. ##, *p* ≤ 0.01; ###, *p* ≤ 0.001; ####, *p* ≤ 0.0001 compared to the corresponding DMSO controls. ns, not significant; *, *p* ≤ 0.05; **, *p* ≤ 0.01; ***, *p* ≤ 0.001; ****, *p* ≤ 0.0001 in comparison to lorlatinib treatment alone

Since ALK inhibition evokes differential expression of lipid-metabolizing enzymes, we performed mass spectrometry-based phospholipidome profiling to evaluate changes in the proportions of lipid species. Inherent differences in the lipidomic profiles were visualized via principal component analysis (PCA; Fig. 8A) and unsupervised hierarchical clustering (Fig. S13), which clearly distinguished between untreated and lorlatinib-treated samples. Inhibition of oncogenic ALK leads to an increase in polyunsaturated fatty acids (PUFAs) of phosphatidylethanolamine (PE, Fig. 8B) and phosphatidylcholine (PC, Fig. 8C) phospholipid species at the expense of saturated and monounsaturated phospholipids, a typical observation following the inhibition of *de novo* lipogenesis [57]. The addition of CAF-CM to H3122 lung tumor spheroids partly abrogated this shift toward higher levels of polyunsaturated lipids, a finding that was further corroborated by calculating saturation indices of all phospholipid species (Fig. S14A).

To further determine the importance of lipogenesis in the cell proliferative response of TKI-resistant ALK + lung cancer cells, we evaluated the effect of fatostatin, a small molecule inhibitor that hinders the trafficking of SREBP-1 to the Golgi compartment and thereby its proteolytic cleavage and activation. Viability assays revealed that SREBP-1 inhibition through fatostatin sensitized lung tumor spheroids to ALK inhibition despite the presence of CAF-CM (Fig. 8D, Fig. S14B).

The above findings suggest that CAF-driven therapy resistance is a result of increased lipid saturation and thus decreased lipid peroxidation, since PUFAs are more prone to lipid peroxidation than are saturated phospholipids [57]. To test this hypothesis, we measured lipid reactive oxygen species (ROS) accumulation via BODIPY 581/591 C11 fluorescence staining. Flow cytometry analysis revealed a strong increase in lipid peroxidation subsequent to lorlatinib treatment in both H2228 (Fig. S14C) and H3122 spheroids (Fig. 8E). In contrast, tumor spheroids treated with CAF-CM accumulated fewer lipid peroxides despite the inhibition of ALK.

To functionally assess whether promoting lipid peroxidation could overcome CAF-mediated resistance, we performed co-treatment experiments with the ferroptosis inducers Erastin and RSL3. Both compounds were able to sensitize tumor spheroids to lorlatinib in the presence of CAF-CM (Fig. S14D and E). Notably, RSL3 exhibited higher potency compared to Erastin, consistent with its direct inhibition of glutathione peroxidase 4 (GPX4), a key enzyme responsible for detoxifying lipid peroxides. In contrast, Erastin acts upstream by blocking cystine import via the cystine/glutamate antiporter (system xc−), resulting in glutathione depletion [58].

Collectively, these data show that interference with ALK signaling inhibits *de novo* lipogenesis concomitant with an increase in lipid peroxidation, an effect that is attenuated in the presence of CAF-CM. Furthermore, sensitization of resistant tumor spheroids to ALK inhibition can be achieved not only through metabolic reprogramming via SREBP-1 inhibition but also by direct pharmacological induction of ferroptosis. These findings highlight ferroptosis as a promising vulnerability in stroma-mediated therapy resistance in ALK⁺ lung cancer.

## Discussion

Although precision oncology has made significant strides in tailoring therapy to the molecular alterations of individual tumors, acquired resistance to targeted drugs remains a key clinical challenge [3]. One opportunity for cancer cells to bypass the drug response is through cellular crosstalk with frequently abundant CAF populations. Thus, exploiting vulnerabilities occurring in the presence of CAFs represents an attractive strategy to overcome therapy resistance. To increase the pathophysiological importance of in vitro modeling, the co-culture model devised in this study involved the cultivation of EML4-ALK-driven lung adenocarcinoma cell lines and CAFs in a 3D setting. The generated lung tumor spheroids were utilized to explore signaling networks between the two heterologous cell types that shape susceptibility to molecular-directed drugs via microenvironmental cues. In kinase-addicted NSCLC, a reduction in tumor cell survival following EGFR- and ALK-directed therapy interventions could be counteracted by exposure to nonmalignant CAF populations or CAF-associated paracrine mediators [58–62]. This well-documented ability of CAFs to promote therapy resistance also became apparent following co-cultivation experiments with H2228 and H3122 lung tumor spheroids, as shown by the compromised ability of ALKi treatment to decrease cell proliferation and induce apoptosis. Interestingly, the survival of EML4-ALK v3-driven H2228 cells was consistently better than that of EML4-ALK v1-driven H3122 cells before and after the addition of fibroblasts, which is consistent with the worse outcomes observed for v3-driven ALK + NSCLC in clinical practice [63].

In addition to its unprecedented resolution of cellular heterogeneity, scRNA-seq provides a basis for disentangling individual cells in response to stimuli and in the context of their microenvironment [64, 65]. In the present study, transcriptionally distinct tumor cell and fibroblast clusters of heterotypic lung tumor spheroids were identified following nonlinear dimensionality reduction using UMAP and cluster marker analysis. The heterogeneity of lung tumor cells could be partially explained by clusters of cycling cells, underlined by congruent expression signatures of identified cluster markers with gene sets utilized for cell cycle annotation (e.g., *TOP2A*, *MKI67*, *PTTG1*, and *CENPF*). The fibroblast population expressed genes related to ECM remodeling, collagen formation, cell adhesion, chemotaxis and other secretory functions, which is consistent with previous reports that focused on characterization of the tumor microenvironment [66–69]. The heterogeneity of CAFs is largely context dependent, and increasing evidence points toward functional specializations among CAFs [70]. This is highlighted by the identification of different fibroblast populations, such as myofibroblastic CAFs (myCAFs) or inflammatory CAFs (iCAFs) [67, 69]. Although such stratifications into CAF subtypes could not be delineated herein, a rather mixed phenotype based on partly overlapping gene signatures among different CAF subtypes seemed likely. This finding was suggested to be driven by microenvironmental differences between in vitro and in vivo conditions (e.g., lack of an immune compartment or a lymphatic/vascular system), which can substantially influence fibroblast fate [70, 71]. Nevertheless, this hypothesis warrants further investigations in the present context.

In oncogene-addicted NSCLC, CAF-derived soluble factors confer treatment resistance through binding to their receptors on the tumor cell surface and activating downstream signaling pathways [61, 72]. Since the ability of CAF-CM to promote the growth of ALK-inhibited tumor spheroids implied a significant contribution of CAF-derived soluble factors to tumor cell resistance, further analyses focused on investigating paracrine signaling pathways as a route of intercellular communication. Among these, HGF represents a CAF-secreted ligand known to induce resistance toward EGFR- and ALK-directed TKIs via stimulation of its cognate receptor MET and downstream PI3K/AKT and MAPK signaling [59, 60]. Similarly, a direct link between CAF-derived NRG1 and the promotion of therapy resistance via ErbB3 receptor signaling was observed in *BRAF*-mutant melanoma [44] and prostate cancer [45]. NRG1 is also able to convey therapy insensitivity in NSCLC cells, e.g., through autocrine mechanisms or external stimuli [72–76]. In agreement with these observations, the resistance-inducing effect of the CAF-associated ligands HGF and NRG1 on TKI-treated H2228 and H3122 tumor spheroids was confirmed by increased cell viability despite ALK inhibition. In contrast, the resistance-inducing effects of CAF-CM could be attenuated following the addition of inhibitory antibodies directed against HGF and NRG1.

By analyzing scRNA-seq data, we identified the main molecular drivers of CAF-mediated therapy resistance and uncovered the association of enhanced lipogenic gene expression with a CAF-driven resistant phenotype in ALKi-treated lung adenocarcinoma cells. The activation of *de novo* lipid synthesis is frequently observed in cancer and is assumed to fuel high demands in membrane biogenesis during cell division and tumor growth in a cell-autonomous manner [17]. Accumulating evidence also suggests that multiple oncogenic signaling pathways converge on lipid synthesis, thereby creating metabolic dependencies of molecularly altered cancers [30, 31, 56, 77]. In addition, the induction of SREBP-1-driven *de novo* synthesis of endogenous lipids has become increasingly apparent in many drug-resistant cancer cells [39], and sustained lipogenesis has been shown to be a key mediator of acquired resistance toward molecularly directed drugs in oncogene-driven melanoma [32] and NSCLC [33, 78]. Notably, such reprogramming of lipid metabolism is influenced not only by cell-autonomous genomic alterations but also by nongenomic components and non-cell autonomous players, i.e., the tumor microenvironment [79]. However, the direct impact of CAFs on the induction of lipid pathway activity in therapy-resistant cancer cells, as presented in this project, remains largely unexplored.

Our results further revealed that ALK inhibition reduced the levels of saturated and monounsaturated phospholipids while increasing lipid polyunsaturation and lipid peroxidation. Since bis-allylic carbons are particularly reactive, saturated, monounsaturated, and polyunsaturated, acyl chains differ in their susceptibility to peroxidation by free radicals [57, 80]. Accordingly, the increase in lipid ROS following ALK-TKI treatment aligns with the high unsaturation levels of membrane lipid species. In contrast, the presence of CAF-CM largely reversed the lipid composition, rendering ALKi-treated lung tumor spheroids less susceptible to lipid peroxidation by limiting the degree of phospholipid polyunsaturation. Another study similarly demonstrated that CAFs were able to ameliorate lipid peroxidation product accumulation, accompanied by the induction of chemoresistance in gastric cancer cells [81]. In recent years, mounting evidence has linked oncogenic RTK activation, downstream signal transduction and metabolic reprogramming in neoplastic cells [82, 83]. Previous work has demonstrated that SREBP-1 activation is modulated via KRAS-driven MAPK activation [56], direct ERK1/2-specific SREBP phosphorylation [84], or mutant *BRAF* [32]. Nevertheless, most studies pinpoint to a signal transduction via the PI3K/AKT/mTORC1/SREBP axis downstream of active RTKs to induce *de novo* lipid biosynthesis [25, 28]. Our work likewise demonstrated that CAF-CM and the CAF-secreted factors HGF and NRG1 reinduced AKT signaling in ALKi-treated lung tumor spheroids, concomitant with the induction of SREBP-1 and its lipogenic targets. While the in-depth roles of CAF-secreted RTK ligands in regulating tumor cell lipid homeostasis have still not been sufficiently explored, some concordant indications can be derived from other studies. As such, HGF increases ACSVL3 expression levels in human glioma cells (U373), an enzyme that plays a central role in activating FAs for complex lipid synthesis [85]. NRG1 is currently heavily explored for its metabolic role in a variety of tissues [29] and has been shown to regulate cholesterol and fatty acid synthesis in the process of Schwann cell myelination [86, 87]. Critically, pharmacological targeting of SREBP-1 restored the sensitivity of resistant lung tumor spheroids to lorlatinib and was able to overcome the established CAF-mediated lipid metabolism-supportive niche, highlighting potential new therapeutic options for the clinical treatment of advanced NSCLC. In addition, co-treatment with ferroptosis inducers further supported the concept that CAF-mediated resistance involves protection from lipid peroxidation. Specifically, both Erastin and RSL3 were able to resensitize CAF-CM-treated tumor spheroids to ALK inhibition. The observed superior activity of RSL3 suggests that direct GPX4 inhibition may more effectively bypass compensatory antioxidant mechanisms induced by the tumor microenvironment. Our findings are consistent with previous studies showing that GPX4 inhibition is especially effective in eliminating therapy-resistant or metabolically adaptive cancer cells [88, 89]. These results highlight the therapeutic potential of targeting ferroptosis, especially through direct GPX4 inhibition, to overcome stromal-mediated resistance to ALK-targeted therapies.

Importantly, our findings suggest that these mechanisms are common for both standard (v1) and high-risk (v3) EML4-ALK fusion variants and could therefore provide novel strategies to counter the currently unmet need for ALK + disease with adverse molecular features [90].

## Conclusions

Overall, the systematic approach of combining advanced 3D cell cultures of *ALK*-translocated NSCLC cell lines and CAFs with scRNA-seq provides insights into distinct cell (sub)population-specific gene expression variabilities and in response to external stimuli. Our data further established a previously unrecognized role of CAFs in promoting resistance to ALK-TKI treatment through lipid metabolic reprogramming. These findings underscore the critical contribution of the tumor microenvironment to therapy resistance and highlight metabolic vulnerabilities, such as SREBP-1 activation and ferroptosis evasion, as promising targets to overcome CAF-mediated protection. Targeting these non-genetic resistance mechanisms may offer new therapeutic strategies to enhance the efficacy of ALK-targeted therapies in patients with ALK⁺ NSCLC.

## Electronic supplementary material

Below is the link to the electronic supplementary material.

Supplementary Material 1 Additional File 1: Raw unedited blots with highlighted bands of the western blots shown in panel (A) of Fig. 6.

Supplementary Material 2 Additional File 2: Raw unedited blots with highlighted bands of the western blots shown in panel (B) of Fig. 6.

Supplementary Material 3 Additional File 2: Raw unedited blots with highlighted bands of the western blots shown in panel (B) of Fig. 6.

Supplementary Material 4 Additional File 4: Raw unedited blots with highlighted bands of the western blots shown in panel (B) of Fig. 7.

Supplementary Material 5 Fig. S1: (A) Phase-contrast images of untreated (top) versus transforming growth factor-beta 1 (TGF-β1)-stimulated (bottom) MRC-5 fibroblasts and two primary fibroblast lines (FB1, FB2). Scale bar: 100 µm. Validation of CAF transformation of 2D-cultured fibroblasts was performed 72h following TGF-β1 treatment, as shown by changes in the expression of alpha-smooth muscle actin (αSMA) and fibroblast-activation protein (FAP) at the mRNA (B) and protein level (C) (n = 3). (D) Validation of CAF transformation of TGF-β1-treated and subsequently 3D-cultured fibroblasts, as shown by changes in the expression of αSMA and FAP at the protein level. Data are presented as mean ± SD. ****, p≤0.0001.

Supplementary Material 6 Fig. S2: Representative dose-response curves of human NSCLC cell lines H2228, H3122, and A549 following 72 h of treatment with the ALK-TKIs brigatinib and lorlatinib.

Supplementary Material 7 Fig. S3: (A) Gating strategy for the analysis of lung cancer cell death rates. (B) Representative dot plots showing the amount of dead or dying H2228 cells derived from dissociated (non-)treated homo- and heterotypic tumor spheroids following flow cytometric cell death analysis. Quantification of cell death rates of H2228 (C) and H3122 (D) cells as given by the sum of early (Q3) and late apoptotic/necrotic (Q2) cells (n = 3). Data are presented as mean ± SD. ##, p ≤ 0.01; ###, p ≤ 0.001 compared to the corresponding DMSO controls. **, p ≤ 0.01; ***, p ≤ 0.001 in comparison to brigatinib-treated mono-cultures. FVD, fixable viability dye.

Supplementary Material 8 Fig. S4: (A) Representative dot plots illustrating the distribution of H2228 cells in the G0, G1, S, and G2-M cell cycle phases derived from dissociated (non-)treated homo- and heterotypic tumor spheroids following flow cytometric cell cycle analysis. Quantification of the portion of H2228 (B) and H3122 (C) cells according to their cell cycle phase status (n = 3). Data are presented as mean ± SD. #, p≤0.05; ##, p≤0.01; ###, p≤0.001; ####, p≤0.0001 for cells in the G0-phase compared to the corresponding DMSO controls. **, p≤0.01; ***, p≤0.001 for cells in the G0-phase in comparison to brigatinib-treated mono-cultures. PI, propidium iodide.

Supplementary Material 9 Fig. S5: Heatmaps depicting the top ten significantly expressed marker genes of each cell cluster identified following clustering analysis of H2228 (A) and H3122 (B) samples.

Supplementary Material 10 Fig. S6: (A) Bar graphs depicting the total number of differentially expressed genes (DEGs) observed between ALK-inhibited mono-cultured and FB2 co-cultured H2228 and H3122 cells. Blue bars represent the number of downregulated genes, whereas the green bars represent upregulated genes. (B) Venn diagram illustrating the DEGs upregulated in FB2 co-cultures versus mono-cultures between brigatinib- or lorlatinib-treated H2228 and H3122 cells.

Supplementary Material 11 Fig. S7: Heatmap of differentially expressed genes upon ALK inhibition. The set of 79 upregulated genes overlapping between brigatinib- and lorlatinib-treated H2228 and H3122 co-cultures in comparison to mono-cultures are color-coded according to their log2-fold-change expression values.

Supplementary Material 12 Fig. S8: Expression heatmap of fatty acid metabolism-related genes in single-cell transcriptome datasets of brigatinib-treated H2228 and H3122 cells.

Supplementary Material 13 Fig. S9: Average expression of the lipogenic markers ACLY, ACACA, FASN, SCD1, and SREBF1 (termed as Fatty acid score) was projected on UMAP plots of H2228 (A) and H3122 (B) samples to identify enrichment in a cluster-dependent manner. Red indicates maximum gene expression, while grey indicates low or no expression of the selected genes in log-normalized UMI counts.

Supplementary Material 14 Fig. S10: Co-cultivation with FB2-CAFs influences the expression of fatty acid metabolism-related targets upon ALK signaling perturbation via brigatinib (A) and lorlatinib (B) in H2228 and H3122 cells (n = 3). Data are presented as mean ± SD. #, p≤0.05; ##, p≤0.01; ####, p≤0.0001 compared to corresponding DMSO controls. *, p≤0.05; **, p≤0.01; ***, p≤0.001; ****, p≤0.0001 compared to brigatinib- or lorlatinib-treated mono-cultures, respectively.

Supplementary Material 15 Fig. S11: (A) Representative phase contrast images of homotypic H2228 and H3122 lung tumor spheroids treated according to the indicated conditions for 72 h. Scale bar: 200 µm. Changes in spheroid size following lorlatinib treatment in the absence or presence of FB2-CAF-conditioned medium were investigated by analyzing the spheroid areas of imaged H2228 (B) and H3122 (C) lung tumor spheroids. Spheroid size values of twelve individual spheroids are given per experimental group (n = 3). All data are presented as mean ± SD. ####, p≤0.0001 compared to DMSO controls. **, p≤0.01; ****, p≤0.0001 in comparison to lorlatinib treatment alone.

Supplementary Material 16 Fig. S12: Validation of siRNA-mediated knockdown in FB2 fibroblasts using quantitative real-time PCR analysis of GAPDH, HGF, NRG1α, and NRG1β mRNA levels. FB2 fibroblasts were transfected with siRNAs targeting HGF (siHGF_1–3), NRG1 (siNRG1_1–3), a scrambled control siRNA, or a GAPDH-targeting siRNA as positive control (n = 2). Data are presented as mean ± SD.

Supplementary Material 17 Fig. S13: Heatmap illustrating unsupervised hierarchical clustering of lipid species abundances in H3122 spheroids across all replicates (n = 3) and treatment conditions. Data analyzed via MetaboAnalyst 5.0 (86).

Supplementary Material 18 Fig. S14: (A) Log2 ratios depicting the alterations in saturation index induced by lorlatinib. The saturation index is calculated on the basis of the sum of species with the same (un)saturation level (n = 3). (B) Cell viability analysis following the combined treatment of H2228 tumor spheroids with fatostatin, lorlatinib, and fibroblast CM (n = 3). (C) Quantification of lipid peroxidation in H2228 spheroids using C11 BODIPY. Hydrogen peroxide (H2O2) served as a positive control. All data are presented as mean ± SD. #, p ≤ 0.05; ####, p ≤ 0.0001 compared to corresponding DMSO controls. ns, not significant; *, p ≤ 0.05; **, p ≤ 0.01; ***, p ≤ 0.001; ****, p ≤ 0.0001 in comparison to lorlatinib treatment alone.

Supplementary Material 19 Supplementary Table S2: Quality control metrics following scRNA-sequencing applying the 10x Genomics plattform

Supplementary Material 20 Supplementary Table S3: Cell numbers of identified cell cluster

Supplementary Material 21 Supplementary Table S1: Average IC50- and IC75-values of dose-response curves

## Data Availability

Single-cell RNA sequencing data was uploaded to the European Genome-Phenome Archive (EGA), accessible under EGAS50000000135, and is available under controlled access. All remaining data supporting the findings of this study are available within the paper and its Supplementary Information.

## Abbreviations

5-PL: Five-parameter logistic
ACACA: Acetyl-CoA carboxylase alpha
ACLY: ATP citrate lyase
ACTB: β-actin
AKT: Protein kinase B
ALK: Anaplastic lymphoma kinase
ALKi: ALK inhibitors
ATP: Adenosine triphosphate
BRAF: V-raf murine sarcoma viral oncogene homolog B
BSA: Bovine serum albumin
CAF: Cancer-associated fibroblast
CFSE: Carboxyfluorescein succinimidyl ester
CM: Conditioned medium
CoA: Coenzyme A
DMEM: Dulbecco’s Modified Eagle Medium
DMSO: Dimethyl sulfoxide
DPBS: Dulbecco’s Phosphate-Buffered Saline
ECM: Extracellular matrix
EDTA: Ethylenediaminetetraacetic acid
EGFR: Epidermal growth factor receptor
ELISA: Enzyme-linked immunosorbent assay
EMEM: Eagle’s Minimal Essential Medium
EML4: Echinoderm microtubule-associated protein-like 4
ERBB3/HER3: Human epidermal growth factor receptor
ERK: Extracellular-signal regulated kinase
FAP: Fibroblast activation protein
FASN: Fatty acid synthase
FCS: Fetal calf serum
FDR: False discovery rate
FVD: Fixable Viability Dye
GAPDH: Glyceraldehyde 3-phosphate dehydrogenase
GEM: Gel bead in emulsion
GFR: Growth-factor reduced
GSEA: Gene set enrichment analysis
HEPES: 4-(2-hydroxyethyl)-1-piperazineethanesulfonic acid
HGF: Hepatocyte growth factor
IC: Inhibitory concentration
KRAS: V-Ki-Ras2 Kirsten rat sarcoma viral oncogene homolog
MACS: Magnetic-activated cell sorting
MSigDB: Molecular Signatures Database
NFDM: Non-fat dry milk
NRG1: Neuregulin 1
NSCLC: Non-small-cell carcinoma
OPG: Osteoprotegerin
PAGE: Polyacrylamide gel electrophoresis
PC: Phosphatidylcholine
PCA: Principal component analysis
PD: Progressive disease
PE: Phosphatidylethanolamine
PI: Propidium iodide
PI3K: Phosphoinositide 3-kinase
PS: Phosphatidylserine
PUFA: Polyunsaturated fatty acids
RIPA: Radioimmunoprecipitation assay
ROS: Reactive oxygen species
RPMI: Roswell Park Memorial Institute
RTK: Receptor tyrosine kinase
RSL3: RAS-selective lethal 3
S6K: Ribosomal protein S6 kinase 1
SCD: Stearoyl-CoA desaturase-1
SDS: Sodium dodecyl sulfate
SMA: Smooth muscle actin
SNP: Single-nucleotide polymorphism
SREBP: Sterol regulatory element-binding protein
TBS: Tris-buffered saline
TGF: Transforming growth factor-beta
TKI: Tyrosine kinase inhibitor
TME: Tumor microenvironment
TN: Treatment-naive
ULA: Ultra-low attachment
UMAP: Uniform manifold approximation and projection
UMI: Unique-molecular identifier
