# Supplementary figures and images for: Cancer-associated fibroblasts promote drug resistance in ALK-driven lung adenocarcinoma cells by upregulating lipid biosynthesis
Source: Cancer Metab. 2025 Jun 16;13:28. doi: 10.1186/s40170-025-00400-7 (PMC12168422; doi:10.1186/s40170-025-00400-7)

**FB2**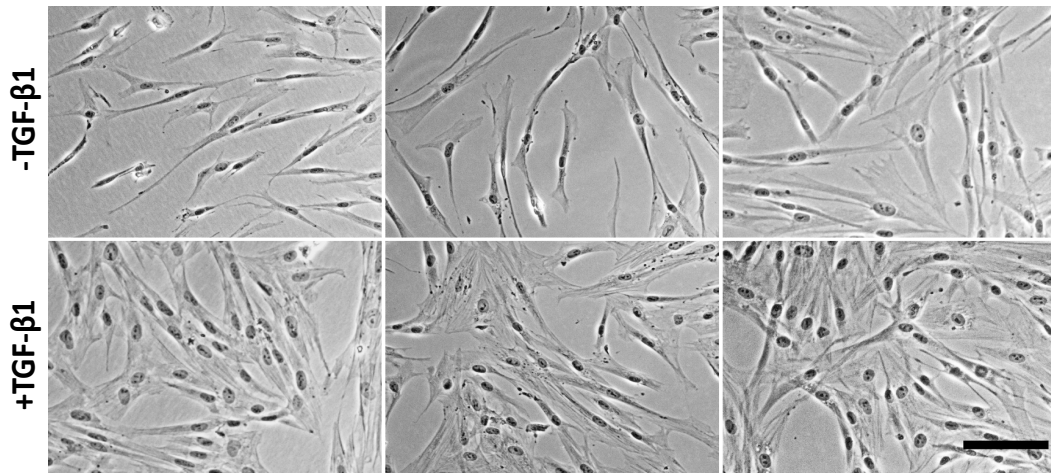

# B

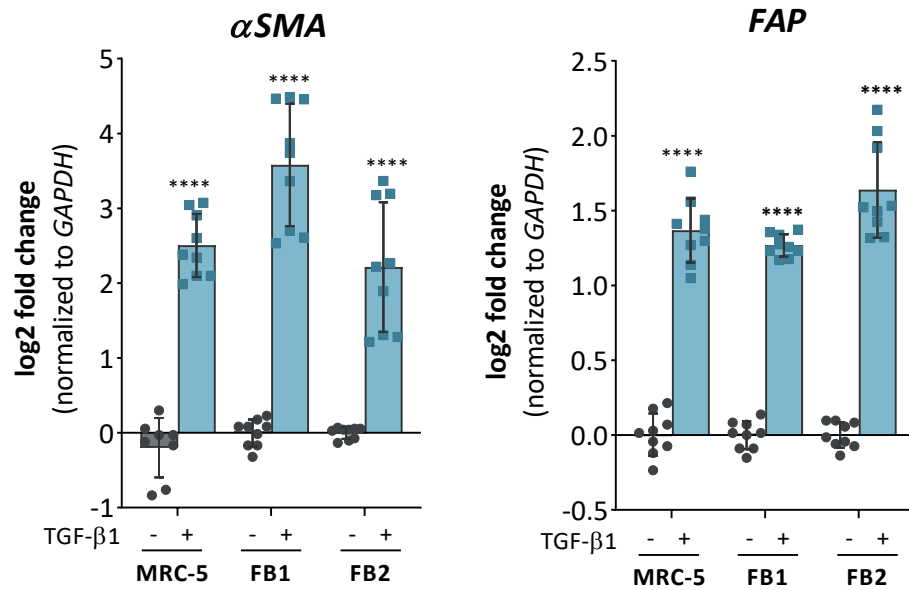

**C**

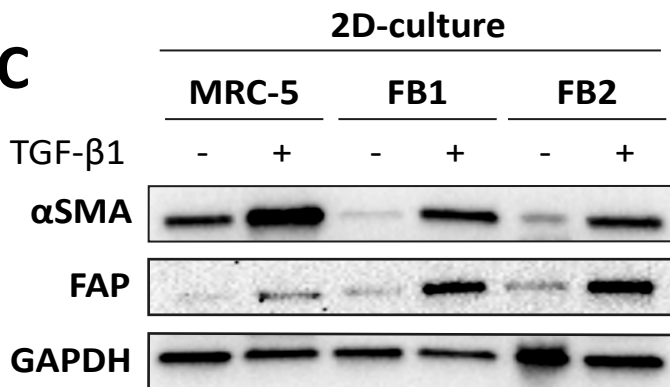

D

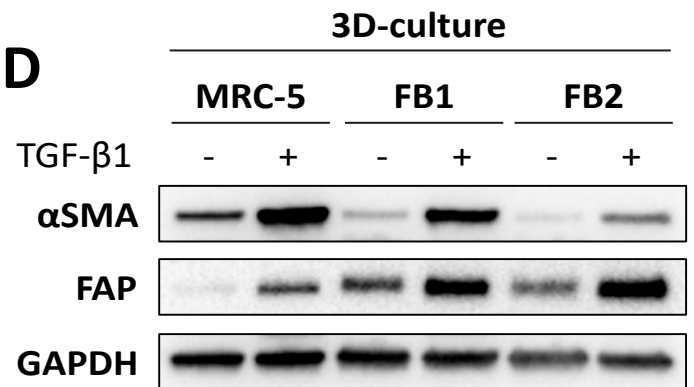

Supplement: Supplementary file 1 — Supplementary Material 1 Additional File 1: Raw unedited blots with highlighted bands of the western blots shown in panel (A) of Fig. 6. [file 40170_2025_400_MOESM1_ESM.pdf]

### Brigatinib

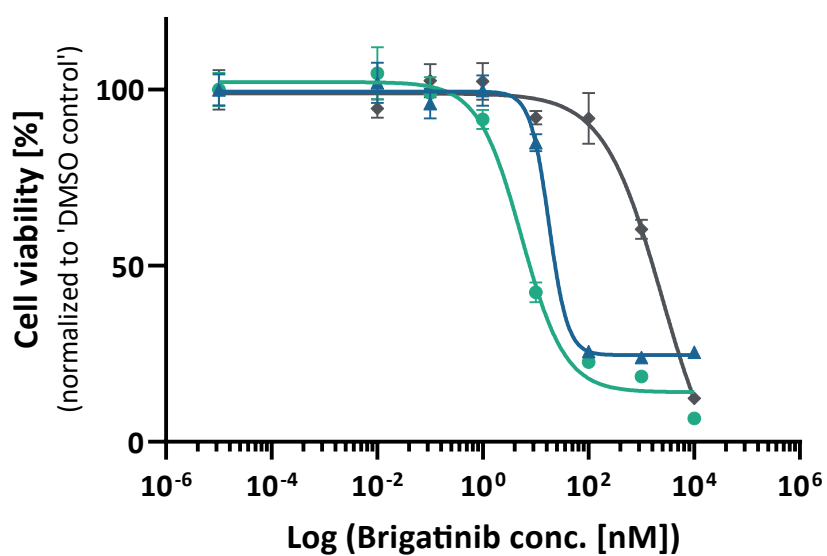

### Lorlatinib

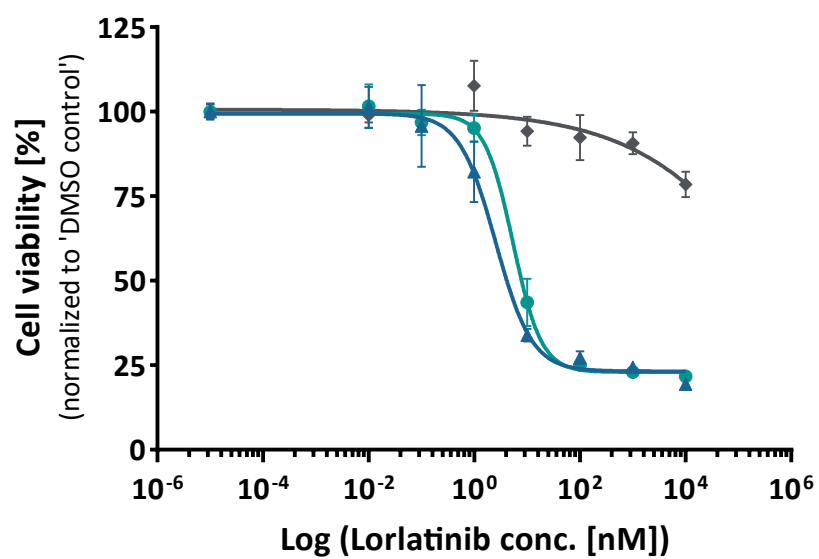

—▲— H2228    —●— H3122    —◆— A549

Supplement: Supplementary file 2 — Supplementary Material 2 Additional File 2: Raw unedited blots with highlighted bands of the western blots shown in panel (B) of Fig. 6. [file 40170_2025_400_MOESM2_ESM.pdf]

**A**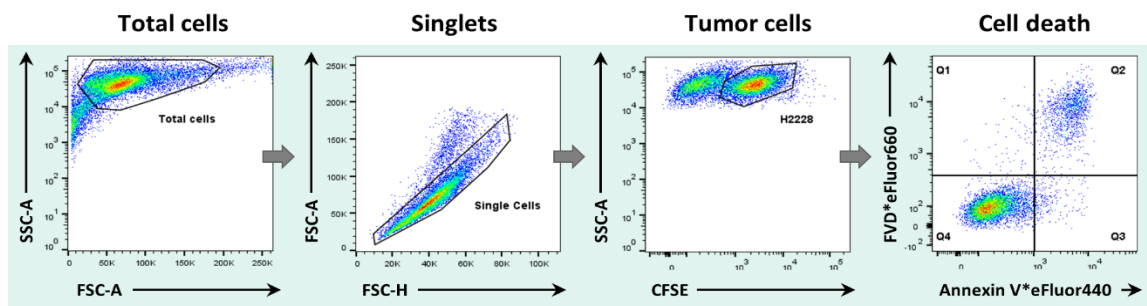**B**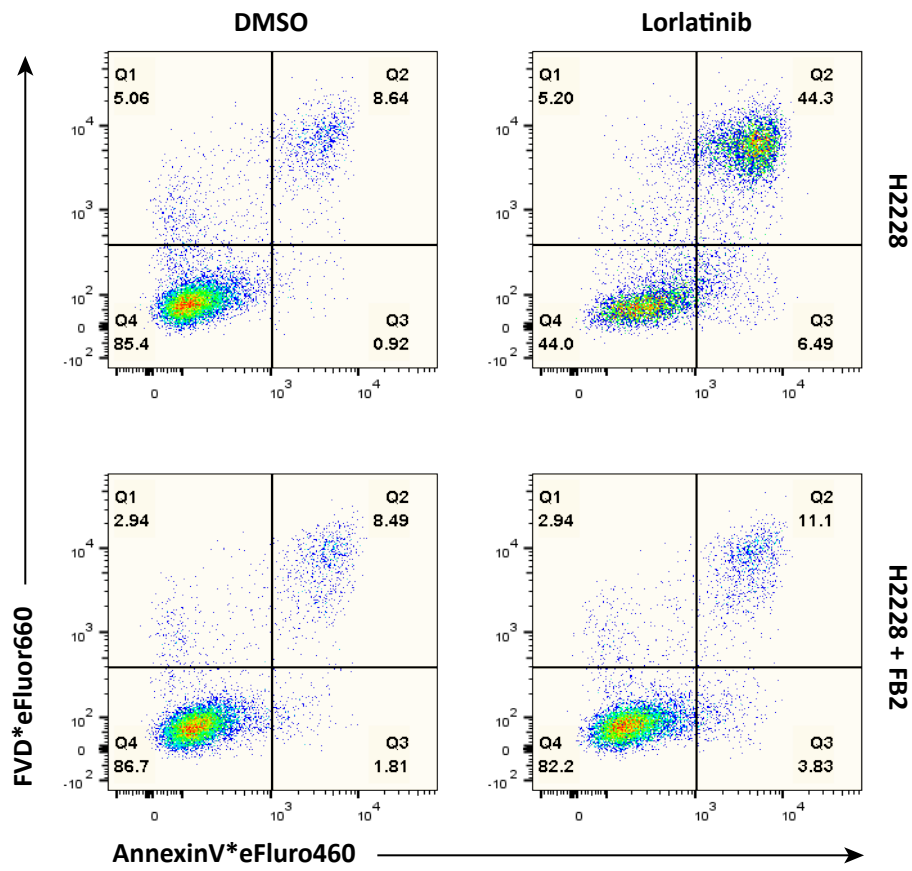**C**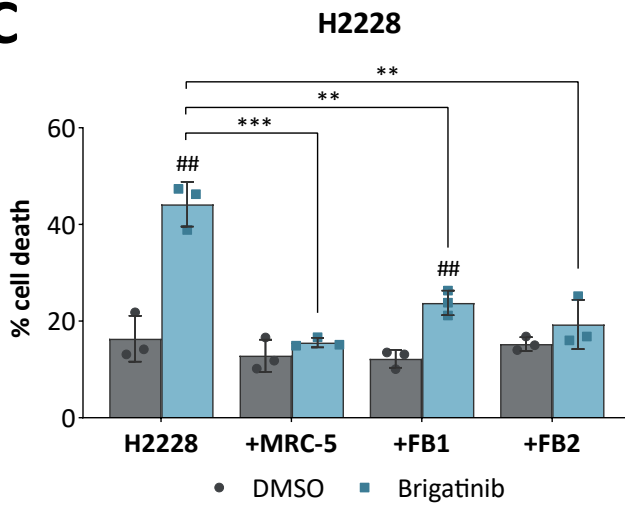**D**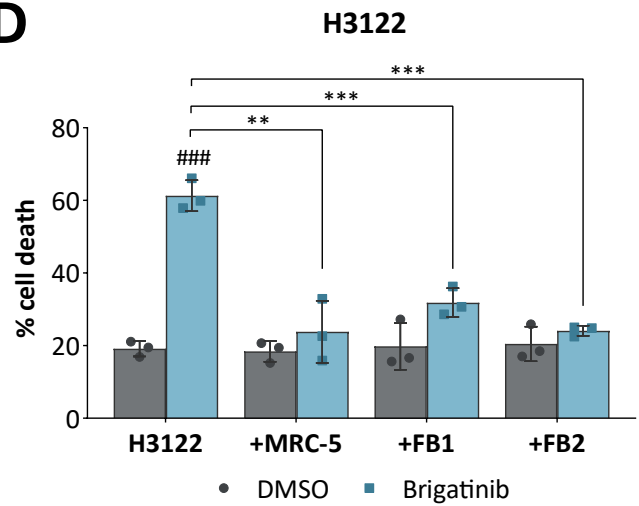

Supplement: Supplementary file 3 — Supplementary Material 3 Additional File 2: Raw unedited blots with highlighted bands of the western blots shown in panel (B) of Fig. 6. [file 40170_2025_400_MOESM3_ESM.pdf]

**A**

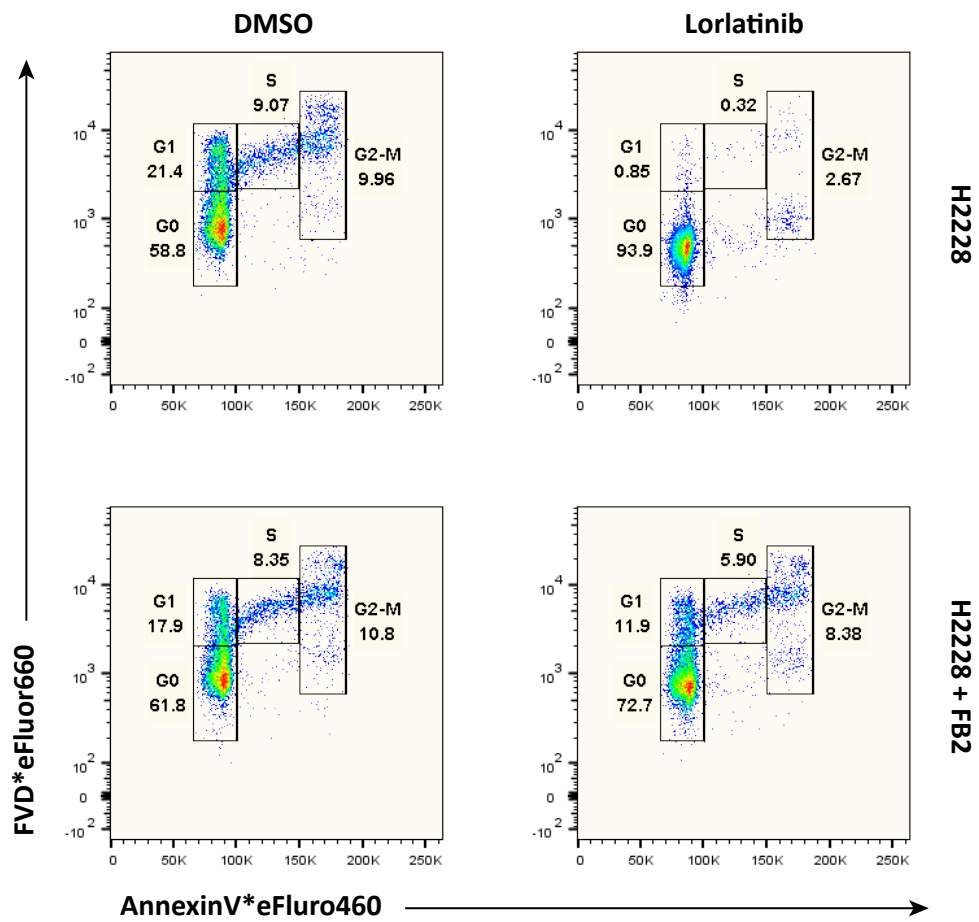

**B**

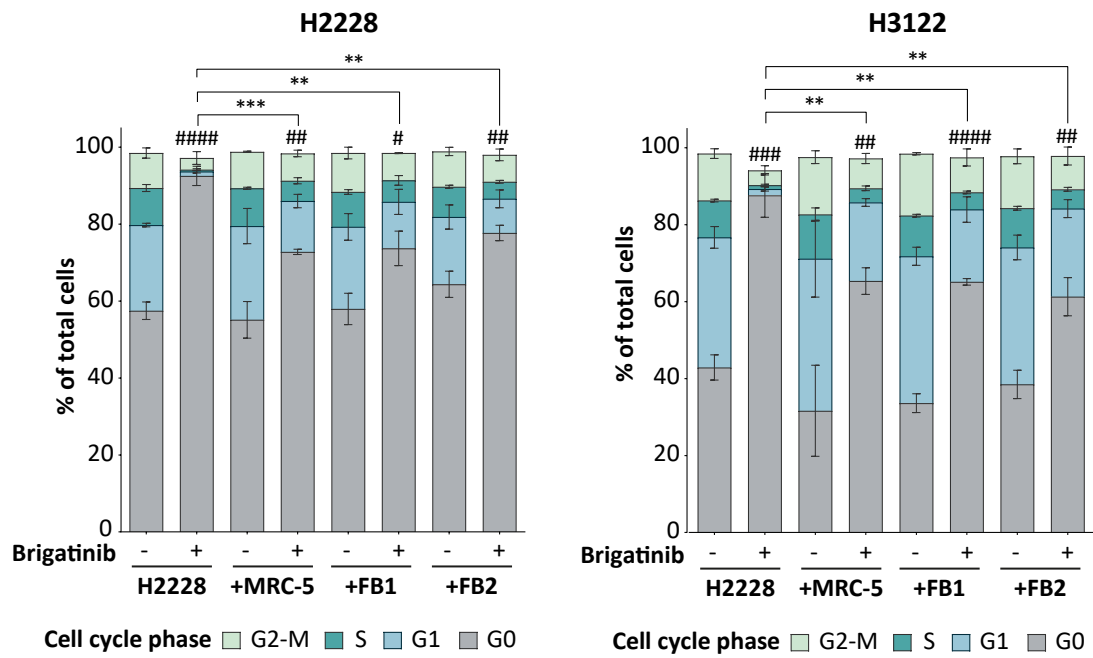

Supplement: Supplementary file 4 — Supplementary Material 4 Additional File 4: Raw unedited blots with highlighted bands of the western blots shown in panel (B) of Fig. 7. [file 40170_2025_400_MOESM4_ESM.pdf]

A

H2228

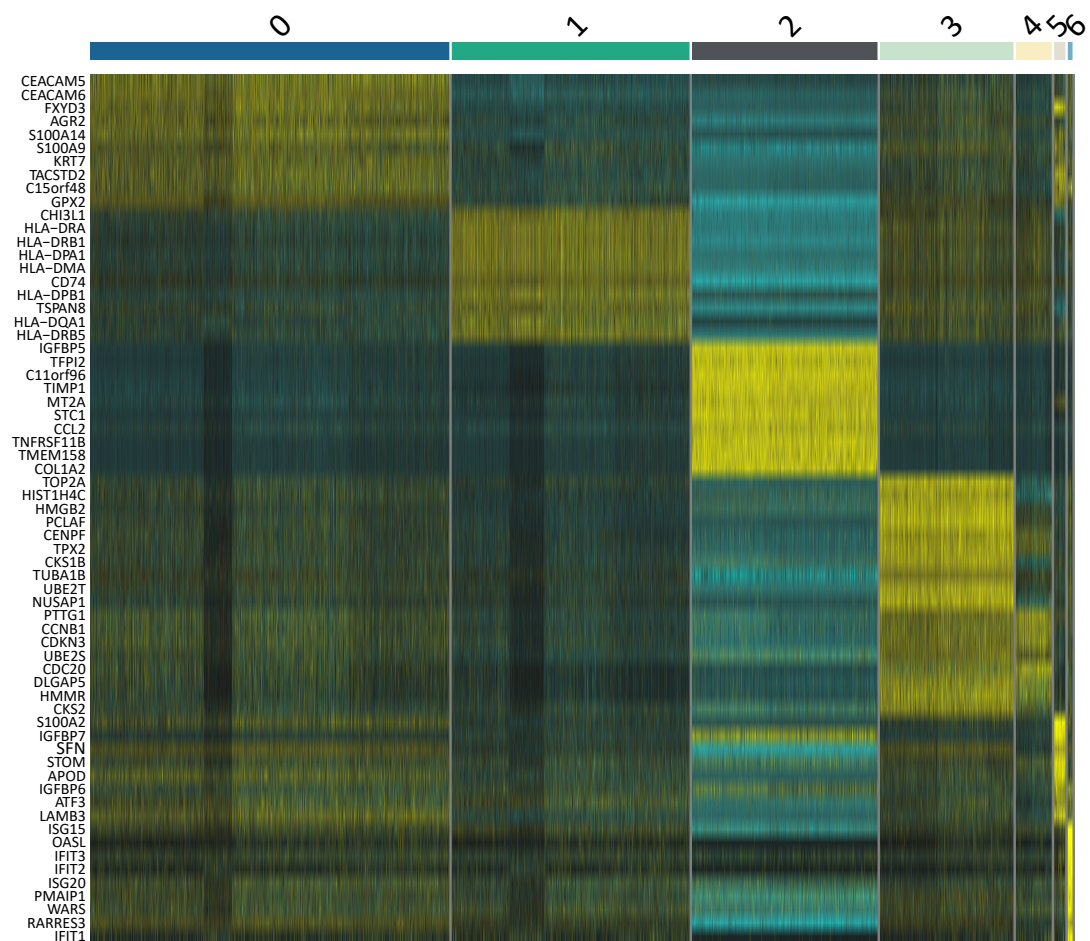

Expression

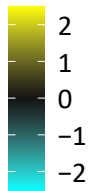

B

H3122

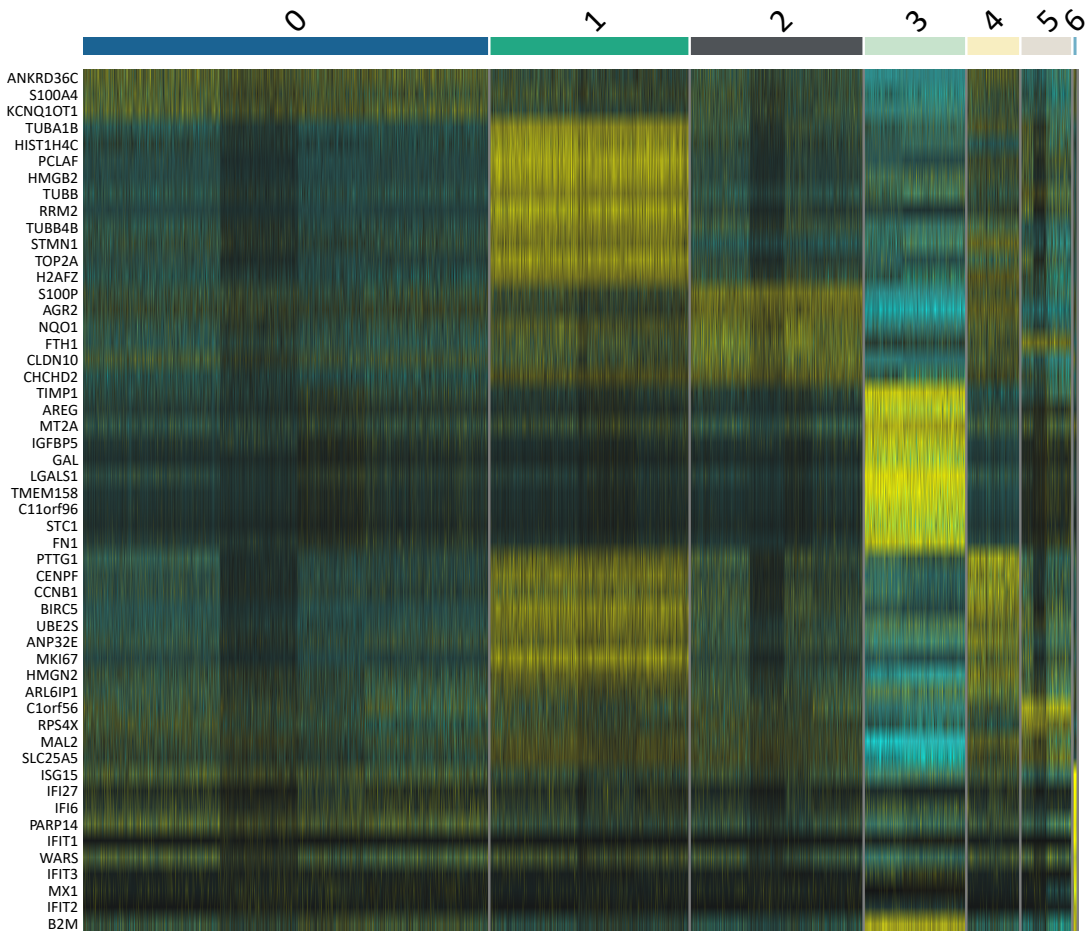

Expression

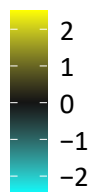

Supplement: Supplementary file 5 — Supplementary Material 5 Fig. S1: (A) Phase-contrast images of untreated (top) versus transforming growth factor-beta 1 (TGF-β1)-stimulated (bottom) MRC-5 fibroblasts and two primary fibroblast lines (FB1, FB2). Scale bar: 100 µm. Validation of CAF transformation of 2D-cultured fibroblasts was performed 72h following TGF-β1 treatment, as shown by changes in the expression of alpha-smooth muscle actin (αSMA) and fibroblast-activation protein (FAP) at the mRNA (B) and protein level (C) (n = 3). (D) Validation of CAF transformation of TGF-β1-treated and subsequently 3D-cultured fibroblasts, as shown by changes in the expression of αSMA and FAP at the protein level. Data are presented as mean ± SD. ****, p≤0.0001. [file 40170_2025_400_MOESM5_ESM.pdf]

**A**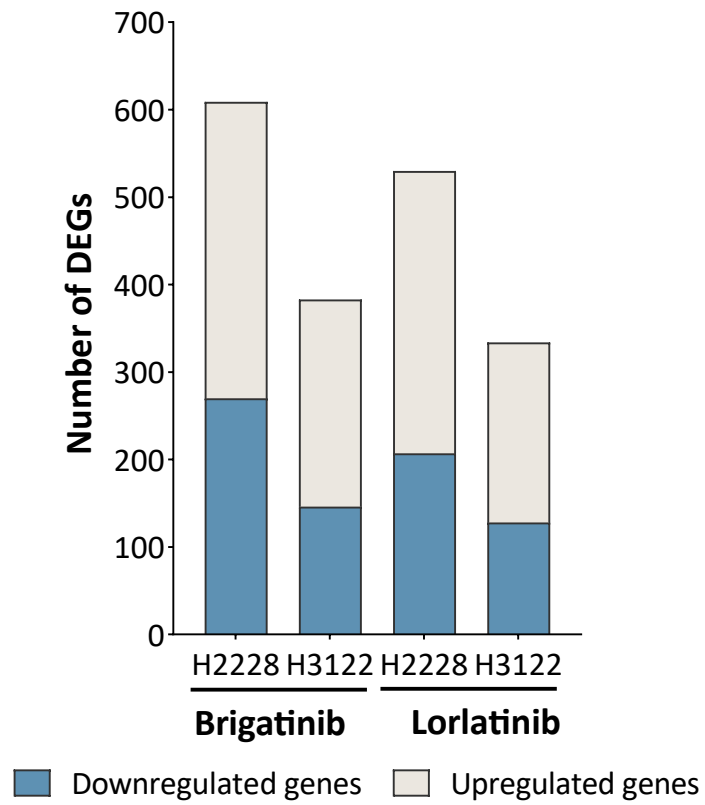**B**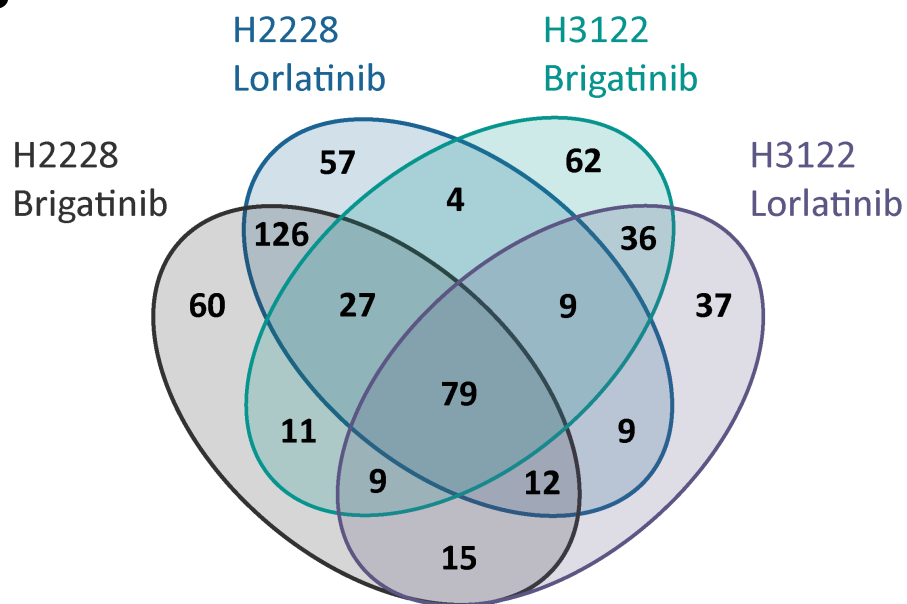

Supplement: Supplementary file 6 — Supplementary Material 6 Fig. S2: Representative dose-response curves of human NSCLC cell lines H2228, H3122, and A549 following 72 h of treatment with the ALK-TKIs brigatinib and lorlatinib. [file 40170_2025_400_MOESM6_ESM.pdf]

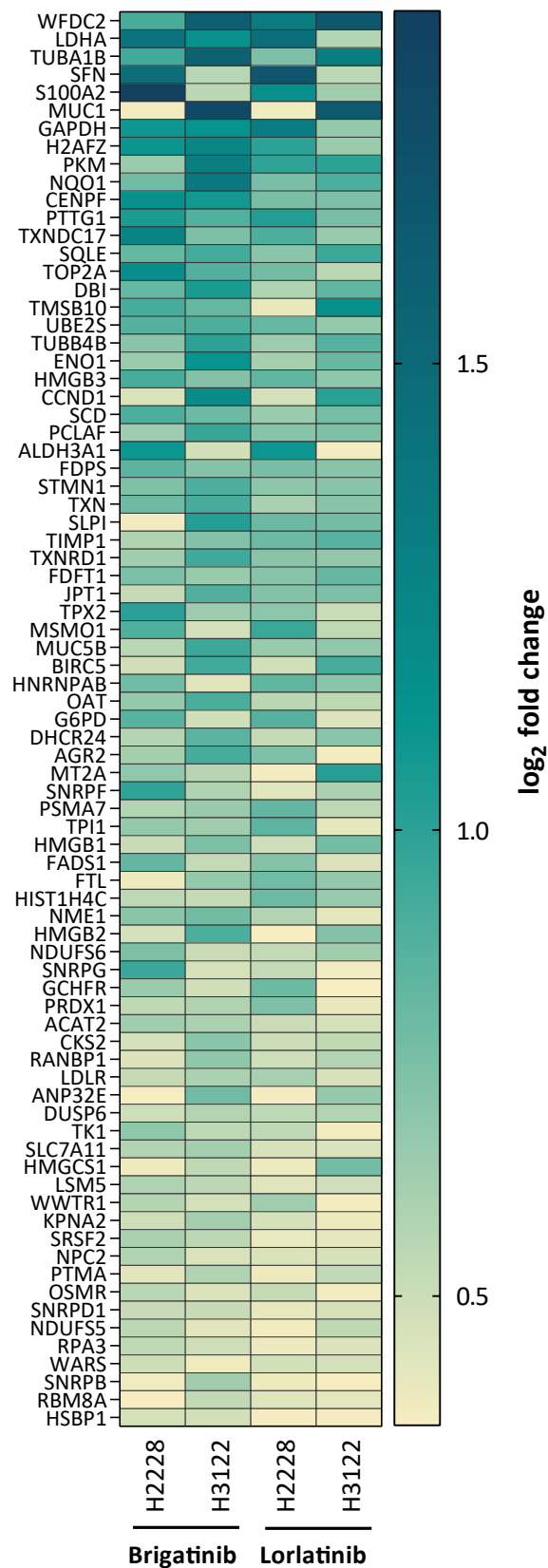

Supplement: Supplementary file 7 — Supplementary Material 7 Fig. S3: (A) Gating strategy for the analysis of lung cancer cell death rates. (B) Representative dot plots showing the amount of dead or dying H2228 cells derived from dissociated (non-)treated homo- and heterotypic tumor spheroids following flow cytometric cell death analysis. Quantification of cell death rates of H2228 (C) and H3122 (D) cells as given by the sum of early (Q3) and late apoptotic/necrotic (Q2) cells (n = 3). Data are presented as mean ± SD. ##, p ≤ 0.01; ###, p ≤ 0.001 compared to the corresponding DMSO controls. **, p ≤ 0.01; ***, p ≤ 0.001 in comparison to brigatinib-treated mono-cultures. FVD, fixable viability dye. [file 40170_2025_400_MOESM7_ESM.pdf]

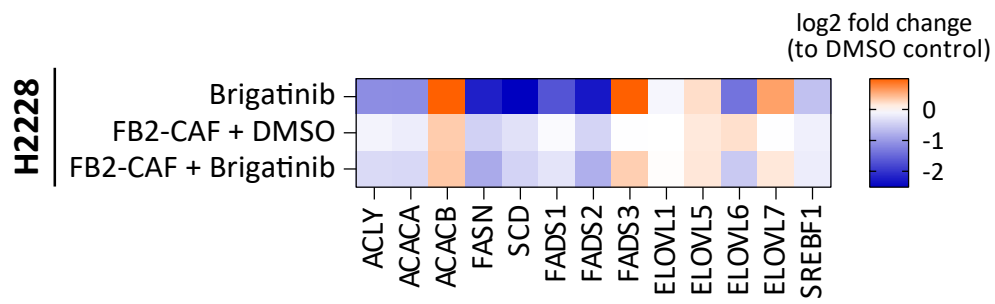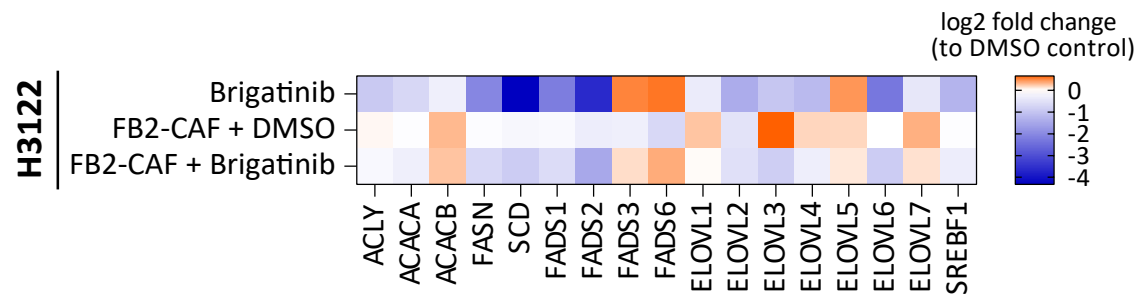

Supplement: Supplementary file 8 — Supplementary Material 8 Fig. S4: (A) Representative dot plots illustrating the distribution of H2228 cells in the G0, G1, S, and G2-M cell cycle phases derived from dissociated (non-)treated homo- and heterotypic tumor spheroids following flow cytometric cell cycle analysis. Quantification of the portion of H2228 (B) and H3122 (C) cells according to their cell cycle phase status (n = 3). Data are presented as mean ± SD. #, p≤0.05; ##, p≤0.01; ###, p≤0.001; ####, p≤0.0001 for cells in the G0-phase compared to the corresponding DMSO controls. **, p≤0.01; ***, p≤0.001 for cells in the G0-phase in comparison to brigatinib-treated mono-cultures. PI, propidium iodide. [file 40170_2025_400_MOESM8_ESM.pdf]

**A**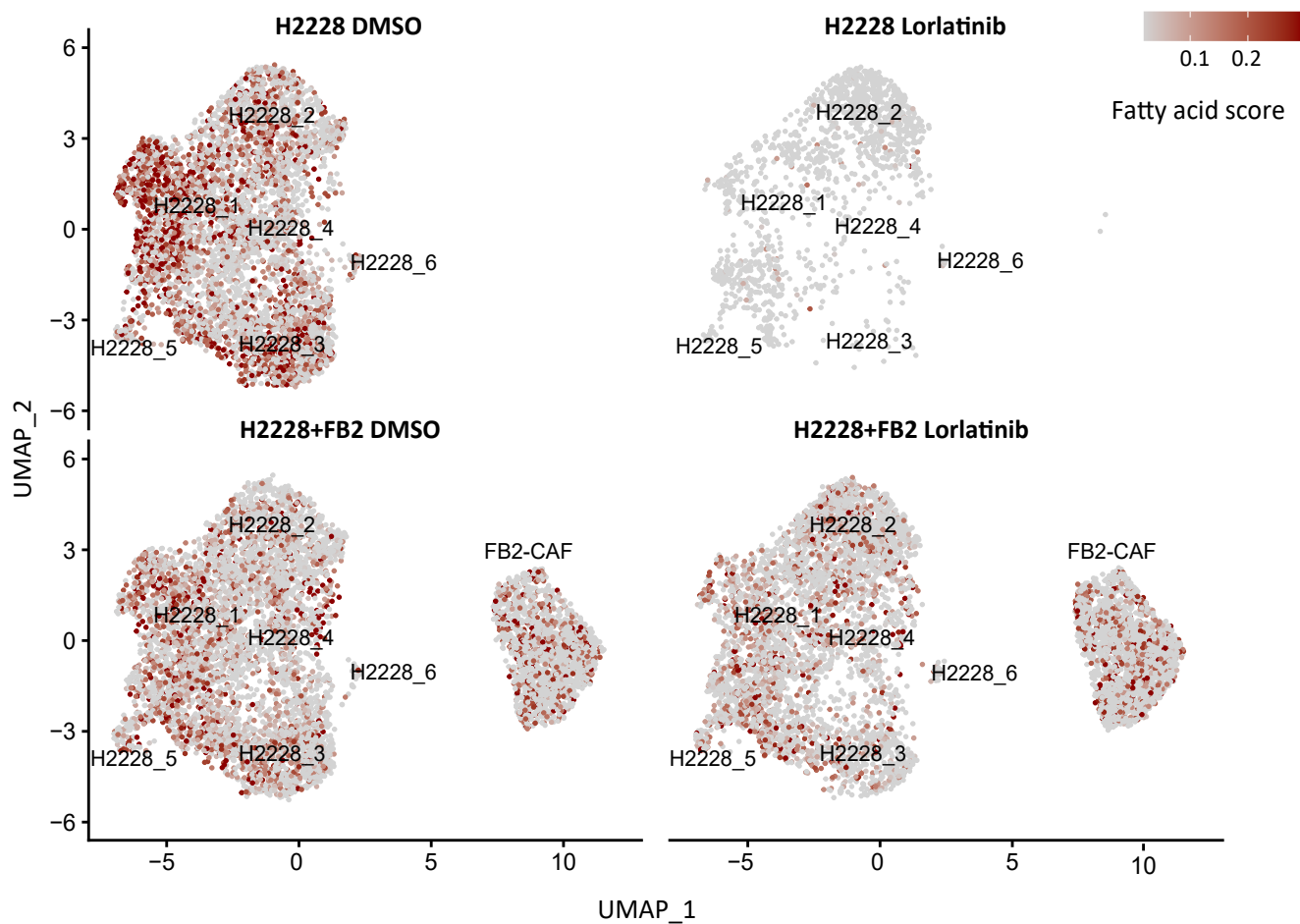**B**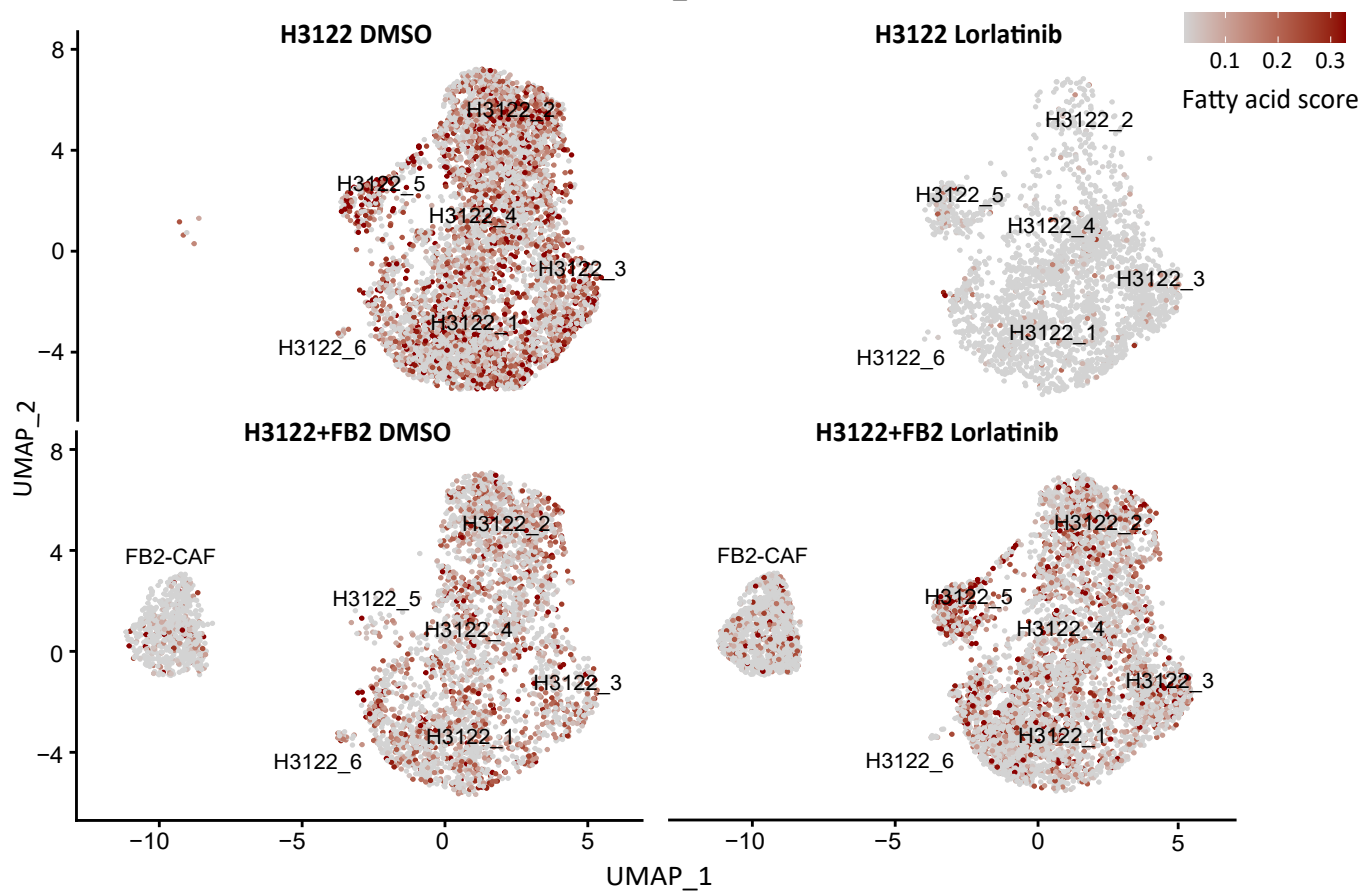

Supplement: Supplementary file 9 — Supplementary Material 9 Fig. S5: Heatmaps depicting the top ten significantly expressed marker genes of each cell cluster identified following clustering analysis of H2228 (A) and H3122 (B) samples. [file 40170_2025_400_MOESM9_ESM.pdf]

**A**

**H2228**

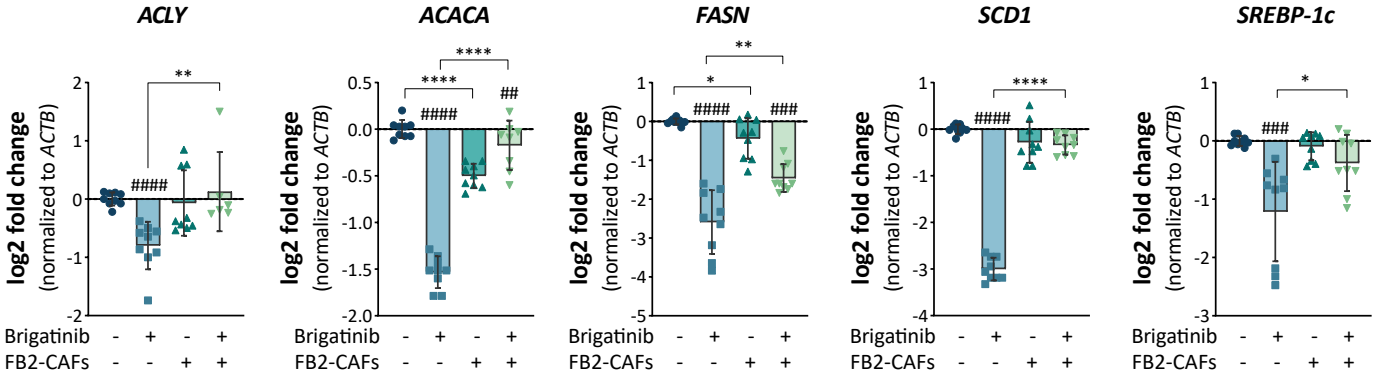

**H3122**

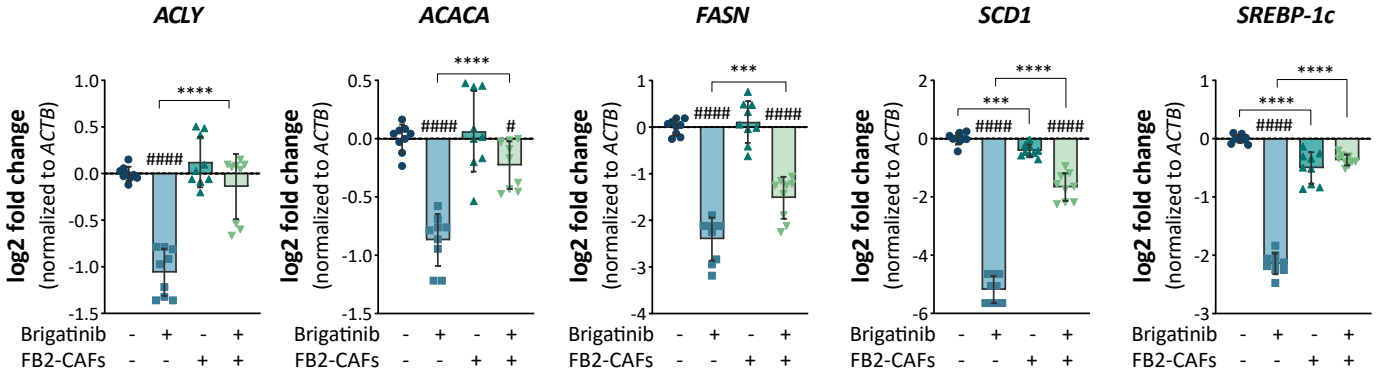

**B**

**H2228**

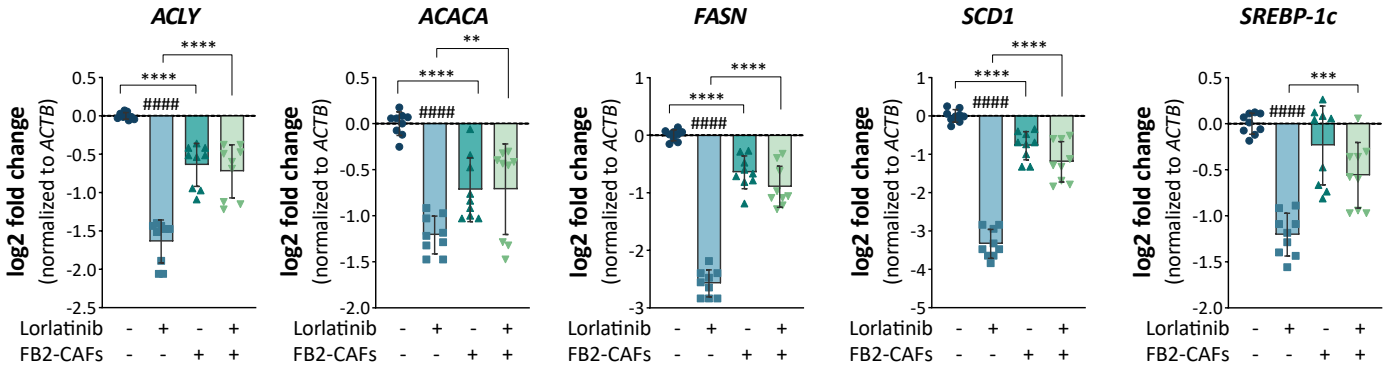

**H3122**

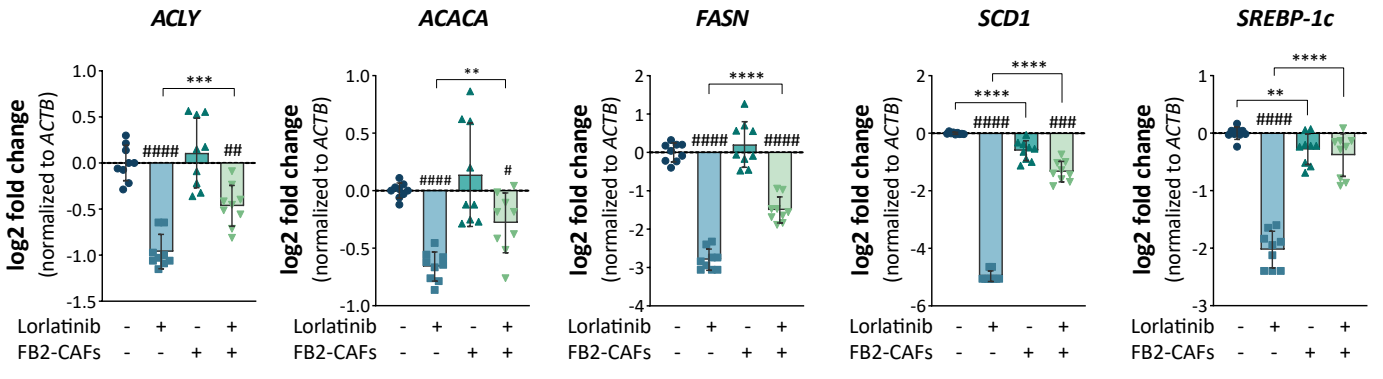

Supplement: Supplementary file 10 — Supplementary Material 10 Fig. S6: (A) Bar graphs depicting the total number of differentially expressed genes (DEGs) observed between ALK-inhibited mono-cultured and FB2 co-cultured H2228 and H3122 cells. Blue bars represent the number of downregulated genes, whereas the green bars represent upregulated genes. (B) Venn diagram illustrating the DEGs upregulated in FB2 co-cultures versus mono-cultures between brigatinib- or lorlatinib-treated H2228 and H3122 cells. [file 40170_2025_400_MOESM10_ESM.pdf]

**A**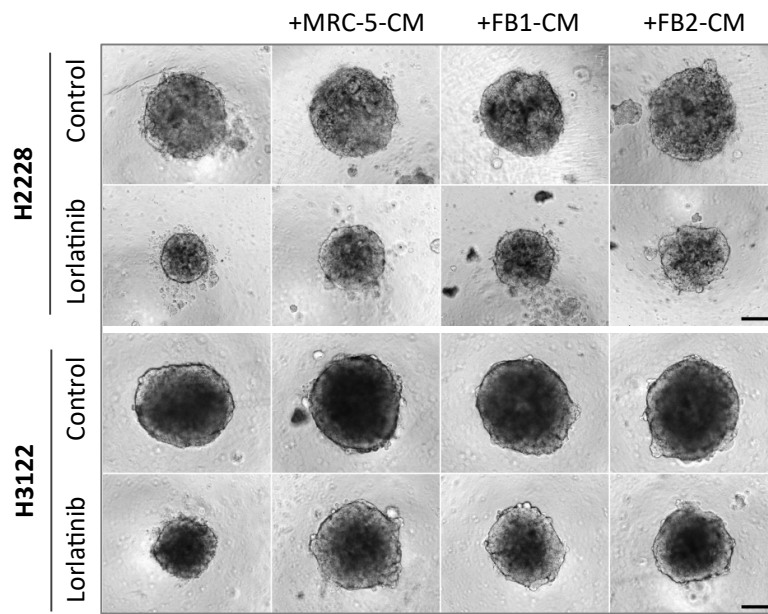**B**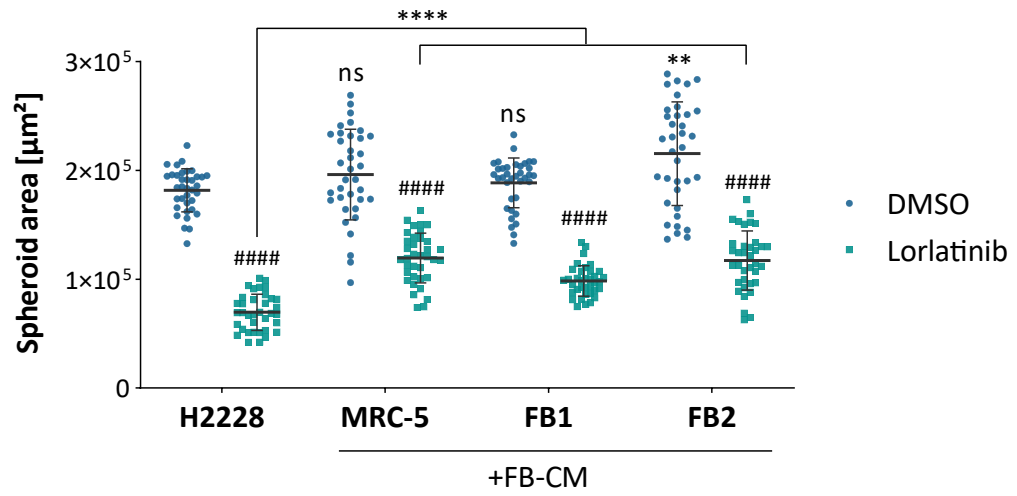**C**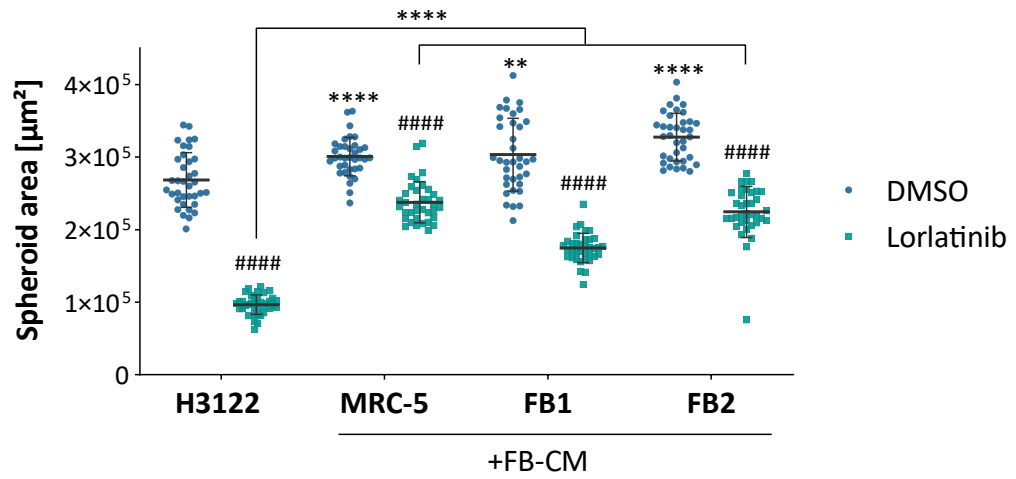

Supplement: Supplementary file 11 — Supplementary Material 11 Fig. S7: Heatmap of differentially expressed genes upon ALK inhibition. The set of 79 upregulated genes overlapping between brigatinib- and lorlatinib-treated H2228 and H3122 co-cultures in comparison to mono-cultures are color-coded according to their log2-fold-change expression values. [file 40170_2025_400_MOESM11_ESM.pdf]

**GAPDH**

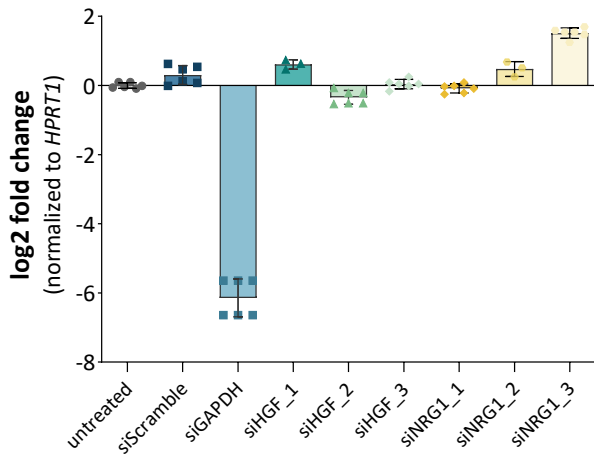

**HGF**

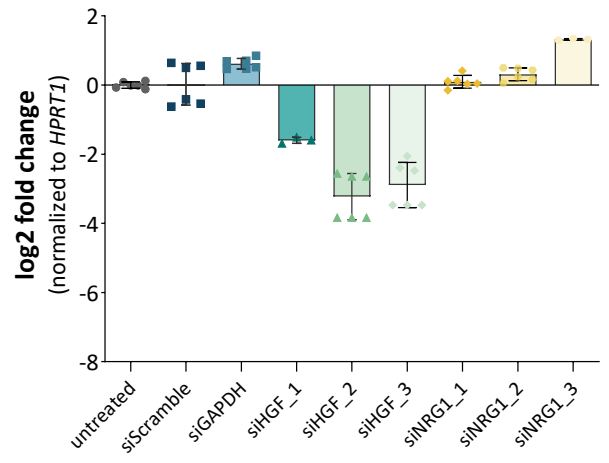

**NRG1 $\alpha$**

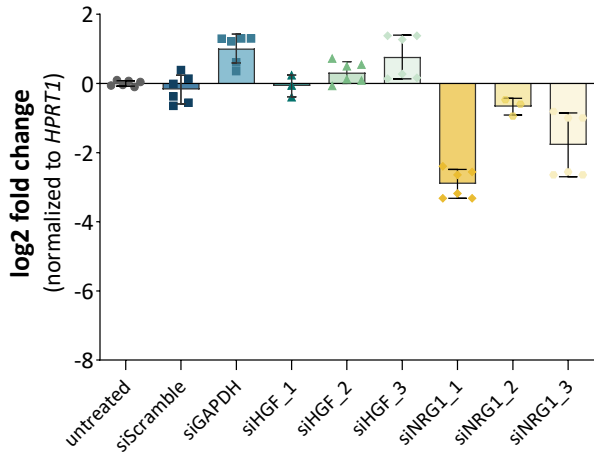

**NRG1 $\beta$**

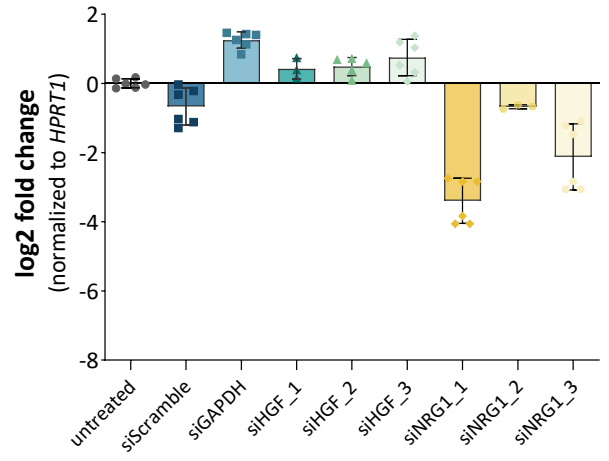

Supplement: Supplementary file 12 — Supplementary Material 12 Fig. S8: Expression heatmap of fatty acid metabolism-related genes in single-cell transcriptome datasets of brigatinib-treated H2228 and H3122 cells. [file 40170_2025_400_MOESM12_ESM.pdf]

**A**

• H3122    ■ H3122+FB2-CM

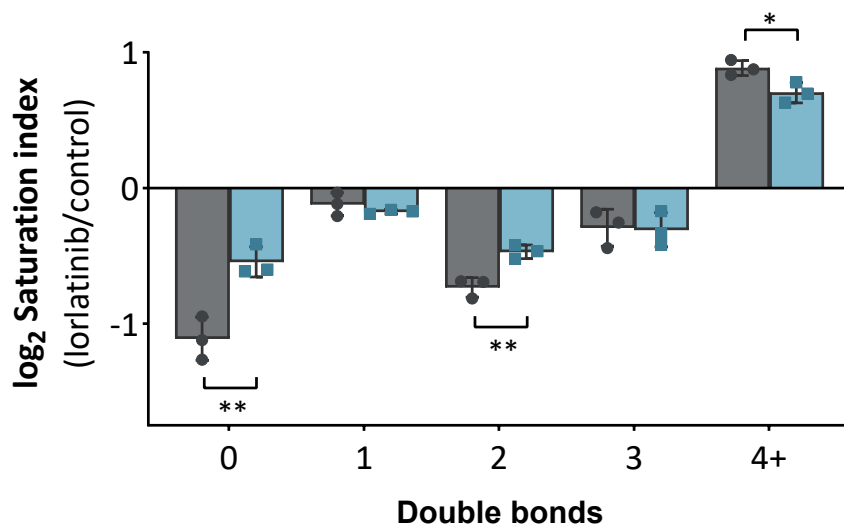**B**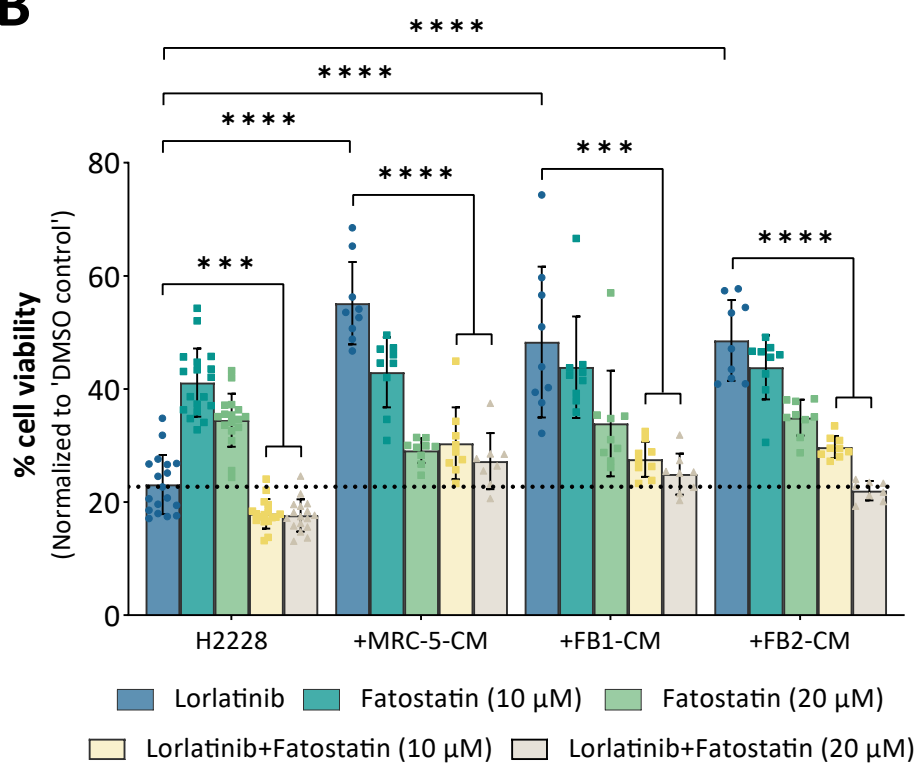**C**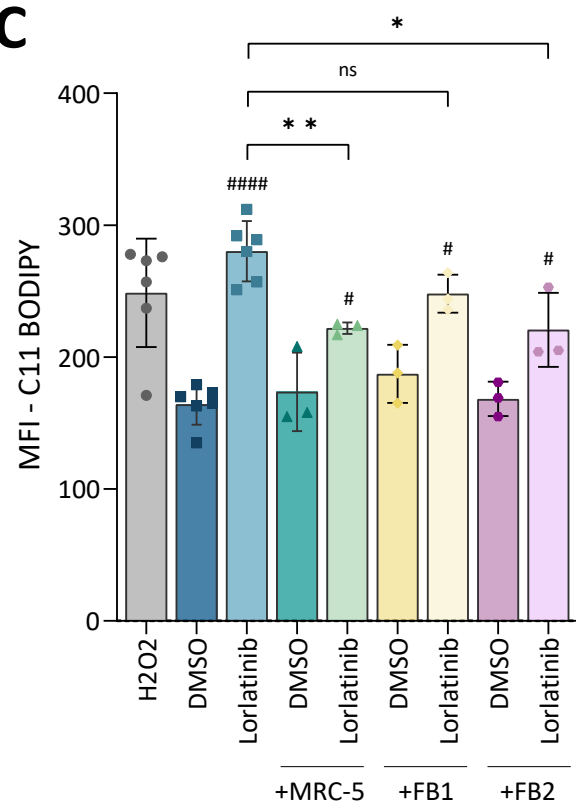**D**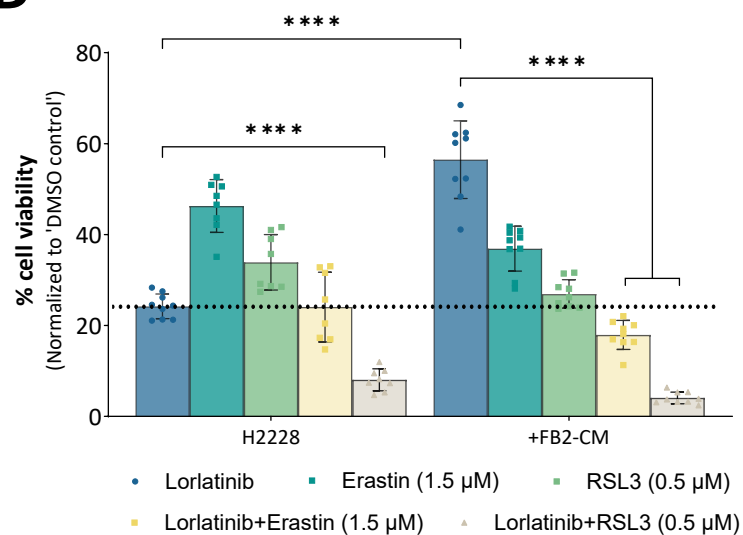**E**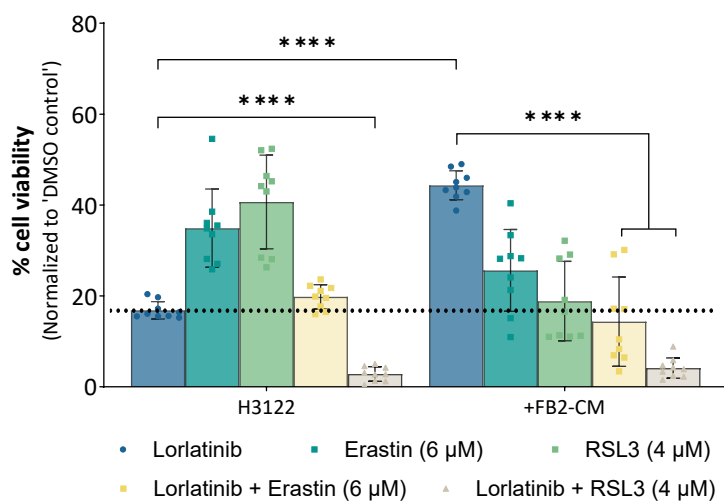

Supplement: Supplementary file 13 — Supplementary Material 13 Fig. S9: Average expression of the lipogenic markers ACLY, ACACA, FASN, SCD1, and SREBF1 (termed as Fatty acid score) was projected on UMAP plots of H2228 (A) and H3122 (B) samples to identify enrichment in a cluster-dependent manner. Red indicates maximum gene expression, while grey indicates low or no expression of the selected genes in log-normalized UMI counts. [file 40170_2025_400_MOESM13_ESM.pdf]

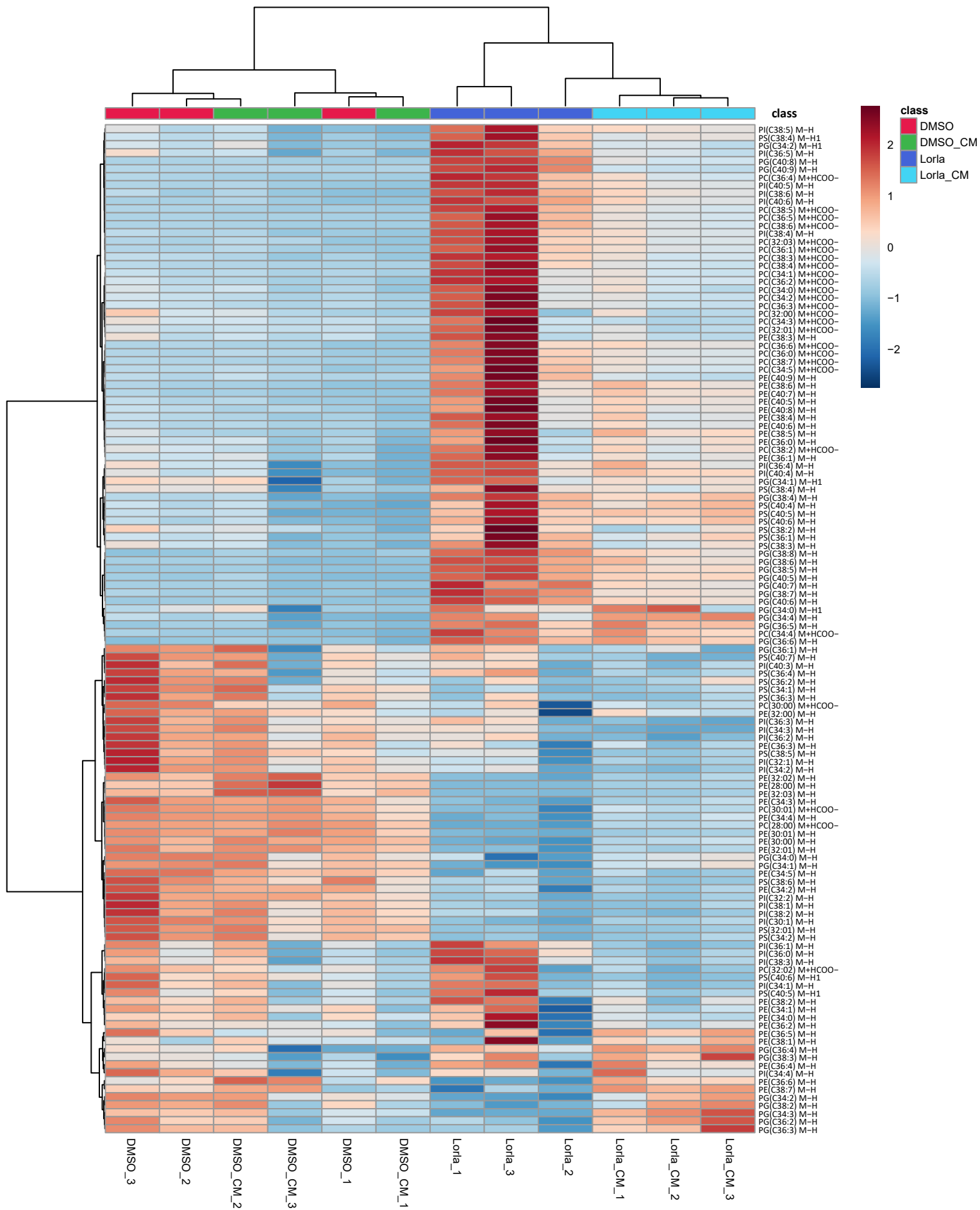

Supplement: Supplementary file 14 — Supplementary Material 14 Fig. S10: Co-cultivation with FB2-CAFs influences the expression of fatty acid metabolism-related targets upon ALK signaling perturbation via brigatinib (A) and lorlatinib (B) in H2228 and H3122 cells (n = 3). Data are presented as mean ± SD. #, p≤0.05; ##, p≤0.01; ####, p≤0.0001 compared to corresponding DMSO controls. *, p≤0.05; **, p≤0.01; ***, p≤0.001; ****, p≤0.0001 compared to brigatinib- or lorlatinib-treated mono-cultures, respectively. [file 40170_2025_400_MOESM14_ESM.pdf]

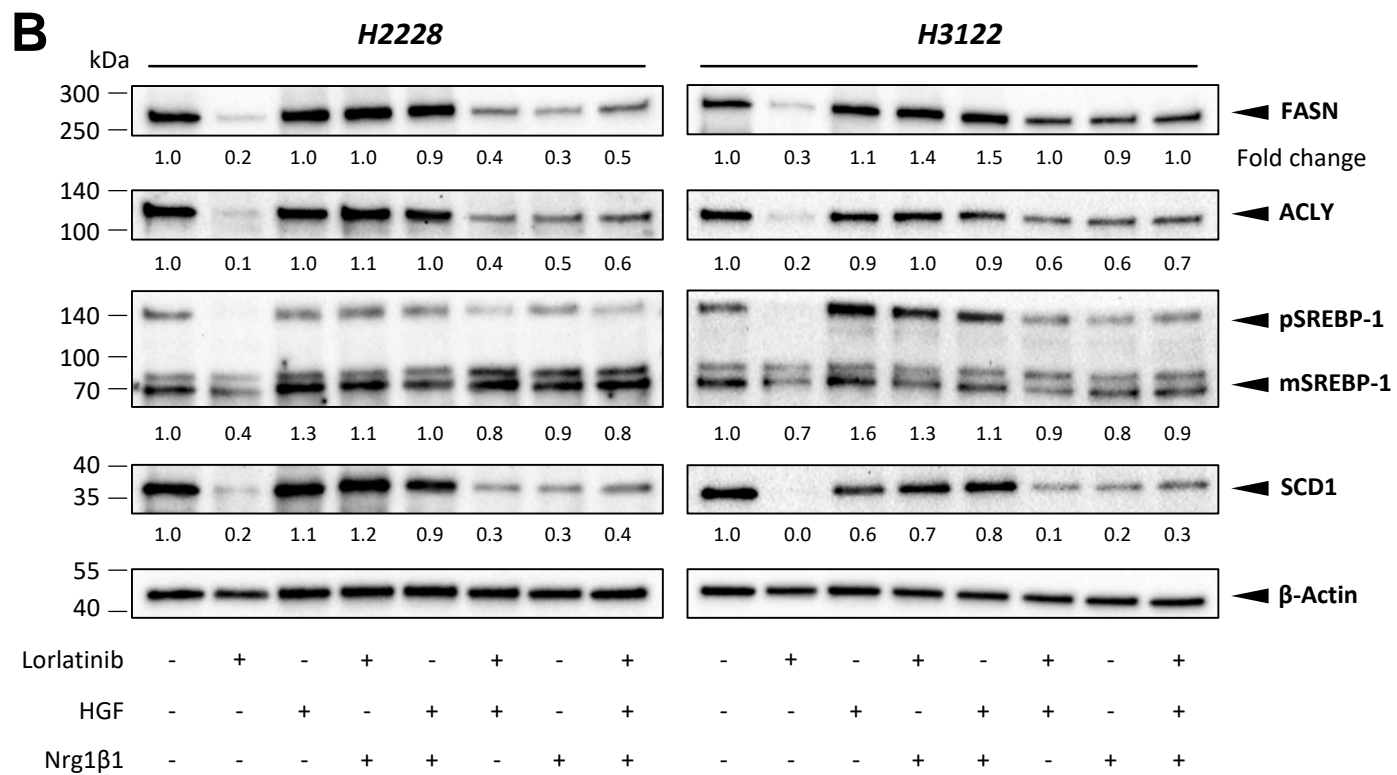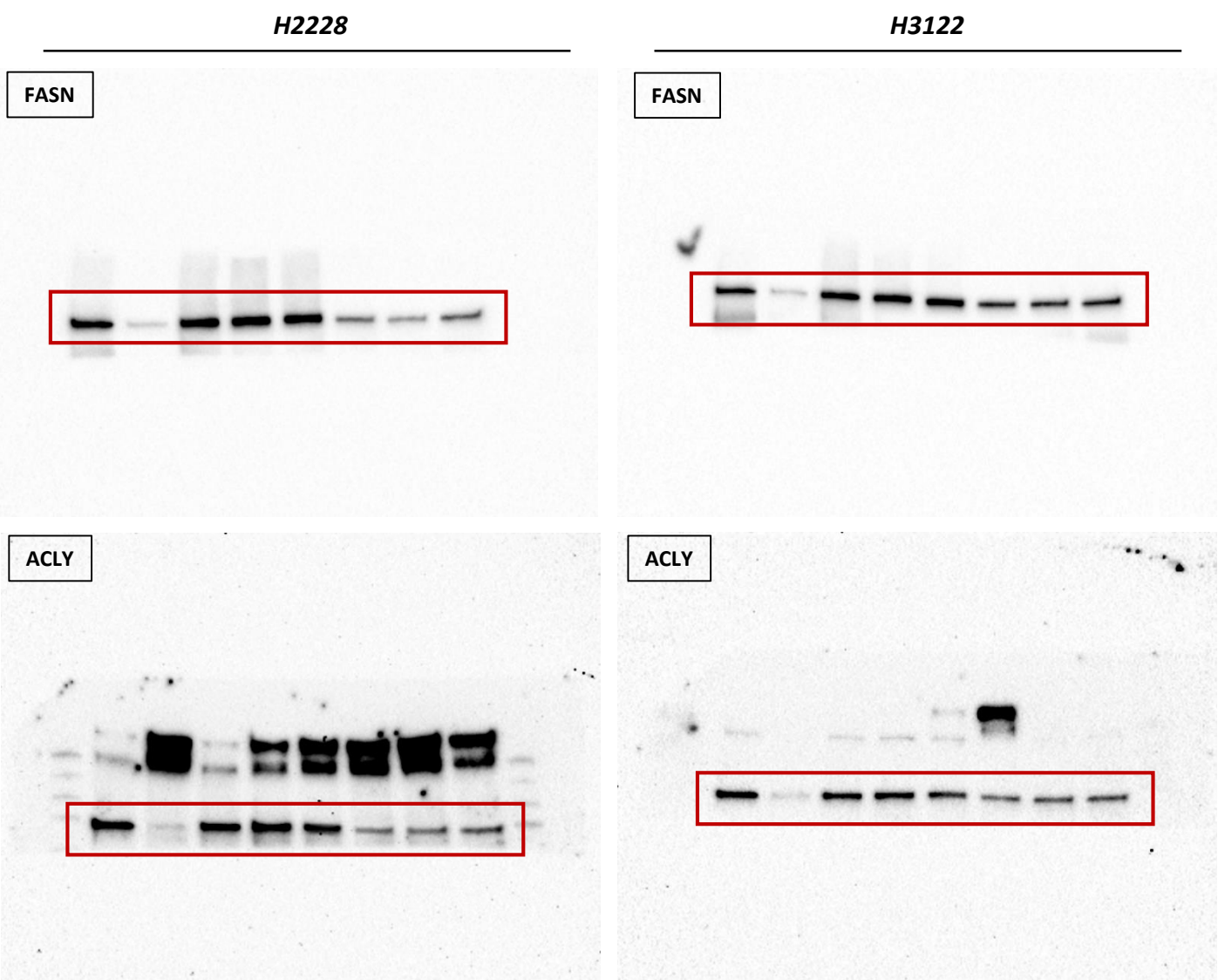

H2228

SREBP-1

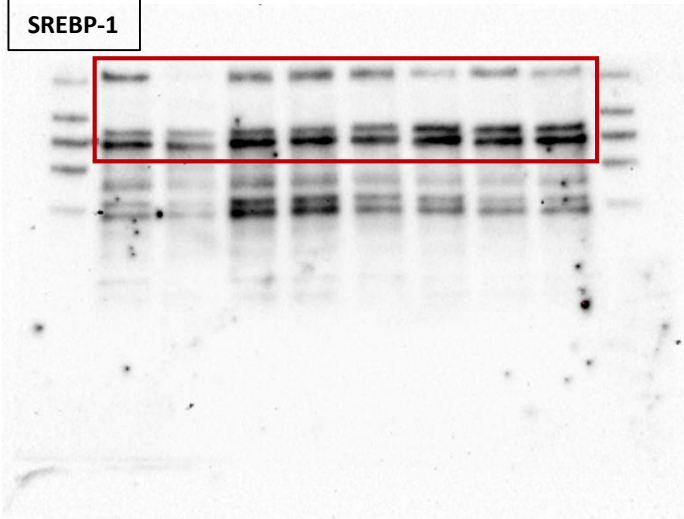

H3122

SREBP-1

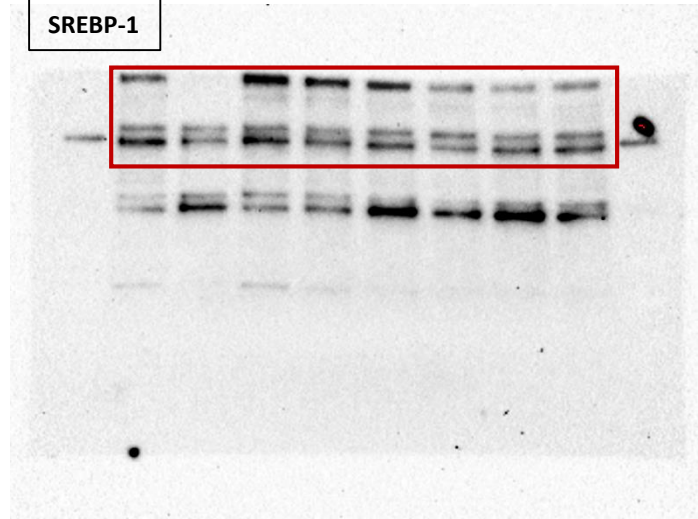

SCD1

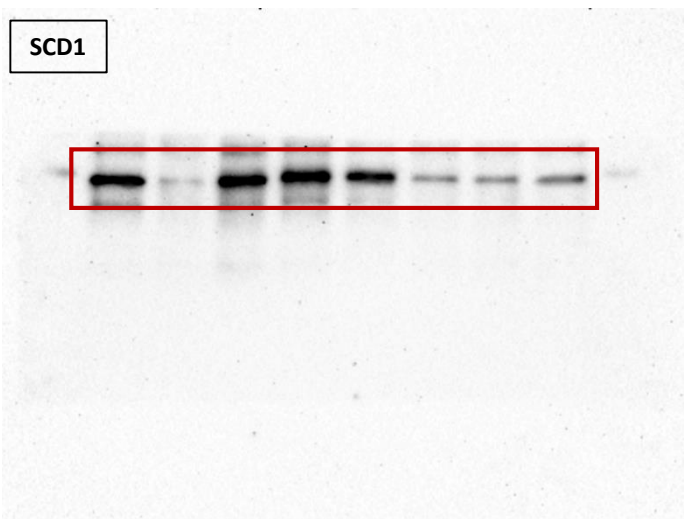

SCD1

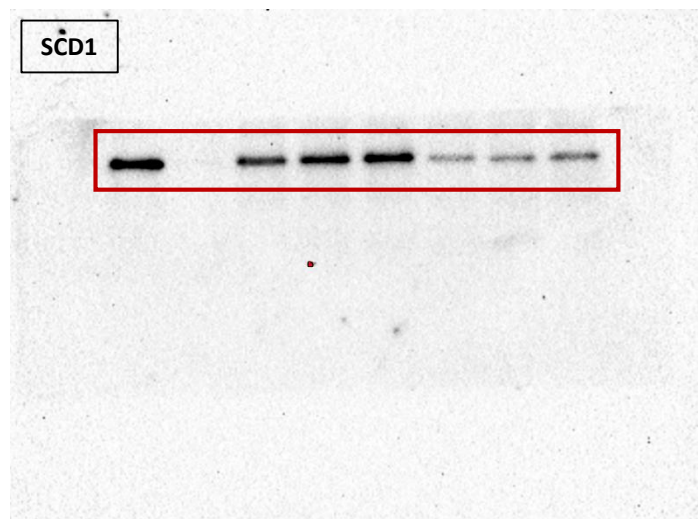

$\beta$ -Actin

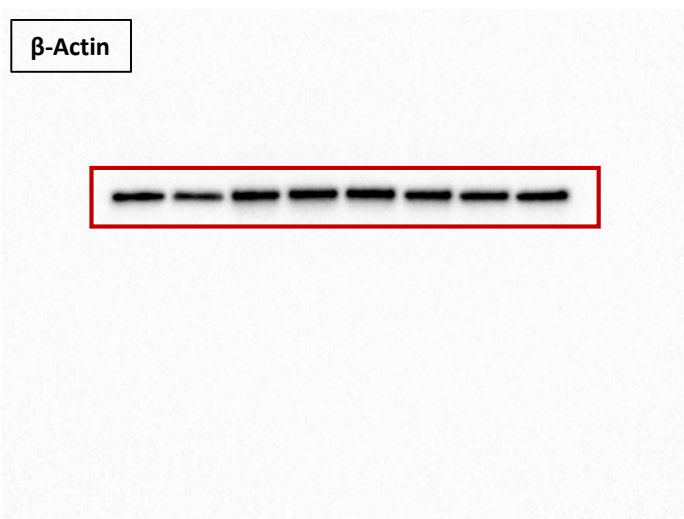

$\beta$ -Actin

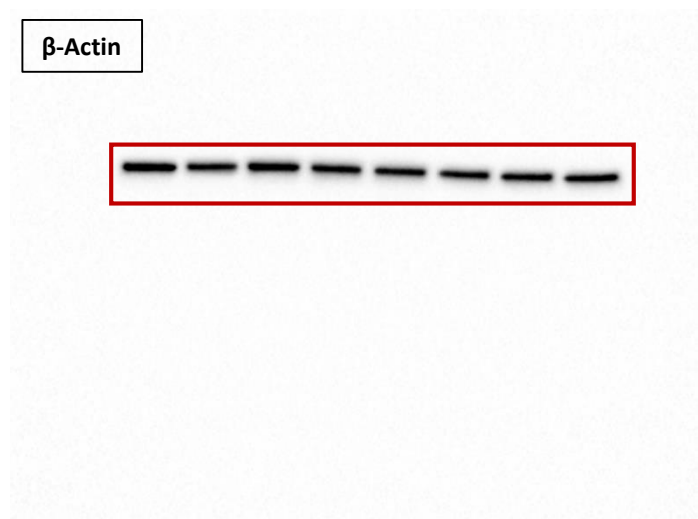

Supplement: Supplementary file 15 — Supplementary Material 15 Fig. S11: (A) Representative phase contrast images of homotypic H2228 and H3122 lung tumor spheroids treated according to the indicated conditions for 72 h. Scale bar: 200 µm. Changes in spheroid size following lorlatinib treatment in the absence or presence of FB2-CAF-conditioned medium were investigated by analyzing the spheroid areas of imaged H2228 (B) and H3122 (C) lung tumor spheroids. Spheroid size values of twelve individual spheroids are given per experimental group (n = 3). All data are presented as mean ± SD. ####, p≤0.0001 compared to DMSO controls. **, p≤0.01; ****, p≤0.0001 in comparison to lorlatinib treatment alone. [file 40170_2025_400_MOESM15_ESM.pdf]

**A**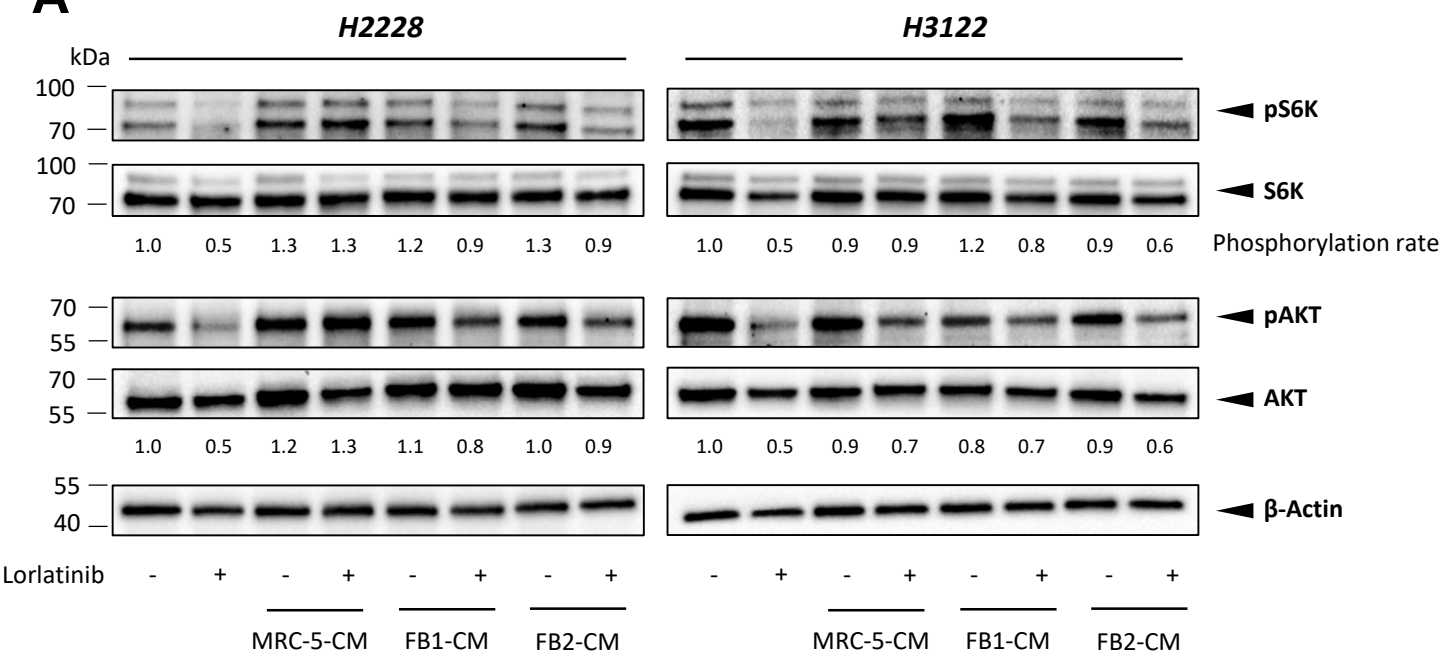**H2228**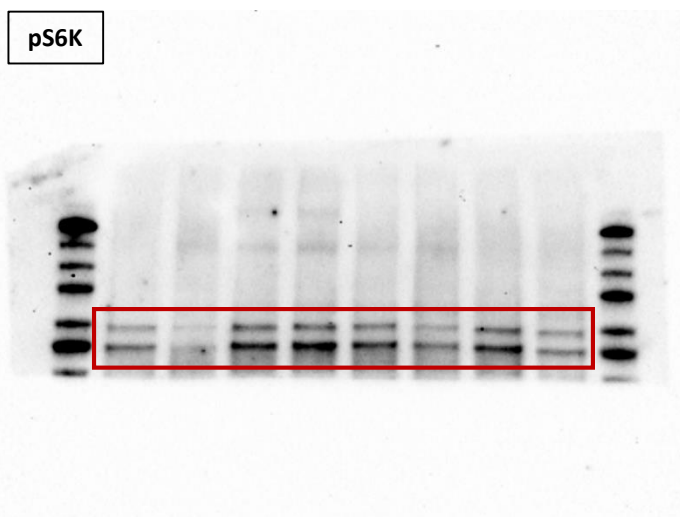**S6K**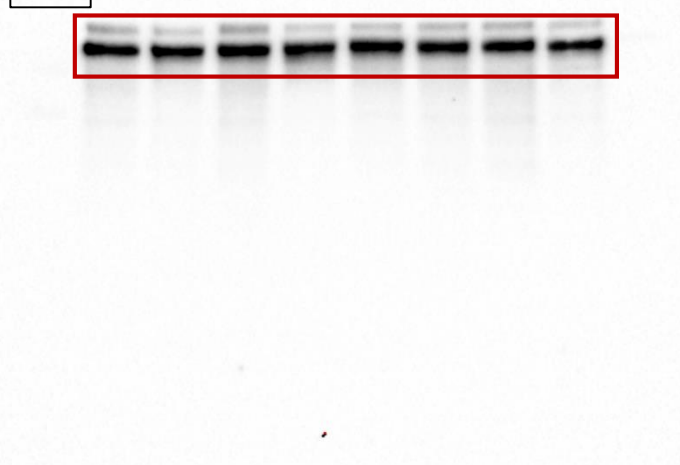**H3122**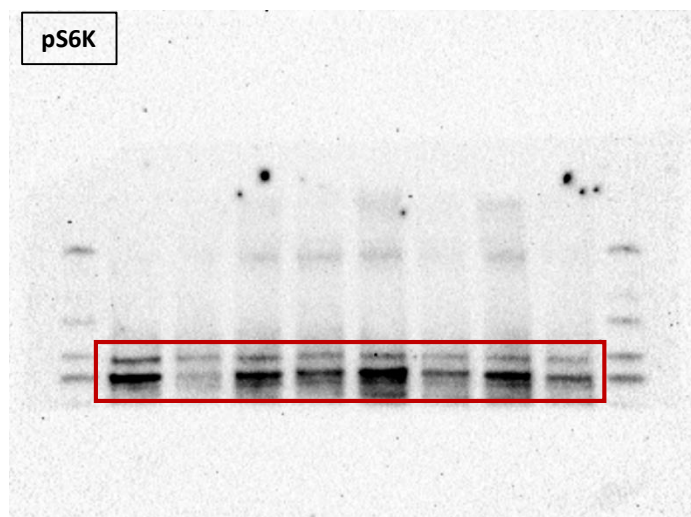**S6K**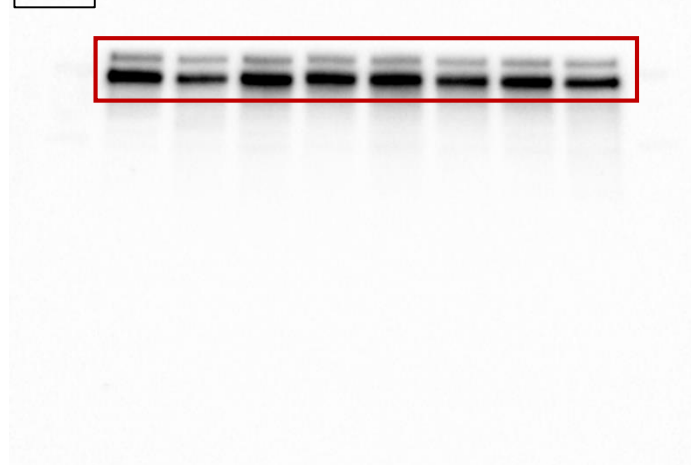

**H2228**

pAKT

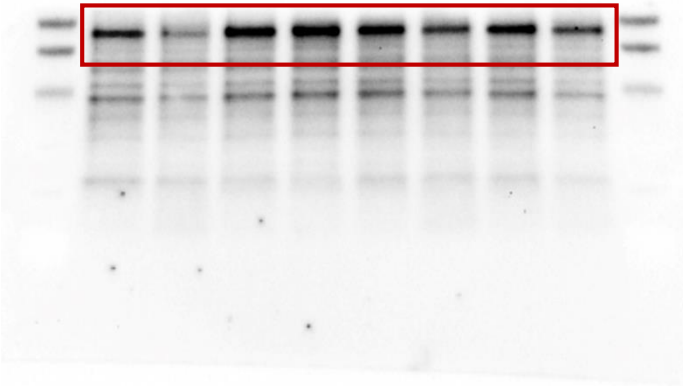

**H3122**

pAKT

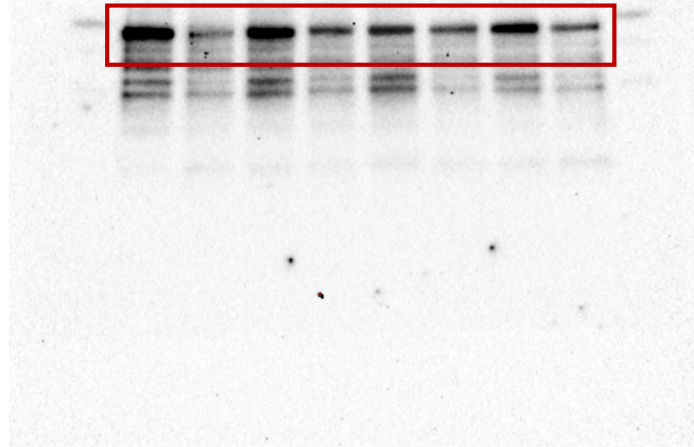

AKT

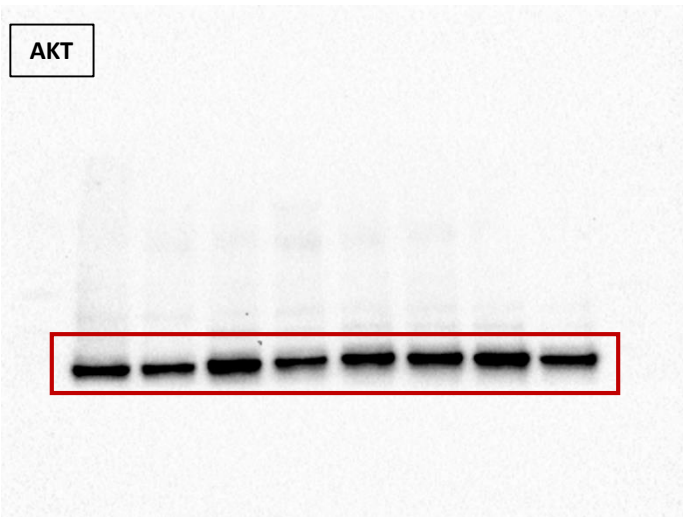

AKT

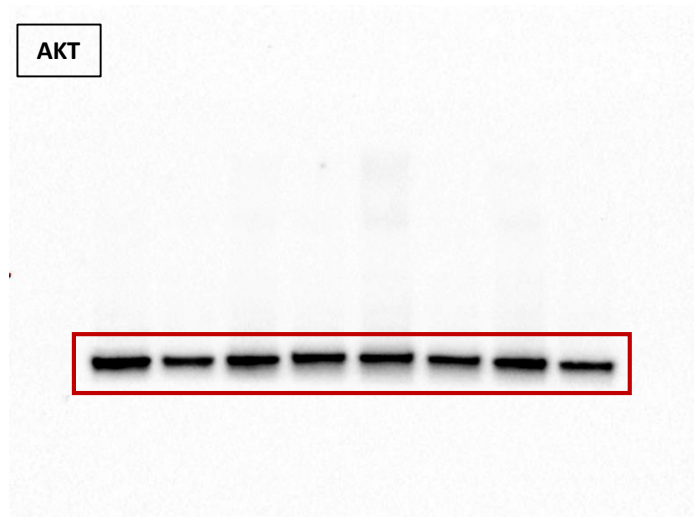

$\beta$ -Actin

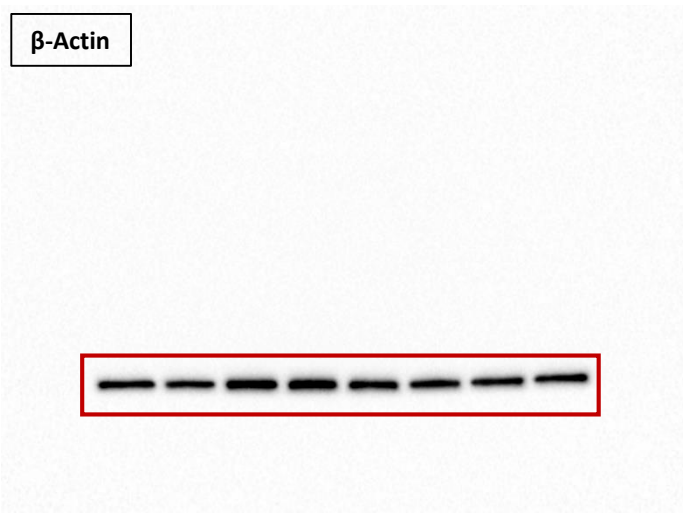

$\beta$ -Actin

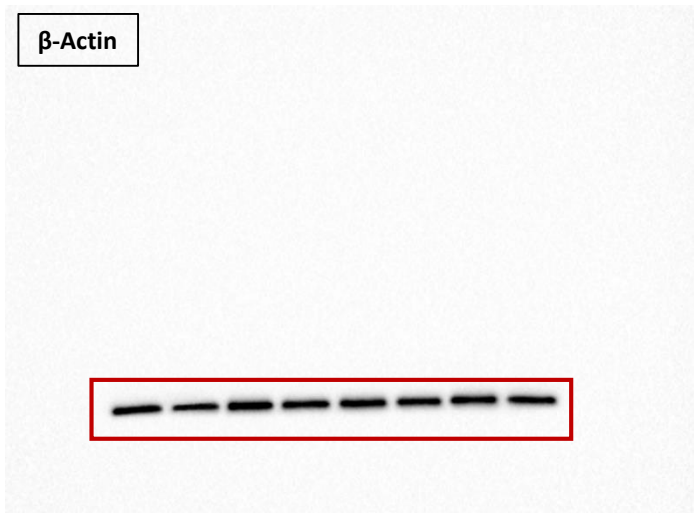

Supplement: Supplementary file 16 — Supplementary Material 16 Fig. S12: Validation of siRNA-mediated knockdown in FB2 fibroblasts using quantitative real-time PCR analysis of GAPDH, HGF, NRG1α, and NRG1β mRNA levels. FB2 fibroblasts were transfected with siRNAs targeting HGF (siHGF_1–3), NRG1 (siNRG1_1–3), a scrambled control siRNA, or a GAPDH-targeting siRNA as positive control (n = 2). Data are presented as mean ± SD. [file 40170_2025_400_MOESM16_ESM.pdf]

**A**

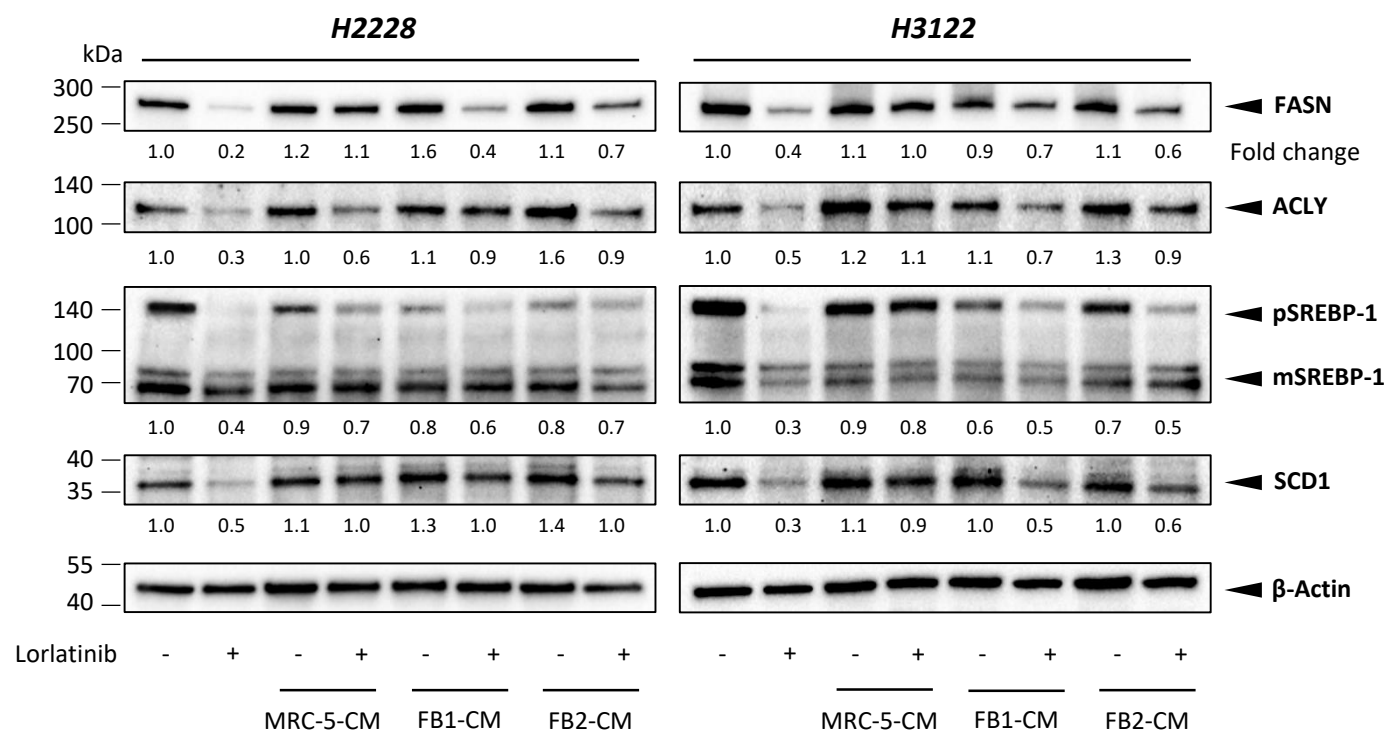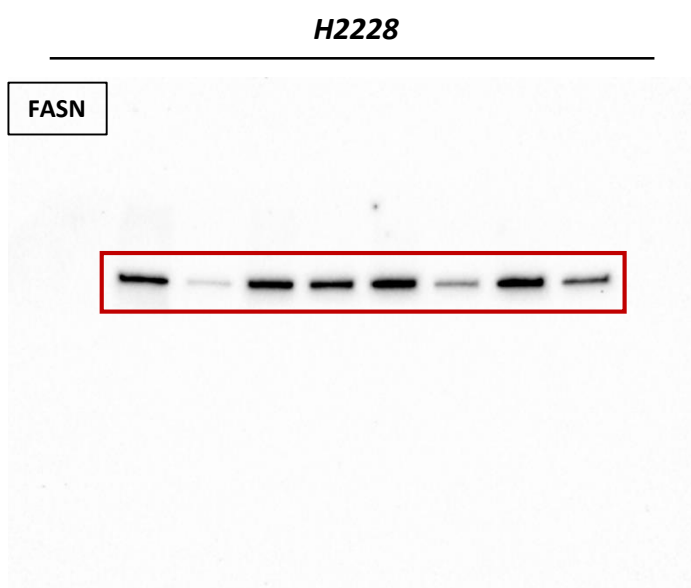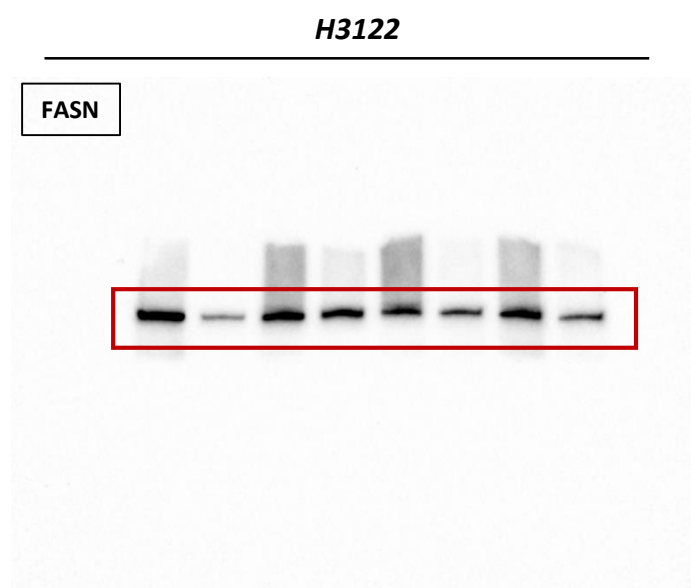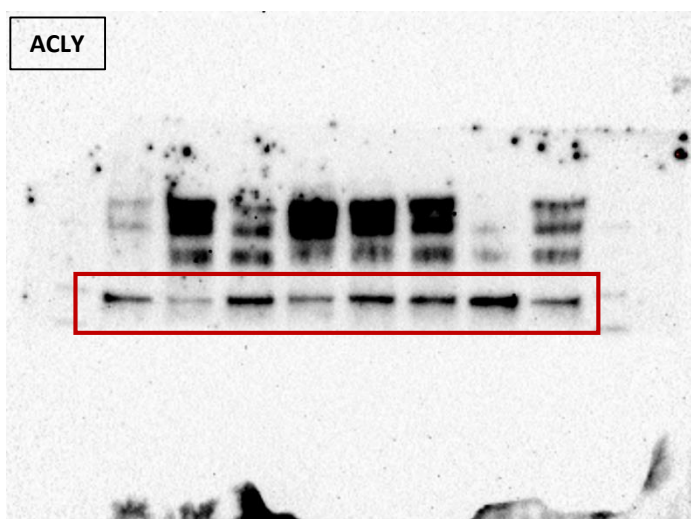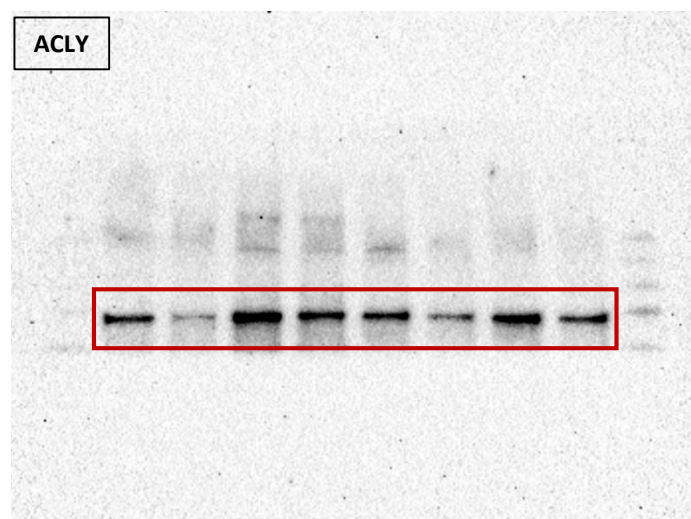

**H2228**

**SREBP-1**

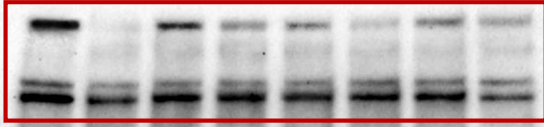

**SCD1**

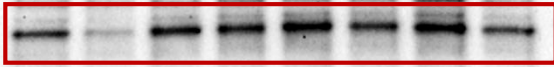

**$\beta$ -Actin**

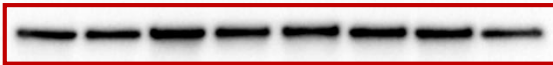

**H3122**

**SREBP-1**

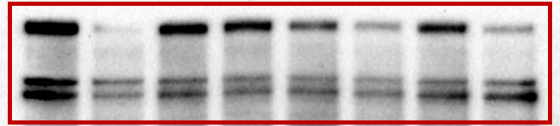

**SCD1**

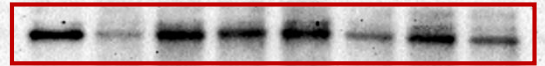

**$\beta$ -Actin**

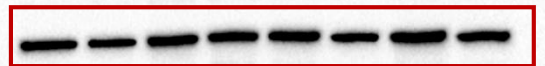

Supplement: Supplementary file 17 — Supplementary Material 17 Fig. S13: Heatmap illustrating unsupervised hierarchical clustering of lipid species abundances in H3122 spheroids across all replicates (n = 3) and treatment conditions. Data analyzed via MetaboAnalyst 5.0 (86). [file 40170_2025_400_MOESM17_ESM.pdf]

**B**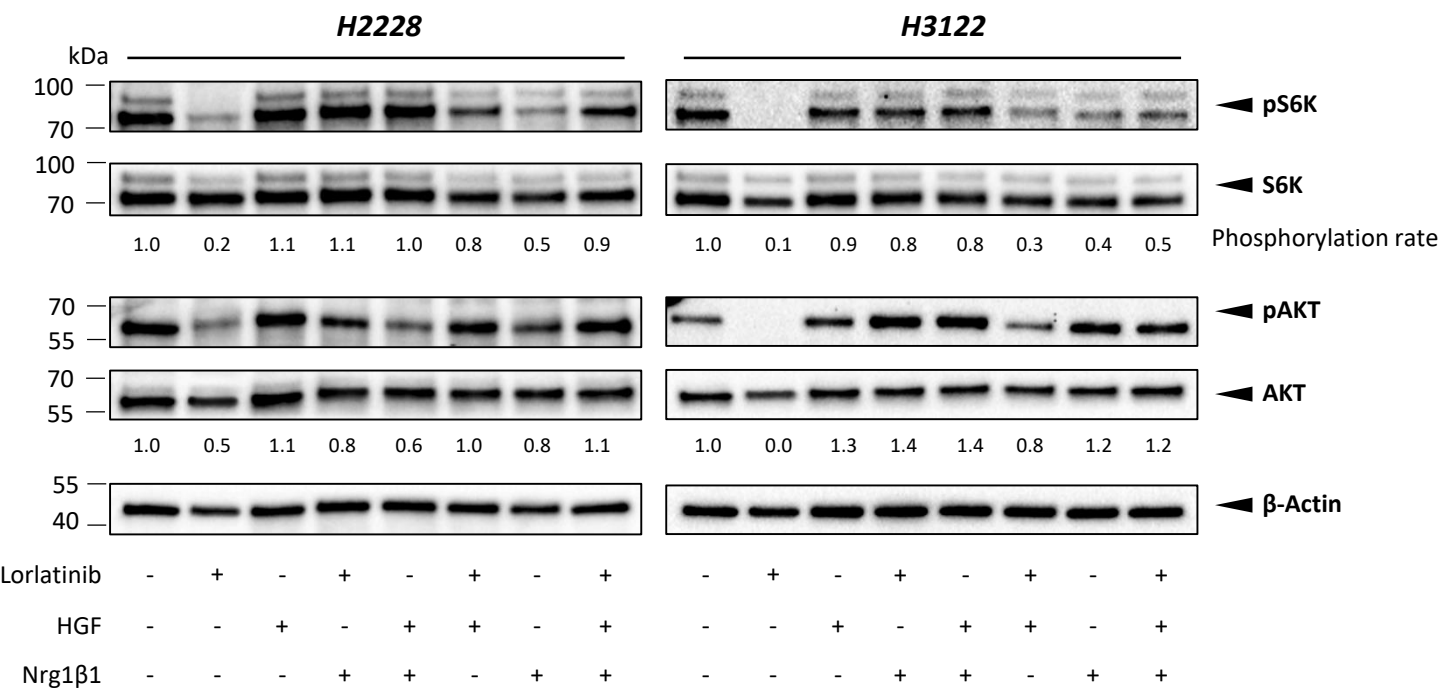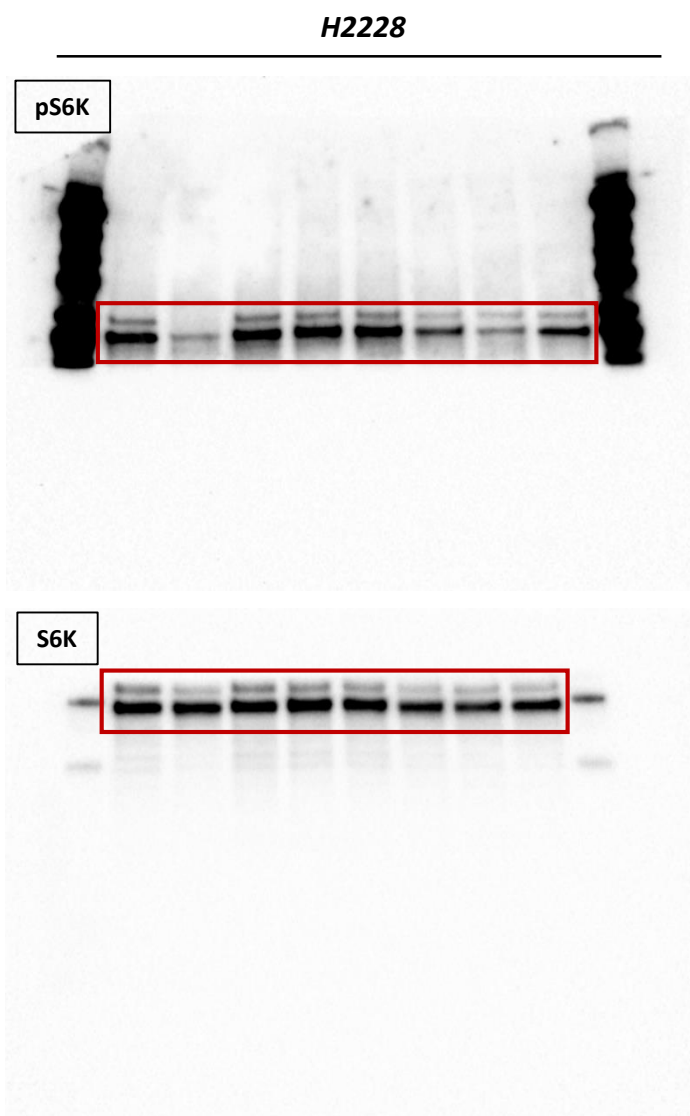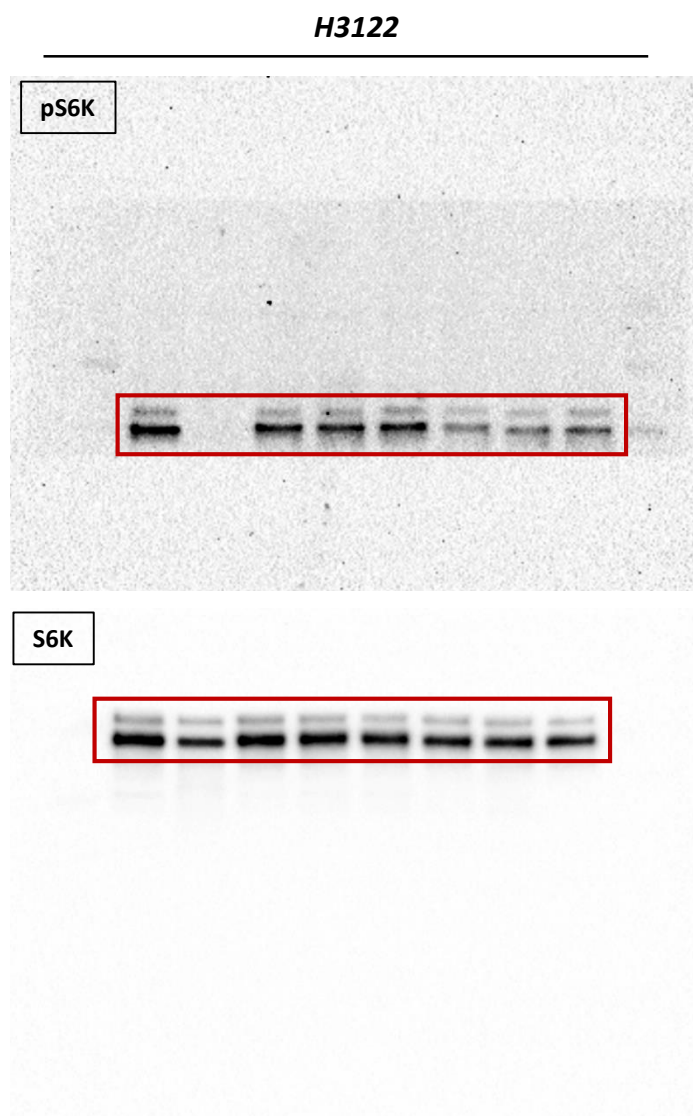

**H2228**

pAKT

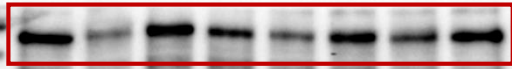

**H3122**

pAKT

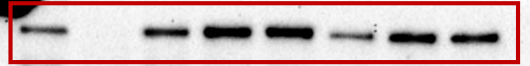

AKT

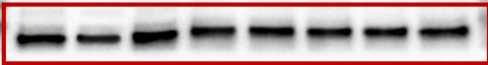

AKT

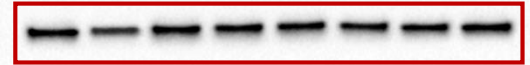

$\beta$ -Actin

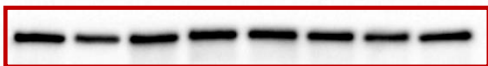

$\beta$ -Actin

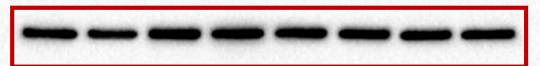

Supplement: Supplementary file 18 — Supplementary Material 18 Fig. S14: (A) Log2 ratios depicting the alterations in saturation index induced by lorlatinib. The saturation index is calculated on the basis of the sum of species with the same (un)saturation level (n = 3). (B) Cell viability analysis following the combined treatment of H2228 tumor spheroids with fatostatin, lorlatinib, and fibroblast CM (n = 3). (C) Quantification of lipid peroxidation in H2228 spheroids using C11 BODIPY. Hydrogen peroxide (H2O2) served as a positive control. All data are presented as mean ± SD. #, p ≤ 0.05; ####, p ≤ 0.0001 compared to corresponding DMSO controls. ns, not significant; *, p ≤ 0.05; **, p ≤ 0.01; ***, p ≤ 0.001; ****, p ≤ 0.0001 in comparison to lorlatinib treatment alone. [file 40170_2025_400_MOESM18_ESM.pdf]
